# Blood Flow Simulation and Uncertainty Quantification in Extensive Microvascular Networks: Application to Brain Cortical Networks

**DOI:** 10.1111/micc.70027

**Published:** 2025-09-21

**Authors:** Peter Mondrup Rasmussen

**Affiliations:** ^1^ Center of Functionally Integrative Neuroscience, Department of Clinical Medicine Aarhus University Aarhus Denmark

**Keywords:** Bayesian calibration, biophysical modeling, blood flow, boundary conditions, hemodynamic simulations, microcirculation, microvasculature, uncertainty quantification

## Abstract

**Objective:**

Microvascular blood flow simulations enhance understanding of microcirculatory phenomena at the micrometer scale by capturing heterogeneity in blood flow. However, imaged areas often only partially represent tissue regions, leading to numerous vessels crossing boundaries and strongly influencing simulated blood flows through imposed boundary conditions.

**Methods:**

Two key methodological aspects of blood flow simulations are addressed: selecting appropriate boundary conditions and quantifying the inevitable impact of boundary condition uncertainties on model simulations. An adaptive method for pressure boundary conditions is proposed and rigorously evaluated in extensive brain cortical microvascular networks. The adaptive method is integrated into a Bayesian calibration framework, inferring distributions over thousands of unknown pressure boundary conditions and providing uncertainty estimates for model simulations.

**Results:**

The adaptive method produces simulations consistent with reference data, yielding depth‐dependent pressure drop profiles and layer‐wise capillary blood flow profiles consistent with previous analysis. These hemodynamic phenomena generalize to biphasic blood flow simulation models incorporating in vivo viscosity formulations. Uncertainty quantification reveals a novel spatially heterogeneous and depth‐dependent pattern in blood flow uncertainty.

**Conclusions:**

The adaptive method for pressure boundary conditions will be useful in future applications of both forward and inverse blood flow simulations. Uncertainty quantification complements hemodynamic predictions with associated uncertainties.

**Abbreviations**
A: arteriole
ABC: adaptive boundary conditions
AL: analysis layer
AV: ascending venule
C: capillary
DA: descending arteriole
DAR: direction agreement rate
MCMC: Markov chain Monte Carlo
MCV: mean corpuscular volume
RBC: red blood cell
SA: surface arteriole
SV: surface venule
UNK: unknown
V: venule

## 1 Introduction

Microvascular networks support normal tissue function by transporting oxygen and nutrients into tissue while simultaneously removing waste products [1]. Microcirculatory variables are inherently heterogeneous, both structurally and functionally, including strong variations in red blood cell (RBC) velocities and blood oxygenation [2, 3, 4, 5]. This microscale heterogeneity, in turn, significantly influences biophysical phenomena at the macroscale, such as oxygen and nutrient delivery and waste clearance [5, 6, 7, 8, 9]. Despite their complexity, microvascular networks are well‐organized and tightly regulated to ensure an adequate distribution of blood across tissue [1, 10, 11, 12, 13, 14, 15]. However, disruption in the microcirculation can lead to altered oxygen and nutrient delivery to tissue and clearance of waste products causing cellular malfunction and diseases [16, 17], including neurodegenerative disorders [16, 18, 19, 20, 21], highlighting the need to understand microcirculatory phenomena at the network level to uncover underlying pathophysiological changes [5, 17].

Biophysical modeling [22, 23] complements in vivo studies of the microcirculation [15, 24] by enabling in silico experiments [23, 25, 26], and by enabling assimilation of model simulations with experimental data to provide insights into otherwise unobservable microcirculatory variables [2, 27, 28]. Spatially resolved microcirculatory modeling captures the heterogeneity in microcirculatory variables, providing a detailed representation of microcirculatory blood flow and solute transport phenomena at micrometer scale [2, 22]. These models are usually constructed using a combination of physical laws, empirical descriptions of the blood's complex rheological behavior, and imaging of the microvasculature [1, 29, 30, 31, 32, 33, 34]. Imaged areas, however, often only partially represent a self‐contained tissue region, leading to numerous vessels crossing the boundaries of the imaged area [35]. It is widely recognized that simulated blood flows are heavily influenced by imposed boundary conditions at these cut vessels, making the selection of appropriate boundary conditions a persistent challenge that requires considerable attention [27, 29, 30, 31, 32, 34, 35, 36, 37, 38, 39, 40]. A significant contributing factor to this challenge is the heterogeneity of pressures and blood flows throughout the microvasculature [2, 4], leading to substantial uncertainties regarding the boundary conditions [27].

Recently, we introduced a Bayesian probabilistic approach for assimilating simulations from spatially resolved blood flow models with hemodynamic observations, accounting for errors in the observed data as well as uncertainties in model parameters, including boundary conditions and parameters of rheological descriptions [27, 41]. Boundary pressures were treated as uncertain parameters, and by calibrating model predictions against in vivo measurements or literature‐derived data, the Bayesian approach offered an effective means for inferring distributions over the unknown boundary conditions while simultaneously providing a formal means for quantitatively assessing the impact of these uncertainties on model predictions.

This study continues along this line of research and addresses the complexities in modeling blood flows in extensive realistic microvascular networks, and in quantifying the impact of the inevitable boundary condition uncertainty on model predictions through the following methodology: (i) An adaptive method for setting appropriate pressure boundary conditions is proposed. (ii) The predictions made by this new method are subjected to rigorous quantitative validation in extensive rodent cortical microvascular networks, against reference model predictions based on an established blood flow simulation model [25, 32]. (iii) A probabilistic approach is adopted to assess the influence of uncertainty in pressure boundary conditions on blood flow predictions in forward simulations based on reference pressure boundary conditions and in simulations obtained with the proposed method. (iv) The new adaptive method for setting pressure boundary conditions is incorporated into our Bayesian calibration framework [27, 41], and the utility of the framework is evaluated by implementing it with three different viscosity models [42, 43, 44]. Similarity in model predictions of hemodynamic variables and in uncertainty estimates among blood flow simulation models incorporating these three viscosity models as well as their similarity with reference model predictions is evaluated. (v) Depth‐dependent pressure profiles and laminar capillary blood flow profiles are examined across models, and the depth‐resolved analysis is further complemented by an assessment of blood flow uncertainty along the underlying blood flow paths.

## 2 Materials and Methods

### 2.1 Overview of Materials and Methods

The Materials and Methods section presents the governing equations underlying the blood flow simulation model and outlines the method used for modeling nonuniform hematocrit. The adaptive method for setting pressure boundary conditions is described, and the process of integrating it into the blood flow simulation model is also described. Following this, a probabilistic approach that enables a quantitative evaluation of the impact of boundary condition uncertainty on forward blood flow simulations is described, followed by a description of our Bayesian calibration framework. Lastly, the microvascular networks and numerical experiments used in the quantitative model evaluation are described.

Throughout the text, matrices will be denoted with capital boldface letters A, vectors by small boldface letters a, scalars by small letters a, while $A_{i,j}$ will denote the element in the *i*'th row and *j*'th column in A.

### 2.2 Blood Flow Modeling

#### 2.2.1 Equations Governing Blood Flow

Blood flow simulations are based on mass conservation, and information on network topology and vessel segment morphology [1]. Following a widely used approach [1, 29, 30, 31, 32, 34, 36, 45, 46], blood is represented as an equivalent fluid [47], and empirical descriptions are adopted to account for blood's complex rheological behavior, such as variable effective viscosity with diameter and hematocrit (Fåhræus‐Lindqvist effect) [1, 7]. Based on the definition of effective viscosity [1, 7], segment blood flow rates $q\in {\mathbb{R}}^{m\times 1}$ are linearly related to node pressures $p\in {\mathbb{R}}^{n\times 1}$ by [1, 7, 29, 30, 31, 32, 34, 36, 45, 46]

(1) q=Mp

where $M\in {\mathbb{R}}^{m\times n}$ is a sparse matrix of segment hydraulic conductance (inverse resistance), which depends on segment diameter, length, and effective viscosity [1], and n and m are the number of nodes and vessel segments, respectively.

Blood flow rates at interior nodes sum to zero (mass conservation), which together with imposed pressure or flow boundary conditions, encoded in the sparse vector $b\in {\mathbb{R}}^{n\times 1}$, leads to [36]

(2) Kp=Rb

In Equation (2), K=LM+J+N, where $L\in {\mathbb{R}}^{n\times m}$ is a sparse matrix encoding segment connectivity, $J\in {\mathbb{R}}^{n\times n}$ is a sparse matrix containing ones at diagonal places corresponding to boundary nodes governed by pressure boundary conditions, $N\in {\mathbb{R}}^{n\times n}$ is a zero matrix, and $R\in {\mathbb{R}}^{n\times n}$ is an identity matrix. At this point, the two matrices N and R have no influence in Equation (2) and could be removed without loss of generality. However, their coefficients will be modified in the proposed adaptive method for pressure boundary conditions (Section 2.3.3), and the matrices are hence introduced here to maintain notational consistency. At least one boundary pressure is required to set the absolute pressure levels in Equation (2); otherwise, the system can only be determined up to a pressure constant. After imposing pressure or flow boundary conditions at boundary nodes, the system of equations in Equation (2) can be solved efficiently by numerical solvers designed for sparse linear systems [48, 49, 50]. The resulting node pressures can subsequently be substituted into Equation (1) to yield segment blood flows. Details on hydraulic conductance and on the various matrices in Equation (2), including physical units, are provided in Section 1 in File S1.

In microvascular networks, vessel segments often form a highly connected network with numerous branchpoints [1, 12] of which bifurcations are the predominant type [1]. In downstream branches of a diverging bifurcation, RBCs distribute in a nonuniform manner, the phenomenon known as the phase separation effect [1, 51]. The phase separation effect can be included in blood flow simulations by adopting empirical descriptions [1, 29, 31], resulting in nonlinear interdependence between blood flow rates and hematocrit due to the dependence of phase separation on local blood flow rates [51]. This nonlinear problem can be solved by an iterative procedure, in which the linear system, Equation (2), appears as a sub‐step [29, 30, 31].

### 2.3 An Adaptive Method for Assigning Pressure Boundary Conditions

#### 2.3.1 Model Definition

An adaptive method for assigning pressure boundary conditions is proposed. The rationale behind the method is a reproducing property of the proposed model, ensuring that statistical properties of the respective boundary conditions resemble those of a set of interior nodes. Mathematically, the pressure at boundary node i is defined relative to a weighed sum of pressures at a set of interior reference nodes

(3) $$p_{i}=\sum_{s\in N_{i}}w_{i,s}p_{s}+\Delta p_{i}$$

with $N_{i}$ being a set of interior reference nodes each with pressure $p_{s}$, $w_{i,s}>0$ are normalized weighting coefficients, that is, $\sum_{s\in N_{i}}w_{i,s}=1$, and $\Delta p_{i}$ is an imposed pressure deviation relative to the weighted sum of reference pressures.

The method thereby establishes pressure boundary conditions in relation to some average pressure characteristics of a set of interior nodes. The method's adaptive nature, which eliminates the need for a specific declaration of a reference pressure level, therefore allows it to inherently adapt to differences that may exist across microvascular networks. Simultaneously, the heterogeneity in pressures, as observed in microvascular networks [2, 30, 31, 32, 34], can be included by imposing specific pressure deviations, at individual boundary nodes, relative to this reference level.

#### 2.3.2 Defining Reference Nodes, Weighting Coefficients, and Pressure Deviations

There are many strategies for defining the set of interior reference nodes, $N_{i}$, for a given boundary node i. Reference nodes could for example be identified based on some measure of similarity, for example, node type similarity (e.g., arteriolar, venular, or capillary node), morphological similarity, or geometrical similarity (e.g., similar cortical depth or similar topological location).

There are likewise many strategies for defining the weighting coefficients w. These could, for example all be equal, for a given boundary node, and the resulting sum over reference nodes in Equation (3) would then yield the average reference node pressure. Another example could be that the weighting coefficients are defined based on some distance measure such as Euclidean distances or the shortest path‐distances between a given boundary node and the individual reference nodes.

By defining sets of interior reference nodes and corresponding weighting coefficients, the challenge of defining boundary pressures is re‐cast into a challenge of defining the pressure deviations $\Delta p_{i}$ relative to the set of reference nodes. These relative pressures could for example be sampled from some random distribution to mimic the pressure heterogeneities observed in microvascular networks, expert knowledge may be used to impose systematic deviations relative to the reference node pressures, or they could be inferred in data assimilation by using a model calibration technique [27]. In the following, the pressure deviations in Equation (3) will be referred to as *relative pressure boundary conditions* to distinguish these from conventional pressure boundary conditions.

#### 2.3.3 Incorporating the Adaptive Method Into the Governing System of Equations

The adaptive method for defining pressure boundary conditions, Equation (3), can be incorporated into the governing system of equations, Equation (2), by modifying the elements in the matrices J and N. The matrix J will contain ones at diagonal places corresponding to boundary nodes governed by either conventional pressure boundary conditions or relative pressure boundary conditions. N will encode information about the sets of interior reference nodes and their associated weighting coefficients in Equation (3) as follows. For a given boundary node i, governed by a relative pressure boundary condition, the matrix N will have nonzero elements $N_{i,s}=-w_{i,s}$ corresponding to its reference nodes $s\in N_{i}$. Finally, the relative pressure deviation $\Delta p_{i}$ will be encoded in b by $b_{i}=\Delta p_{i}$. Thus, it is straightforward to incorporate the adaptive method for pressure boundary conditions, Equation (3), into the governing system of equations, Equation (2), which can then be solved as usual.

The linear systems in Equations (1) and (2) may experience a significant reduction in sparsity if numerous boundary nodes are subjected to relative pressure boundary conditions, and many reference nodes are utilized concurrently. This decrease in sparsity can subsequently result in a substantial increase in the computational effort needed to solve the system of equations, Equation (2). However, it is often reasonable to assume that multiple boundary nodes may have identical sets of reference nodes. Even under this assumption, the pressures at these boundary nodes may vary due to the application of the relative pressure deviation $\Delta p_{i}$ at the level of individual boundary nodes. Grouping boundary nodes into sets with shared reference nodes and weighting coefficients can lead to a tremendous increase in sparsity and hence reduce computational demand significantly. Further details on how this can be implemented, by modifying elements in N and R, Equation (2), are provided in Section 2 in File S1.

### 2.4 Probabilistic Uncertainty Quantification

#### 2.4.1 Uncertainty Quantification

In the context of modeling, the blood flow simulation model can be regarded as a forward model [23, 27, 52]. When a particular set of model parameters, denoted by θ (e.g., boundary conditions), is prescribed, the model's states (e.g., segment blood flows, velocities, pressures, and hematocrits) can be predicted. By treating the uncertain parameters θ as random variables with associated probability distributions, this inherent uncertainty and the resulting uncertainty in model predictions can be captured [53, 54].

For example, considering segment blood flows as the variable of interest, the probabilistic approach enables assessment of how uncertainties in boundary conditions translate into uncertainties in these blood flows. Formally, the probability distribution that governs segment blood flows, given the uncertainty in boundary conditions, can be inferred and summarized by various summary statistics. The expectation

(4) $$m=E_{\theta }\left[q\left(\theta \right)\right]$$

provides a measure of central tendency, representing the average or expected value of individual segments' blood flow under uncertainty. The covariance matrix

(5) $$\Omega =E_{\theta }\left[\left(q\left(\theta \right)-m\right){\left(q\left(\theta \right)-m\right)}^{\prime }\right]$$

on the other hand, quantifies the spread or dispersion around this expected value, thereby capturing the inherent uncertainty in blood flows. Its off‐diagonal elements convey information on between‐segment covariance, while its diagonal elements provide insight into individual segments' variance. Consequently, the square root of the j’th diagonal element in Ω corresponds to the standard deviation $\sigma_{j}$ governing the simulated blood flow in segment j.

A large standard deviation for a given segment, relative to its mean, indicates high uncertainty in blood flow, and blood flow may even exhibit opposite directions under this uncertainty. The following metric, termed the direction agreement rate (DAR), was established to capture this effect and is defined by

(6) $$\mathrm{DA}R_{j}=100\times \max\left(P\left(q_{j}\left(\theta \right)>0\right),1-P\left(q_{j}\left(\theta \right)>0\right)\right)$$

Here, $P\left(q_{j}\left(\theta \right)>0\right)$ is the probability that the segment's blood flow, which is a random variable, follows the direction from the segment's start node to its end node. The DAR metric thereby quantifies the rate at which a segment's blood flow direction agrees with its predominant direction. A large DAR, close to 100%, indicates that a segment's blood flow is governed by a small dispersion, corresponding to a small standard deviation $\sigma_{j}$ relative to the mean $m_{j}$. Conversely, a small DAR, close to 50%, occurs with large dispersion. The DAR metric thus provides a straightforward, yet insightful and interpretable means for quantitively assessing the uncertainties in blood flow simulations. It is noted that DAR is a statistical measure independent of observed blood flow direction. Instead, DAR quantifies consistency in the simulated blood flow direction under uncertainty.

The probabilistic approach to uncertainty quantification depends on methods to determine the distribution governing segment blood flows, considering uncertainty in boundary conditions. The following two sections will describe methods for inferring this distribution. First, a simplified approach will be applied to linear forward simulation models, followed by a more comprehensive method, anchored in Bayesian statistics, which takes observed data or literature‐derived data into account.

#### 2.4.2 Uncertainty Quantification in Linear Forward Modeling

Assuming that specific boundary conditions have been defined, for example from measured [2] or literature data [2, 29, 31, 32], through domain‐specific reasoning [29, 31], by numerical optimization and calibration techniques [27, 36, 38, 55], or by embedding techniques [32], the objective of the following is to establish a straightforward approach to quantify the extent to which uncertainties in these imposed boundary conditions lead to uncertainties in the simulated blood flows.

The governing equations (Equations 1 and 2) can be combined to yield

(7) q=Hb

where $H=MK^{-1}R$. Under the simplifying assumption of a known hematocrit field, (e.g., uniform hematocrit [28, 35, 56]), the mathematical formulation in Equation (7) emphasizes that segment blood flows are linear transformations of the pressure or blood flow boundary conditions in b, a property will be utilized in the following.

Let the set of row indices corresponding to boundary nodes in b be partitioned into two sets A and B. These two sets consist of boundary nodes that the investigator wants to study for their impact of uncertainty and boundary nodes for which the investigator does not want to study for the impact of uncertainty, respectively. Equation (7) can then be written as

(8) $$q=H_{A}b_{A}+H_{B}b_{B}=H_{A}b_{A}+q_{B}$$

where $H_{x}$ and $b_{x}$ are composed of columns and rows in H and b, respectively, corresponding to the indices contained in A and B. From a probabilistic perspective, assume that the uncertainty governing the boundary conditions at nodes in A is modeled by a Gaussian distribution with mean $b_{A}$ and covariance $\Sigma_{A}$, that is, $N\left(b_{A},\Sigma_{A}\right)$. That is, the expected values of these boundary conditions are the same as defined in b, whereas the additional boundary condition uncertainties are described in terms of the covariance matrix $\Sigma_{A}$. It follows from statistical theory [53], that variables resulting from a linear transformation of Gaussian variables, Equation (8), are also governed by a Gaussian distribution, that is.

(9) $$q\sim N\left(m,\Omega \right)$$

Segment blood flows are thereby random variables with the expected value

(10) $$m=H_{A}b_{A}+q_{B}$$

identical to the blood flows in Equations (7) and (8). The additional uncertainties in blood flows, induced by the uncertainties in boundary conditions, are described by the covariance matrix

(11) $$\Omega =H_{A}\Sigma_{A}H_{A}^{\prime }$$

In a microvascular network, a certain degree of covariance between boundary condition variables is expected due to the network's structural connectivity. However, this covariance information is complex and not readily accessible. Instead, a pragmatic approach, which was employed in this study, is to treat the covariance matrix $\Sigma_{A}$ as a diagonal matrix. Hence, boundary condition uncertainties are described in terms of uncertainty related to individual boundary variables. Although this is a simplification, it is important to note that the covariance matrix Ω, which governs the distribution of segment blood flows in Equation (9), accumulates uncertainty contributions from all individual boundary variables by filtering these through the matrix $H_{A}$ as seen in Equation (11). The probabilistic approach to uncertainty quantification, as outlined above, is related to classical sensitivity analysis, Section 5 in File S1.

#### 2.4.3 Bayesian Model Calibration and Uncertainty Quantification in Inverse Modeling

Inverse modeling involves assimilating model simulations with observed data to infer latent model states and the underlying but unknown parameters. In the present context, it amounts to inferring latent hemodynamic variables and the unknown pressure boundary conditions based on an incomplete and noisy set of measured or literature‐derived blood flow rates or velocities. Compared with forward modeling, this process is inherently more complex due to the need to solve for multiple parameters that could potentially explain the observed data and the nonuniqueness of the solution [27, 52]. The Bayesian approach, offering a statistically rigorous framework for confronting these challenges [54], employs Bayes' rule.

(12) $$p\left(\theta |y\right)=\frac{p\left(y|\theta \right)p\left(\theta \right)}{p\left(y\right)}$$

where $p\left(\theta |y\right)$ is the posterior probability of the parameters θ given the observations in y, $p\left(y|\theta \right)$ is the likelihood which is the probability of the observations given the model simulations, $p\left(\theta \right)$ is the prior which is the probability of the parameters before seeing the observations, and $p\left(y\right)$ is the marginal likelihood which is constant for a given set of observations.

From a practical perspective, the Bayesian approach, as outlined above, requires the choice of likelihood and prior distributions in Equation (12). Often, the posterior distribution, Equation (12), does not have an analytical solution, which leads to the application of advanced techniques such as Markov chain Monte Carlo methods [27, 54] for numerically computing the posterior distribution, along with summary statistics such as the expectation, Equation (4), the covariance, Equation (5), and the direction agreement rate, Equation (6). These practical considerations are further detailed in Section 2.6.2.

### 2.5 Microvascular Networks

**FIGURE 1:**
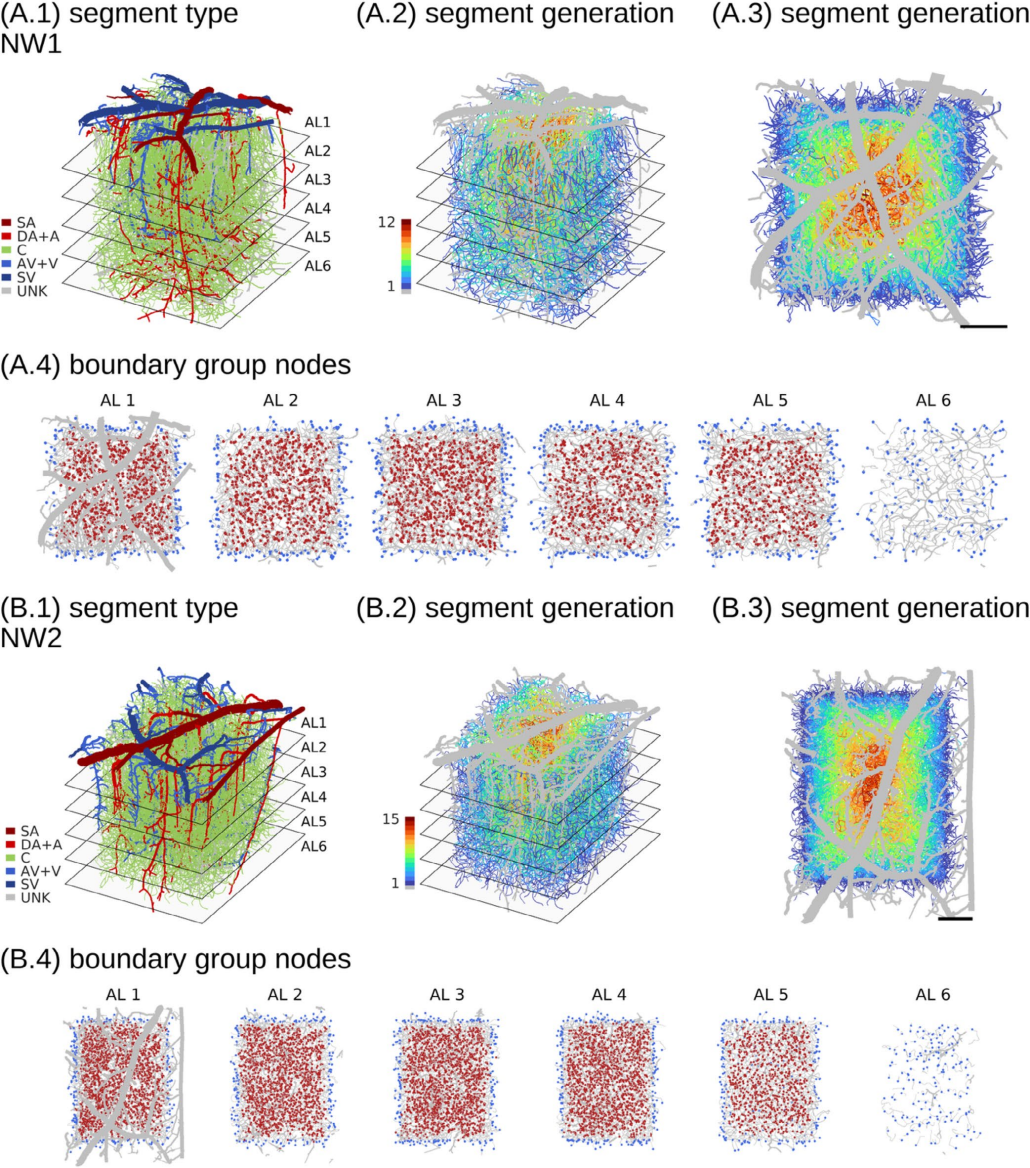
Segment types and segment generations for the two microvascular networks, NW1 and NW2. In (A.1, B.1), each segment is uniquely colored according to its category. In (A.2, B.2) and (A.3, B.3), the generation of capillary segments relative to network boundary nodes is color‐coded, with non‐capillaries shown in gray to emphasize capillary vessels. The first two columns present 3D (xyz) representations of the networks, while the third column provides 2D (xy) projections. Planes in the depth direction, visible in the first two columns, mark the boundaries between analysis layers (ALs), with layer boundaries separated by 200 μm. Scale bars in (A.3, B.3) represent 200 μm. In (A.4, B.4), boundary nodes of categories C and UNK are colored blue, interior reference nodes are red, and vessel segments are gray. Some segments appear without both end‐nodes since these nodes were located outside the respective analysis layers.

The analysis was conducted using extensive microvascular networks from the mouse somatosensory cortex [33, 57, 58]. Variants of these networks, including blood flow simulations, subsequently made accessible by Schmid et al. [32, 59], and downloaded from https://doi.org/10.5281/zenodo.758632 and https://doi.org/10.5281/zenodo.5115639 under the CC BY 4.0 license (https://creativecommons.org/licenses/by/4.0/), were used in this study. The two networks, which are available in both data sources [32, 59], were considered. These anatomically accurate networks represent volumes of approximately 1.5–2.2 cubic mm from the pial surface to a depth of around 1.2 mm. Vessels were categorized into six classes based on their diameter and their connectivity with surface vessels of known classes [32]. Vessel categories included surface arterioles (SA), descending arterioles and arterioles (DA + A), capillaries (C), ascending venules and venules (AV + V), surface venules (SV), and unknown (UNK). The two networks are visualized in Figure 1A.1,B.1 and characteristic morphological parameters are summarized in Table 1 in File S2.

Schmid et al. simulated the distribution of pressure, blood flow, RBC velocity, and hematocrit throughout the networks using a blood flow simulation model that tracks discrete RBCs [32], and these simulations, denoted ref.Data, was used as a reference for evaluating the impact of boundary condition uncertainty on model simulations and in the quantitative assessment of the proposed adaptive method for setting pressure boundary conditions.

The networks, which essentially represent cubes cut from cortex, have numerous cut vessels on all six cube faces, except at the pial face. The latest version of the networks [59] features a somewhat simplified representation of vessel segments near network faces, and the original networks [32] were trimmed accordingly in this study, with details provided in Figure 1 in File S3. The number of boundary nodes for surface arterioles and surface venules is in the tens, but this number rapidly increases with depth, resulting in a large total number of boundary nodes: 1225 for the smaller network (NW1) and 1751 for the larger network (NW2), Table 1.

**TABLE 1:** Segment and node information on the two microvascular networks (NW1 and NW2).

Network	Segments	Nodes	Boundary nodes						
			SA	DA + A	C	V + AV	SV	UNK	Total
NW1	10 873	7928	8	69	961	46	6	135	1225
NW2	19 318	13 707	8	89	1405	181	11	57	1751

Abbreviations: A, arteriole; AV, ascending venule; C, capillary; DA, descending arteriole; SA, surface arteriole; SV, surface venule; UNK, unknown; V, venule.

In this study, a straightforward segment generation metric was further assigned to individual vessel segments. This metric, designed to be purely topological in nature, was defined in relation to boundary nodes and calculated using the following algorithm. Initially, a set of nodes was identified from all boundary nodes. Following this, a new set of nodes was identified, which could be reached by propagating along segments connected to the initial set of nodes. These two node sets were then merged, and all segments with both their end‐nodes contained in this combined set were assigned a generation level of one. A new iteration was then initialized from the combined set of nodes to identify segments with a generation level of two. This iterative procedure was repeated until a segment generation was assigned to all vessel segments, resulting in segment generations up to 12 (NW1) and 15 (NW2), Figure 1A.2/3 and 1B.2/3.

Groups of interior capillary nodes were identified as reference nodes in the proposed adaptive method for pressure boundary conditions. Each network was first divided into six analysis layers (ALs) along the depth direction, with layer boundaries separated by 200 μm [32]. For AL 1–5, groups of reference nodes were then identified as the set of nodes that were end‐nodes of capillary segments with a segment generation level of at least two to reduce the influence of boundary effects. In NW2, the deepest analysis layer, AL 6, contained only a small number of candidate reference nodes, Figure 1 in File S3. Therefore, no reference group was defined based on nodes in AL 6. Instead, the group of reference nodes in AL 5 was also utilized as reference nodes for boundary nodes in AL 6. The same approach was applied to NW1 to maintain processing consistency across networks. Individual reference node groups contained 661–1071 (NW1) and 922–2287 (NW2) nodes, Table 2 in File S2. The groups of reference nodes and associated boundary nodes are visualized in Figure 1A.4,B.4.

Individual segments were assigned to a unique AL based on their average endpoint depth, and hemodynamic metrics were analyzed for AL 1–5 [32].

### 2.6 Numerical Experiments

#### 2.6.1 Experiment 1—Forward Modeling

The first numerical experiment was designed to assess the impact of boundary condition uncertainty on model simulations and to quantitatively evaluate the proposed adaptive method for pressure boundary conditions. The experiment employed segment hematocrits and boundary node pressures from the reference dataset, ref.Data. To exclusively evaluate the impact of pressure boundary conditions, the following strategy was adopted. Segment hematocrits from ref.Data were used to compute the effective viscosities of individual segments. These viscosities were subsequently used to calculate the hydraulic resistances, which were then kept constant throughout the simulations, enabling a direct assessment of the influence of boundary pressures parameterization across models. Consistent with the reference analysis [32], viscosities were calculated using an in vitro viscosity formulation [42], Table 2, plasma viscosity was set to 1.2 cP, and no adjustment was made for differences in mean corpuscular volume (MCV) between humans and mice. Details on the viscosity model are provided in Section 3.2.1 in File S1.

**TABLE 2:** Model nomenclature.

Model	Viscosity model	Pressure boundary conditions	Segment hematocrit
ref.Vitro	In vitro, Pries et al. [42]	All defined from pressures in ref.Data.	Nonuniform and defined from ref.Data [32].
ref.Vitro.ABC	In vitro, Pries et al. [42]	Adaptive method for pressure boundary conditions used for vessel categories C and UNK. Pressures from ref.Data used for all other vessel categories.	Same procedure as in ref.Vitro
cal.Vitro.ABC	In vitro, Pries et al. [42]	Adaptive method for pressure boundary conditions used for vessel categories C and UNK. All pressure boundary conditions, including relative pressure boundary conditions, inferred by Bayesian calibration.	Uniform during Bayesian calibration. Subsequently nonuniform by using the inferred boundary pressures in a nonlinear simulation model incorporating the phase separation model by Pries et al. 1989 [51].
cal.Vivo.ABC	In vivo, Pries et al. [44]	Same procedure as in cal.Vitro.ABC.	Same procedure as in cal.Vitro.ABC.
cal.Esl.ABC	In vivo incorporating the effect of the endothelial surface layer (ESL), Pries and Secomb 2004 [43]	Same procedure as in cal.Vitro.ABC.	Same procedure as in cal.Vitro.ABC.

*Note:* Summary of the two reference models and three calibration models studied. Original references are included for the various viscosity models and the phase separation model. Sections 3 and 4 in File S1 provide details on mathematical formulations, implementation details, and additional references.

Two models were examined and are referred to as *reference models* throughout the text, Table 2. In the first model, ref.Vitro, model simulations were performed using node pressures from ref.Data as pressure boundary conditions for all boundary nodes. In the second model, ref.Vitro.ABC, node pressures from ref.Data were used as boundary pressures for vessels categories SA, DA + A, AV + V, and SV, while boundary conditions for vessel categories C and UNK were assigned using the proposed adaptive method, Table 2. Specifically, the pressure boundary condition for a given boundary node (in a given AL) was assigned relative to reference nodes in the respective layer (Figure 1A.4,B.4). Equal weighting coefficients were assigned to individual reference nodes, and the relative boundary pressures were thus defined relative to the average layer‐wise reference node pressure. The relative boundary pressures $\Delta p_{i}$ in Equation (3) were defined by sampling each of these i.i.d. from a Gaussian distribution with zero mean and a standard deviation of 4 mmHg. Thereby, heterogeneity in boundary pressures was induced by this random assignment of pressure deviances, while consistency between the layer‐wise average boundary node pressure and interior reference node pressures was ensured at the same time.

The probabilistic approach to uncertainty analysis in forward models, Section 2.4.2 Equation (9), was adopted to examine the influence of pressure boundary condition uncertainty on blood flow uncertainty. Blood flow uncertainty was quantified by the direction agreement rate, Equation (6). A standard deviation of 4 mmHg was used to represent boundary pressure uncertainty. Uncertainty was exclusively applied to boundary vessels of category C and UNK to focus the analysis on how blood flow was influenced by uncertainties associated with boundary pressures that differed across the ref.Vitro and ref.Vitro.ABC models. In Experiment 2, a more advanced strategy was adopted, which included boundary condition uncertainty for all vessel categories.

#### 2.6.2 Experiment 2—Inverse Modeling

In the second numerical experiment, the adaptive method for pressure boundary conditions was integrated into our Bayesian calibration framework [27]. This integration aimed at inferring probability distributions over all boundary pressures and simultaneously at quantifying the impact of the resulting uncertainty on blood flow simulations.

The framework's utility was assessed by implementing the blood flow simulation model with three different viscosity formulations, all of which are currently used in blood flow simulations in brain cortex [27, 30, 31, 35, 37]: an in vitro viscosity formulation [42], cal.Vitro.ABC model, and two in vivo viscosity formulations [43, 44], cal.Vivo.ABC and cal.Esl.ABC models, Table 2. Mathematical details of three empirical models of effective viscosity [42, 43, 44] used are available in Sections 3.2.1 to 3.2.3 in File S1. The three resulting model variants are referred to as *calibration models* in the text. Plasma viscosity was set at 1.2 cP, and adjustments for the differences in MCV between humans, 92 fL [1], and mice, 45 fL [60], were included as described in Section 3.1 in File S1. The phase separation effect was additionally included to account for varying hematocrit throughout the networks, Table 2. Further details on the specific phase separation model [31, 41, 43, 51] used and on algorithmic details of the iterative procedure can be found in File S1 Sections 3.3 and 4, respectively.

To reduce computational costs, a two‐step procedure was established. During Bayesian calibration, a uniform discharge hematocrit of 40% was assigned to all segments [35, 56] and held constant [32]. Bayesian calibration yielded samples from the posterior distributions governing the pressure boundary conditions, Equation (12), and subsets of these samples were subsequently propagated through nonlinear simulation models incorporating the phase separation effect using a uniform inlet discharge hematocrit [32] of 40% at boundary nodes [35, 37].

**FIGURE 2:**
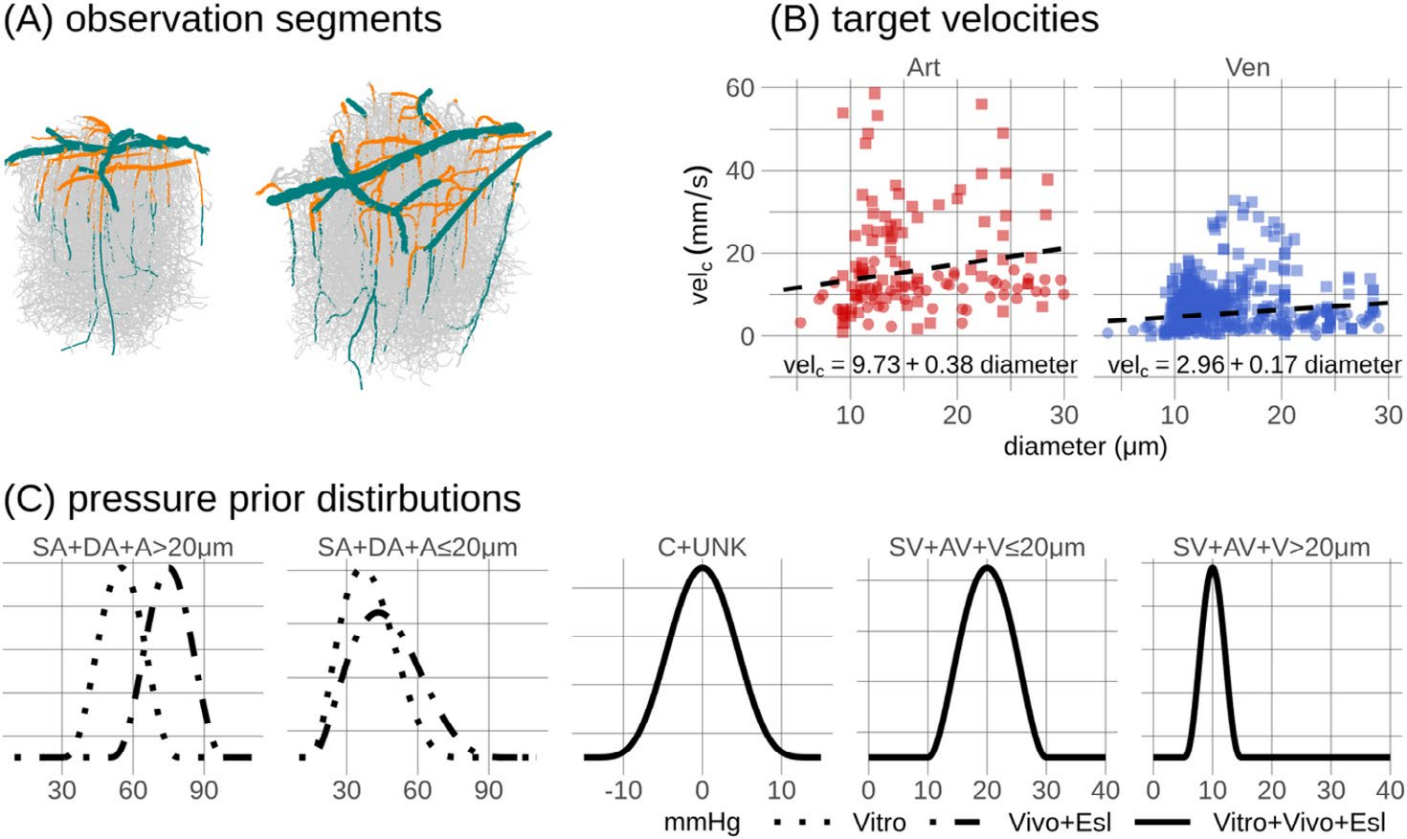
Target red blood cell velocities and flow directions in a subset of segments, and pressure prior distributions used in Bayesian calibration. In (A), spatial representations show vessel segments where target flow directions and target velocities were used in the Bayesian model calibration in Experiment 2. The union of green and orange segments highlights segments where target flow directions were used. Orange segments highlight segments where target velocities, dashed lines in (B), were used. Scatter points in (B) represent data underlying the linear fits, with details provided in Figure 2 in File S3. In (C), pressure prior distributions are shown. The same target velocities were applied across all calibration model variants. Consequently, priors with higher pressure levels in arterioles were adopted for the two models incorporating in vivo viscosity formulations (Vivo+Esl), to accommodate their increased hydraulic resistance [43, 44]. Priors governing vessel categories C and UNK are centered at 0, as these boundary conditions were defined as relative boundary conditions.

Bayesian calibration was informed by RBC velocities and flow directions in a subset of vessels. Since RBC velocities are not available for the two networks, diameter‐dependent linear fits to velocities derived from ref.Data and in vivo measurements in awake mice [61] were used to define target RBC velocities. Target velocities were used in vessel categories SA and DA + A (resp. SV and AV + V) collectively referred to as Art (resp. Ven) for vessels with diameters < 30 μm within AL1, matching the range of available in vivo velocity measurements [61]. Additionally, target blood flow directions in SAs, DAs, AVs, and SVs were determined by iterating through these vessels starting from presumed inflow and outflow end‐nodes of SAs and SVs, respectively. Segments assigned with target velocities and flow directions are highlighted in Figure 2A, and target velocities are shown in Figure 2B. Scaled and shifted beta distributions were used as prior distributions to describe uncertainties governing boundary pressures [27, 41], Figure 2C. Mathematical details regarding likelihood functions and prior distributions are available in Sections 6.1 and 6.2 in File S1.

The DREAM(ZS) algorithm [27, 62, 63, 64] was used for parameter inference, with algorithmic settings detailed in Section 6.3 in File S1. Five Markov chain Monte Carlo (MCMC) chains were run in parallel, with each chain undergoing 2 million iterations, resulting in a total of 10 million MCMC iterations. The DREAM(ZS) algorithm produced an archive of 100 000 samples, representing a thinned history of the MCMC chain samples. The convergence of the sampled chains to a limiting distribution was assessed using the Gelman Rubin statistics with a threshold of 1.2 to declare convergence [27, 54, 64, 65]. A subset of 1000 samples was retained for further analysis. The retained samples of pressure boundary conditions, one set of 1000 samples for each of the three calibration models per network, were then used with the respective flow simulation models that now incorporated the phase separation effect. This resulted in 1000 simulations per model per network, each reflecting diverse boundary pressure configurations and variable segment hematocrit levels for the cal.Vitro.ABC, cal.Vivo.ABC, and cal.Esl.ABC models.

Hemodynamic metrics, for each segment, were summarized by averaging across the 1000 model simulations. Similarly, uncertainty was quantified by the direction agreement rate calculated by the frequency at which the flow direction matched the predominant flow direction observed across the simulations.

## 3 Results

### 3.1 Experiment 1—Forward Modeling

#### 3.1.1 Validation of Reference Simulations Against Reference Data

**FIGURE 3:**
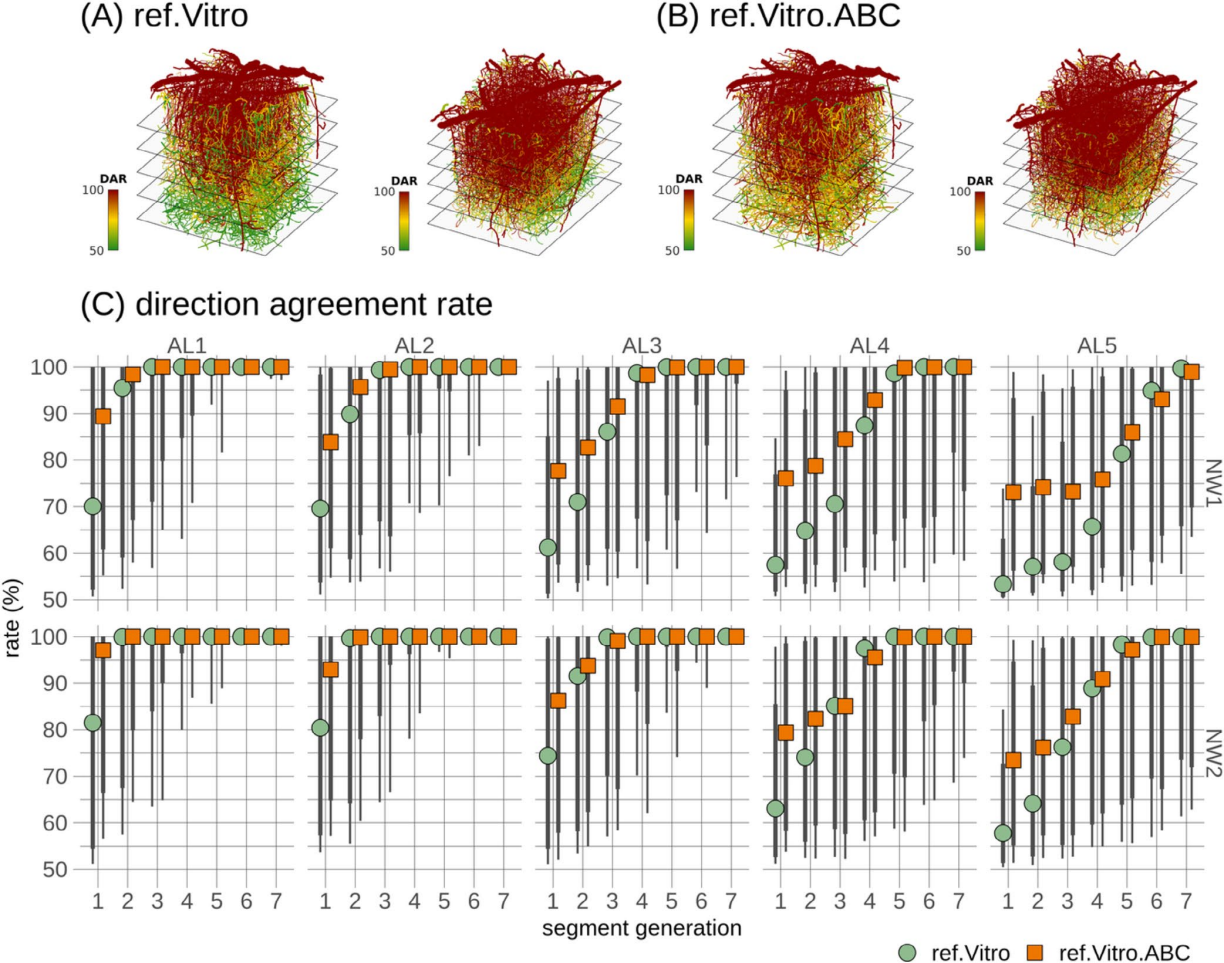
Quantitative analysis of blood flow direction uncertainty in the two reference models, ref.Vitro and ref.Vitro.ABC. In (A) and (B), each segment in the spatial representations of direction agreement rates (DAR) is uniquely colored according to its rate. A direction agreement rate of 100% corresponds to full agreement in blood flow direction, while a rate of 50% corresponds to complete disagreement. These rates are shown in the two models for both microvascular networks: one model with reference boundary pressures for all boundary nodes (ref.Vitro), and the other with adaptive boundary pressures for categories C and UNK boundary nodes (ref.Vitro.ABC). In (C), a summary of the direction agreement rates, grouped by network (NW1 and NW2), analysis layer (AL), and segment generation, is provided. Points denote medians across segments, while the thick and thin vertical lines represent [12.5 87.5] and [5 95] percentiles, respectively.

Initially, the ref.Vitro model simulations were validated against the reference data, ref.Data, through segment‐wise comparisons, and by histograms and summary statistics of the following key hemodynamic variables [35]: pressure, blood flow rate, shear stress, and blood flow velocity. Results, provided in Figure 3 in File S3 and Tables 3–8 in File S2, demonstrate that the ref.Vitro model simulations align closely with the hemodynamic metrics in ref.Data and thus provide a valid reference for subsequent analyses in the present study.

#### 3.1.2 Uncertainty Quantification

**FIGURE 4:**
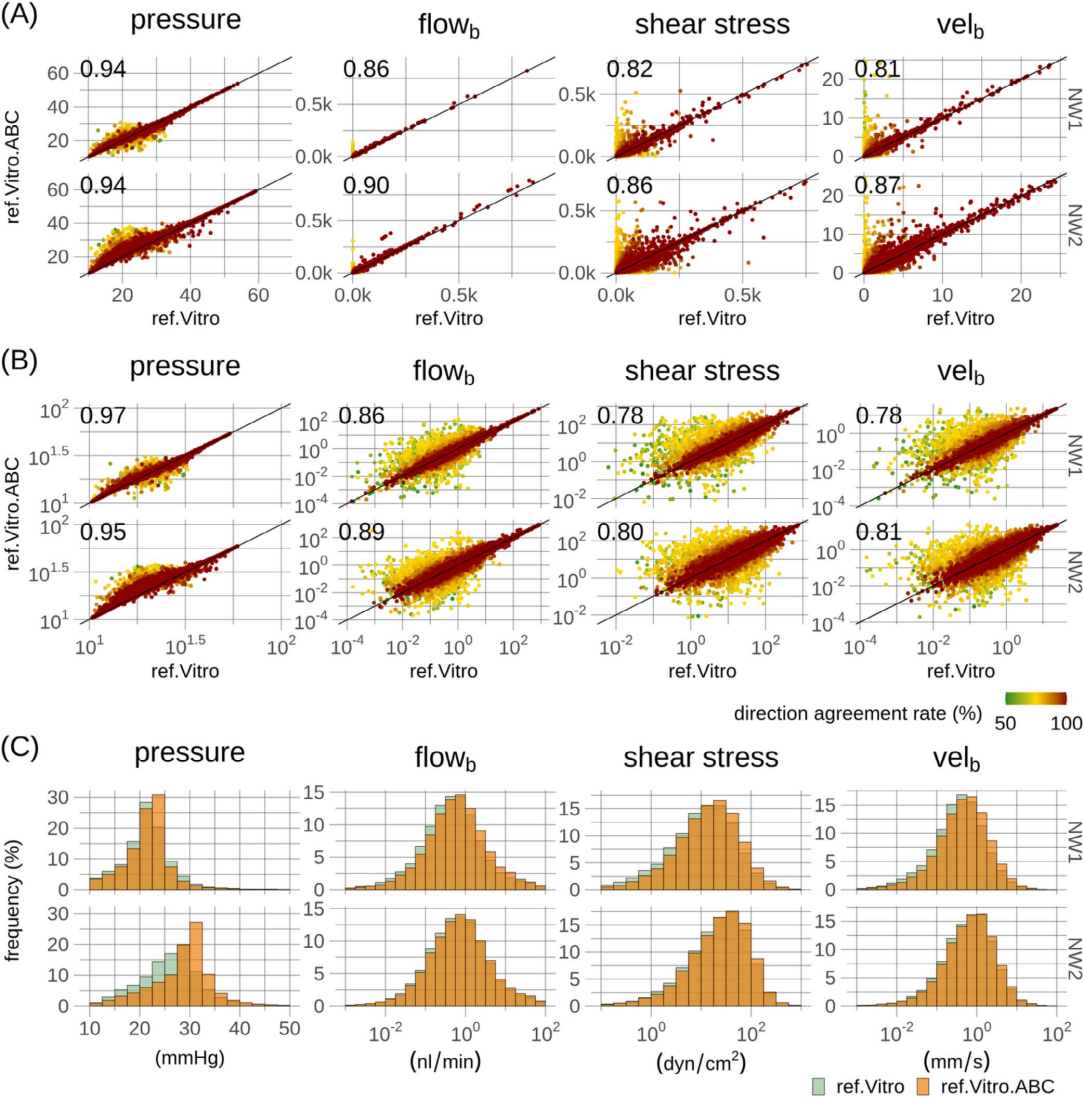
Quantitative comparison of hemodynamic metrics in the two reference models, ref.Vitro and ref.Vitro.ABC. In (A) and (B), each point in the scatter plots represents a vessel segment and is uniquely colored according to its direction agreement rate (DAR) averaged across the two models. For better visualization, scatter points are overlaid according to increasing DAR. The scatter plots are shown on a log‐scale in (B). Black lines represent identity lines, and correlation coefficients, Spearman's for (A) and Pearson for (B), are provided as inserts. Physical units of axes in (A) and (B) are consistent with the axis units of frequency histograms depicted in (C). Rows in (A–C) correspond to the two microvascular networks (NW1 and NW2).

After validating the ref.Vitro model simulations against ref.Data, the impact of pressure boundary condition uncertainty on blood flow uncertainty was evaluated in the ref.Vitro and ref.Vitro.ABC models. Direction agreement rates ranged from 50% to 100%, displaying considerable spatial heterogeneity, Figure 3A,B. This spatial heterogeneity was further quantitatively analyzed by grouping segments according to their respective AL and segment generation, Figure 3C. Decreased uncertainty with increasing segment generation was observed across all ALs. Additionally, a depth‐dependent influence was observed, with only the first few segment generations being markedly affected by blood flow direction uncertainty in AL1, while uncertainty escalated with depth in higher generation levels. The ref.Vitro model exhibited lower median values with increasing depth, but these differences should be considered in light of the extensive distributions represented by the vertical lines in Figure 3C and further detailed in Figure 4 in File S3. Consequently, a consistent pattern of spatial heterogeneity in blood flow uncertainty was observed in the two reference models across the two microvascular networks.

#### 3.1.3 Hemodynamic Predictions

Segment‐wise comparisons of the ref.Vitro and ref.Vitro.ABC models' hemodynamic predictions are shown in Figure 4A,B. Strong correlations were observed for non‐transformed variables (0.81–0.94, Spearman's), as well as for log‐transformed variables (0.78–0.97, Pearson). Segments governed by the most uncertainty exhibited the strongest scatter between the two models.

**FIGURE 5:**
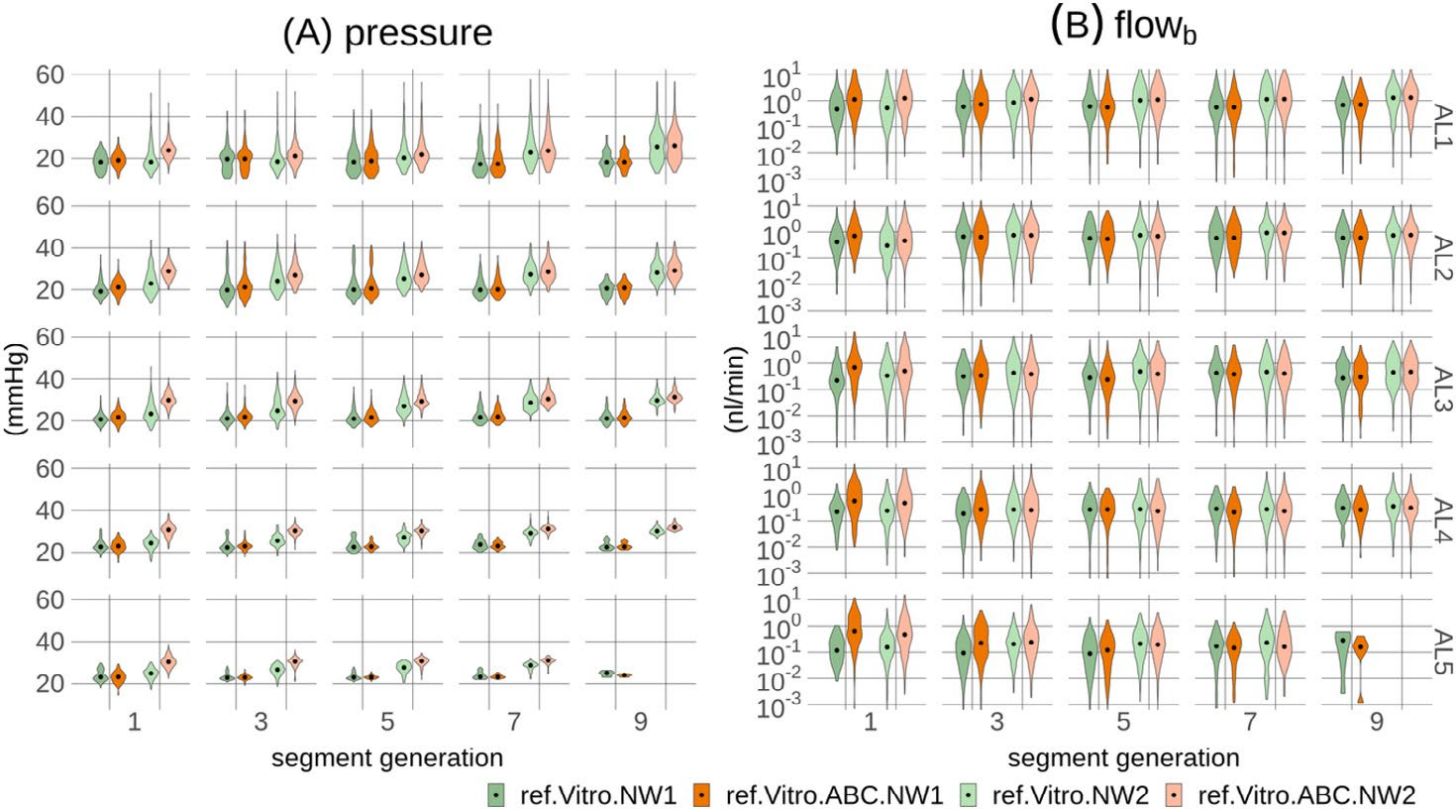
Depth‐wise and generation‐wise pressure profiles and blood flow profiles for capillary segments in the two reference models, ref.Vitro and ref.Vitro.ABC. In (A) and (B), different combinations of the two models (ref.Vitro and ref.Vitro.ABC) and the two microvascular networks (NW1 and NW2) are represented by unique colors. The plots are resolved by analysis layer (AL) in rows and segment generation in columns (see Figure 1 definitions of ALs and segment generation). Violins represent distributions over segments, and dots mark medians.

Frequency distributions of the hemodynamic variables, Figure 4C, and summary statistics, Tables 3–8 in File S2, demonstrate a notable congruence between the two models. The ref.Vitro.ABC model effectively captured the variations in hemodynamic variables across the two networks as observed for the ref.Vitro model. Figure 5 reveals consistent pressure and flow distribution patterns within segment subgroups, with differences in median pressures and blood flows mainly occurring in the lower segment generations. NW2 exhibited elevated pressures compared to NW1 in higher generations in both models. The ref.Vitro model showed a decline in pressure at lower generations, aligning with NW1 levels, whereas the ref.Vitro.ABC model maintained relatively uniform pressure across generations. Boundary conditions for the ref.Vitro model were derived from simulations within a larger synthetic network, leading to anticipated similar pressure levels across networks near their boundaries. In contrast, the ref.Vitro.ABC models used pressure boundary conditions defined relative to interior reference pressure, resulting in more uniform pressure profiles. Variations between the two models were confined to combinations of ALs and segment generations governed by must uncertainty, Figure 3.

Taken together, these results demonstrate good agreement in hemodynamic predictions between the two models and further suggest that uncertainty analysis offers a quantitative means for identifying a set of segments that exhibit a strong correlation between the hemodynamic variables in the two models.

### 3.2 Experiment 2—Inverse Modeling

#### 3.2.1 Calibration Fits

The Gelman Rubin $R_{c}$ statistics confirmed convergence of the MCMC sampled chains to a limiting distribution. Sets of samples of inferred pressure boundary conditions were subsequently used in models incorporating the phase separation effect. A quantitative assessment of the iterative procedure for hematocrit estimation demonstrated good convergence efficacy across all three model variants in both networks when exposed to the pressure boundary conditions inferred by our Bayesian calibration framework, Section 4 in File S1.

**FIGURE 6:**
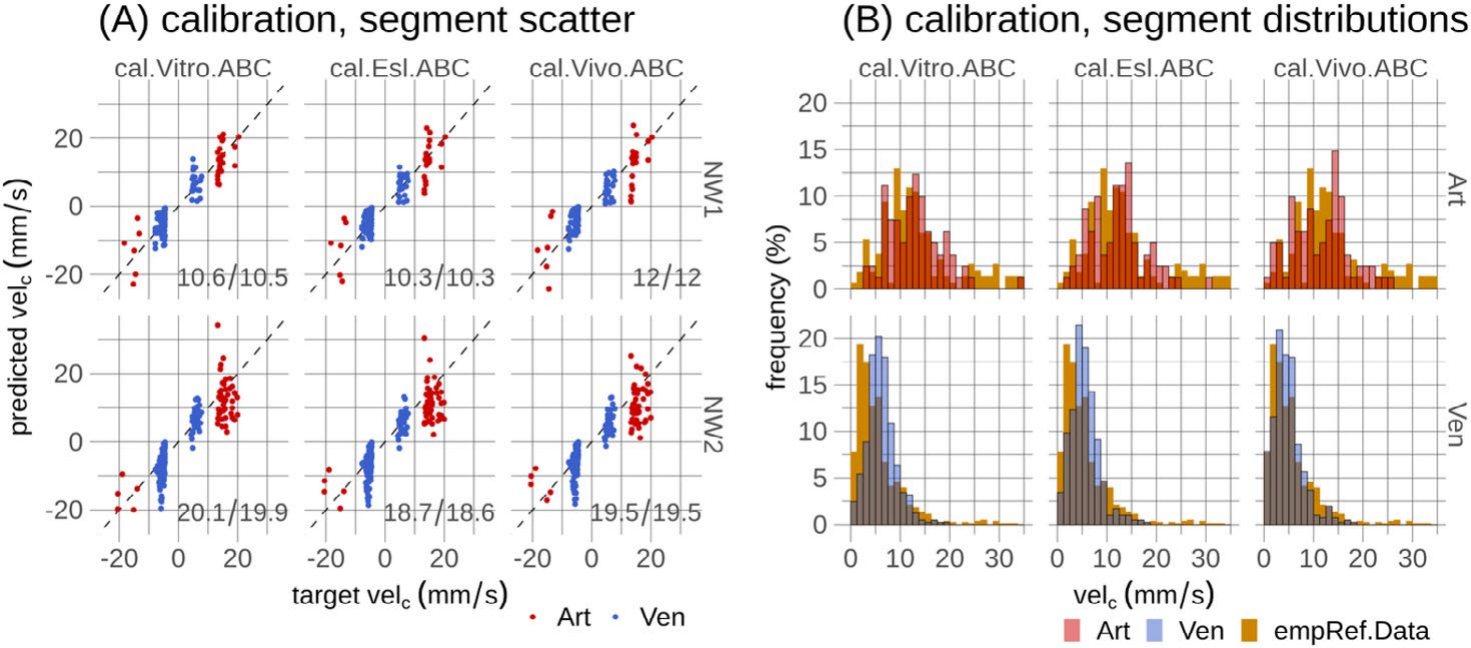
Predictions of the three calibration models, cal.Vitro.ABC, cal.Esl.ABC, and cal.Vivo.ABC. In (A), scatter plots comparing predicted red blood cell velocities with target velocities are shown. Points represent individual segments, dashed lines represent identity lines, and mean squared errors are shown as inserts. The three calibration models are shown in columns, and the two microvascular networks (NW1 and NW2) in rows. Segments where target velocities were used in model calibration are shown in Figure 2A. In (B), frequency histograms of predicted velocities in (A) are shown with vessel types in rows. These histograms are compared to the literature and reference data, empRef.Data, used to create diameter‐dependent target velocities. See Section 2.6.2 and Figure 2 in File S3 for further information about the target velocities.

Segment‐wise comparisons of target velocities against predicted velocities showed similar performance across the three calibration models, Figure 6A. Scatter points tend to form clusters along the horizontal axis (target velocity) since vessels governed by target velocities have similar diameters, Figure 2A. Points within these clusters exhibit scatter along the vertical axis (predicted velocity), resulting from the diameter‐dependent target velocities being adopted and agreeing well with the scatter observed for a given diameter in the data underlying the target values, Figure 2B. High congruence between frequency distributions governing the predicted velocities and the data underlying target velocities is observed for all three models, and all models captured well the distributional differences between Art and Ven observed in the underlying data, Figure 6B.

#### 3.2.2 Uncertainty Quantification

**FIGURE 7:**
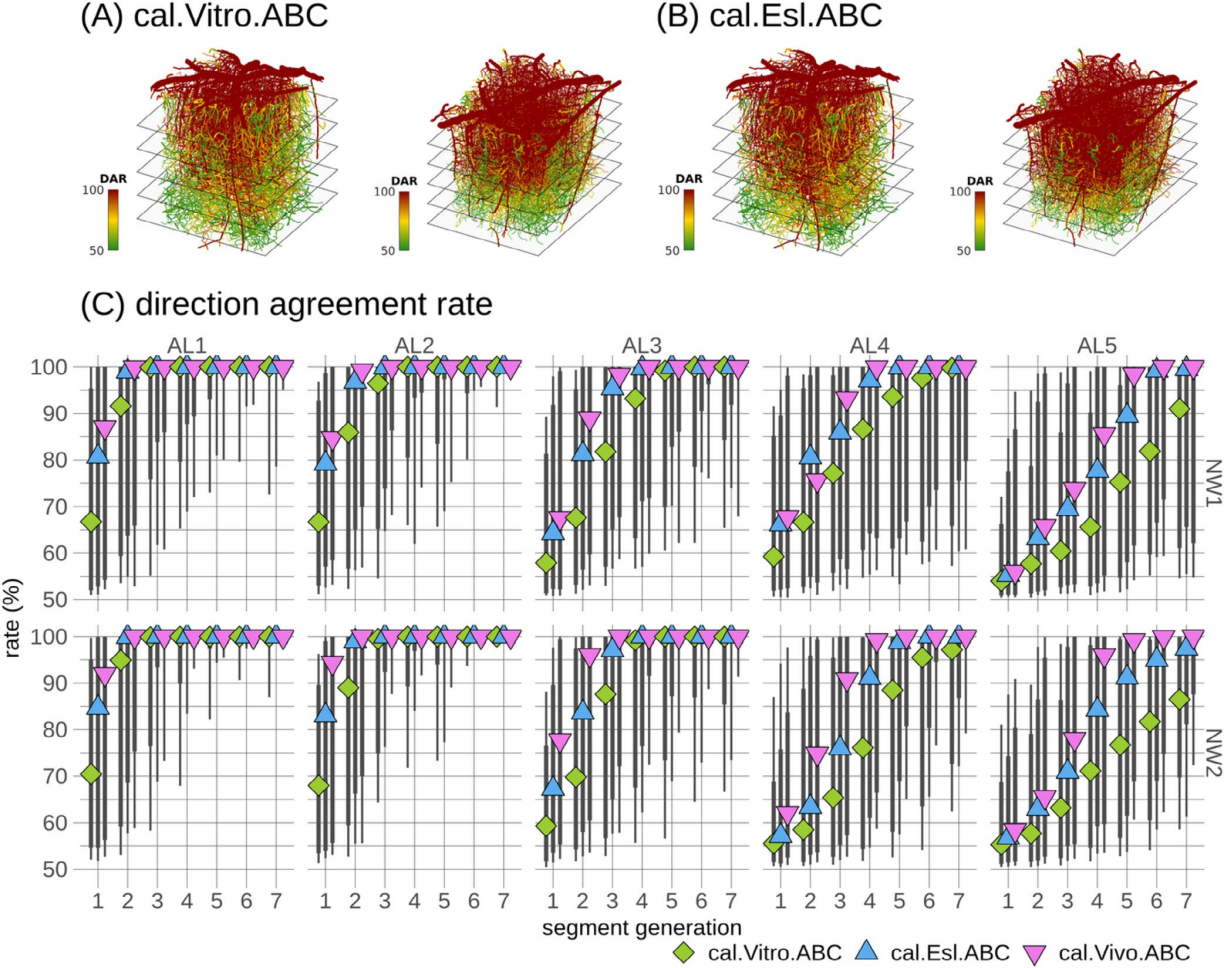
Quantitative analysis of blood flow direction uncertainty in the three calibration models, cal.Vitro.ABC, cal.Esl.ABC, and cal.Vivo.ABC. Same format as in Figure 3 but showing the cal.Vitro.ABC and cal.Esl.ABC models in (A) and (B) and all three calibration models in (C). The spatial representation of direction agreement rate (DAR) for the cal.Vivo.ABC model is similar to (B) and is available in Figure 5C in File S3.

Substantial heterogeneity in segment‐wise uncertainty was observed for all three calibration models, Figure 7 and Figure 5 in File S3, consistent with reference models, Figure 3. Medians governing depth and generation subgroups in the cal.Vivo.ABC and cal.Esl.ABC models followed each other and exhibited escalating uncertainty with depth, Figure 7C, in agreement with reference models, Figure 3C. The cal.Vitro.ABC model exhibited a similar pattern but with slightly increased uncertainty for deeper ALs. When interpreting these differences, the extensive distributions, indicated by vertical lines in Figure 7C, and further detailed in Figure 6 in File S3, should be considered.

#### 3.2.3 Hemodynamic Predictions

**FIGURE 8:**
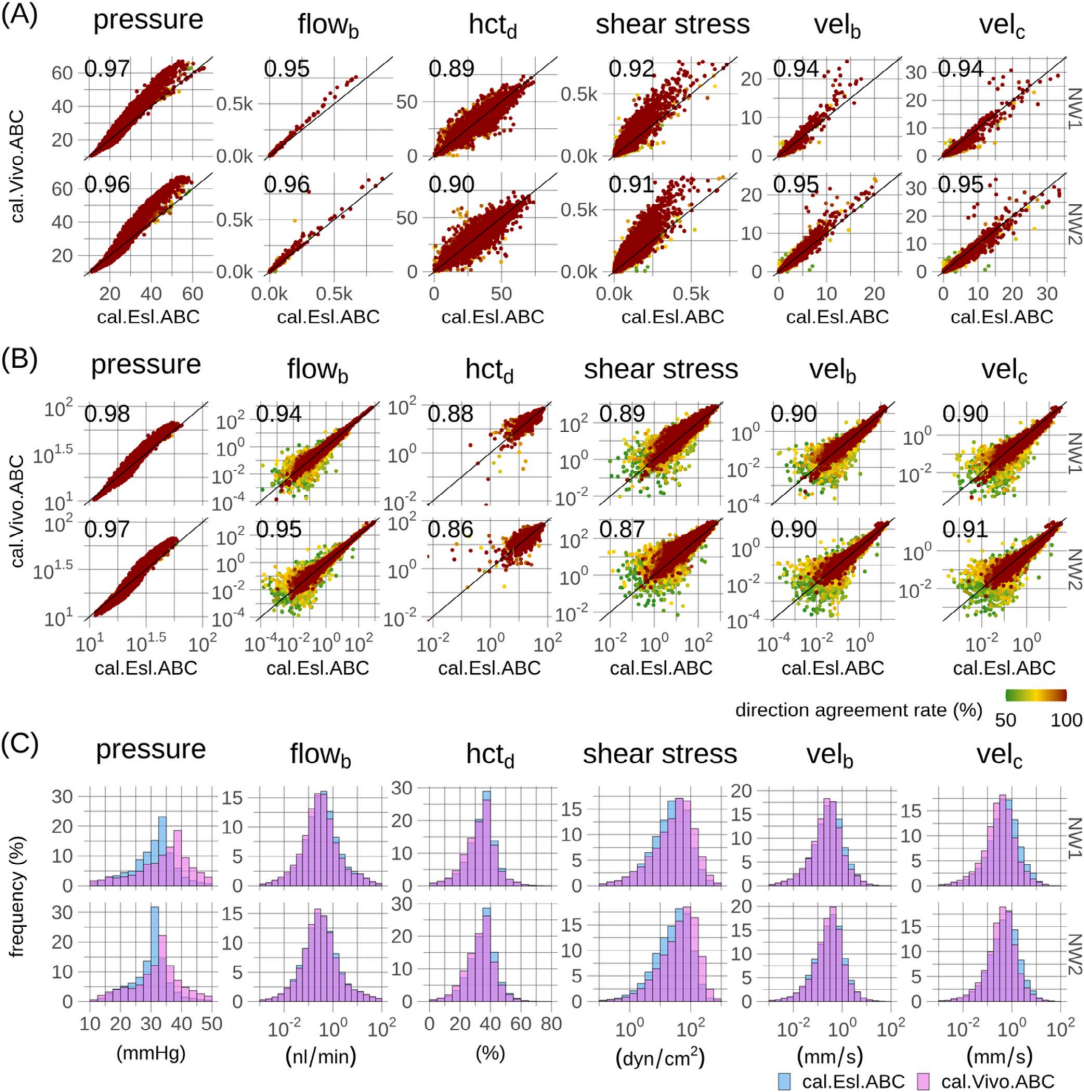
Quantitative comparison of hemodynamic metrics in two calibration models, cal.Vivo.ABC and cal.Esl.ABC. Same format as Figure 4. Hematocrits and red blood cell velocities are additionally compared, since these were considered as functional variables in both models.

Segment‐wise comparisons between the in vivo calibration models, cal.Vivo.ABC and cal.Esl.ABC, along with frequency histograms, are shown in Figure 8A–C and summarized in Tables 3–8 in File S2. Strong correlations were observed for both non‐transformed variables (0.89–0.97, Spearman's) and log‐transformed variables (0.86–0.98, Pearson), with most scatter among segments governed by most uncertainty, in agreement with the result in the reference models, Figure 4. Frequency histograms and summary statistics demonstrate high congruence between networks and models, with slightly increased pressures in the cal.Vivo.ABC model, reflecting its increased hydraulic resistance [43].

**FIGURE 9:**
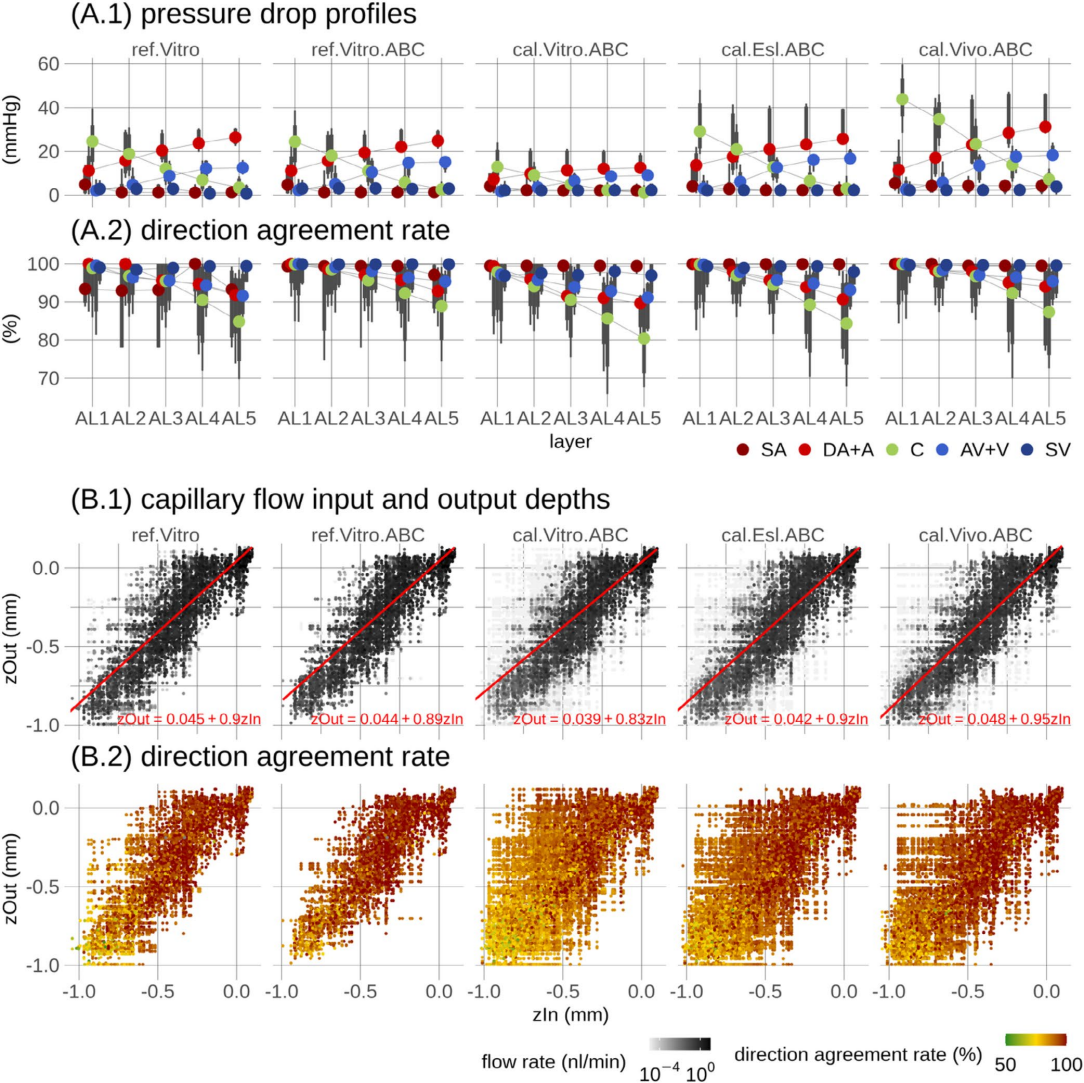
Pressure drop profiles and capillary input and output depths along pathways of blood flow. (A.1) Pressure drop profiles for each vessel category are shown. The paths of blood flow, which were tracked from boundary nodes of surface arterioles to surface venules, are grouped by analysis layers (ALs) based on the depth of the first capillary segment along the paths. The columns display the two reference models and the three calibration models. Points denote medians across paths, while the thick and thin vertical lines represent [12.5 87.5] and [5 95] percentiles, respectively. (A.2) shows a corresponding uncertainty analysis, with uncertainty quantified by the average direction agreement rate of segments across respective parts of individual flow paths. In (B.1), the depths of capillary inputs (zIn) and outputs (zOut) are presented. Individual paths are represented by points, with the intensity of the greyscale color indicating the path blood flow rate. For better visualization, colors are displayed on a log scale, and points are ordered according to intensity. Inserts provide linear fits and their corresponding equations. (B.2) shows direction agreement rates corresponding to points in (B.1) using the same point ordering. The summary statistics in (A.1 and A.2) and the fits in (B.1) were computed by weighting individual paths according to their blood flow rate. Figure 10 in File S3 provides complementary results based on RBC flow weighting in the three calibration models.

Comparisons between the in vitro reference and calibration models, ref.Vitro.ABC and cal.Vitro.ABC models, established a link between Experiments 1 and 2, Figure 7 in File S3. Despite increased scatter, good congruence was observed across hemodynamic variables, Tables 3–8 in File S2. Comparison between in vitro and in vivo calibration models further demonstrated good agreement and preservation of pressure hierarchy, despite lower pressures in the in vitro model, consistent with its decreased hydraulic resistance, Figures 8 and 9 in File S3.

### 3.3 Path‐Based Analysis of Pressure Drop Profiles and Capillary Flow Patterns Across Different Viscosity Models and Pressure Boundary Conditions

Depth‐dependent pressure drop profiles and layer‐wise capillary RBC flow patterns [32] were analyzed to further evaluate the proposed adaptive method for pressure boundary conditions and to examine whether these hemodynamic phenomena generalize across viscosity models including in vivo formulations. Flow paths between SA and SV boundary nodes were tracked for all five models. While reference models provided single simulations per network and hence single sets of paths, the calibration models each provided 1000 simulations per network, and resulting sets of paths were therefore aggregated across simulations for each model. Paths following the sequence SA → DA + A → C → AV + V → SV were retained, with up to two subsequent deviating transitions permitted and vessels with category UNK neglected to render the path selection criteria more robust to vessel labeling errors [32].

Layer‐wise pressure drop profiles, Figure 9A.1, showed depth‐dependency consistent with previously reported results [32]. The two reference models showed strong quantitative consistency and align closely with previously reported results [32]. Near the pial surface, the largest pressure drop occurs across vessels categorized as capillaries, whereas the pressure drop across arterioles becomes the dominant contributor with depth. The cal.Vitro.ABC model showed lower magnitude pressure profiles, in agreement with its more compact pressure distribution, Figure 7 in File S3 and Tables 3–8 in File S2. The in vivo models, cal.Esl.ABC and cal.Vivo.ABC, showed larger pressure drops consistent with their higher pressure levels and broader pressure distributions, Figure 8 and Figures 8 and 9 in File S3. Uncertainty analysis, Figure 9A.2, revealed that high blood flow stability was maintained along the flow paths underlying the pressure drop profiles, Figure 9A.1, with slight increases in uncertainty with increasing depth in agreement with Figures 3 and 7.

Layer‐wise capillary flow patterns showed that the correlations between cortical depths of capillary path endpoints [32] generalize to in vivo viscosity formulations, Figure 9B.1. Uncertainty analysis further supports the reliability of these depth‐wise correlations, Figure 9B.2.

## 4 Discussion

### 4.1 Boundary Conditions

#### 4.1.1 Proposed Adaptive Method for Pressure Boundary Conditions

This work proposes an adaptive method for pressure boundary conditions, which, to the best of the author's knowledge, has not been previously considered. The adaptive method ensures that statistical properties of boundary nodes resemble those of interior reference nodes while maintaining flexibility in relative pressure deviations, Section 2.3.1. The method inherently adapts its reference pressure levels and thereby eliminates the need for declaration or iterative estimation of these levels when exposed to various microvascular networks. The method is simple to implement, as it can be directly incorporated into the system of equations governing the blood flow model, Section 2.3.3.

Guided by previous research demonstrating decreased pressure with cortical depth [30, 32], interior reference nodes were partitioned into sets according to their cortical depth. The method thereby preserves sparsity, Section 2.3.3, maintains low computational demand, and thereby ensures scalability for large microvascular networks. The validity of utilizing groups of interior reference nodes is supported by the good agreement with simulations based on boundary pressures from the reference data set in hemodynamic variables (Figure 4 and Tables 3–8 in File S2), uncertainty profiles (Figure 3), and depth‐dependent pressure profiles and layer‐wise capillary blood flow profiles (Figure 9).

In Experiment 1, representing a typical in silico or forward modeling scenario, reference pressures were imposed at arterioles and venules. The adaptive method was applied to vessel categories of C and UNK, and heterogeneity across the relative boundary conditions was incorporated by sampling these from random distributions. The decision to use pressures from ref.Data for arterioles and venules, and the adaptive method for vessel categories C and UNK, mirrors a typical analysis scenario in which reference data for arterioles and venules would be available from the literature [31, 32, 34], albeit with some degree of uncertainty. Experiment 2, on the other hand, represents a typical data assimilation scenario where the three calibration models were combined with literature‐derived RBC velocities and presumed flow directions in a subset of vessels to facilitate inference over the unknown pressure boundary conditions, including both conventional and relative boundary conditions. To establish a link between Experiments 1 and 2, the adaptive method was also applied to vessel categories of C and UNK in Experiment 2, whereas the remaining boundary pressures were considered as conventional uncertain boundary pressures. It would, however, be straightforward to apply the adaptive method also to cut arterioles and venules in Experiment 2. In this case, additional depth‐dependent groups of reference nodes could be defined for the respective vessel categories, and relative pressure boundary conditions could then be defined relative to these groups while simultaneously being considered as uncertain variables.

#### 4.1.2 Conceptual Comparison With Other Approaches

The adaptive method follows a similar spirit as previous methods prescribing an adjusted but constant pressure at capillary boundary nodes [31] and prescribing pressures in cut arterioles and venules as equal to the mean pressure of the respective vessel classes of equal diameter at the same depth [37]. An important distinguishing feature of the adaptive method in this context is the application of the relative pressure deviations. Maintaining boundary conditions as free parameters in terms of relative pressure deviations is a desirable and unique feature of the proposed adaptive method as it acknowledges the inherent uncertainty of these boundary conditions, and it further facilitates the method's incorporation into data assimilation contexts as done in Experiment 2. For example, if information about blood flow directions in deeper cut arterioles and venules is incorporated into a data assimilation analysis, the added model flexibility, induced by the relative pressure deviations, will allow the inferred boundary conditions to adapt to maintain agreement between the simulated and observed blood flow directions [27].

Recent techniques have further focused on modeling heterogeneous, depth‐dependent boundary conditions by implanting microvascular networks into a larger artificial network designed to mimic the structural properties of the actual network [32], or by eliminating capillary boundary nodes by connecting segments located at opposite network faces [37]. Instead of eliminating boundary nodes and their corresponding boundary conditions, the proposed method maintains these variables but re‐references them relative to interior nodes, ensuring self‐consistency in node pressures while not allowing blood trajectories to re‐enter domain boundaries, reflecting the unknown history of blood entering the domain [32].

While the proposed adaptive method eliminates the need for designing large synthetic embedding networks, it introduces the additional requirements of defining interior reference nodes, defining weighting coefficients, and defining relative pressure deviations. A reasonable strategy is to define reference nodes based on some similarity measures, such as node type, morphology, or topology. Similarly, defining weighting coefficients to yield the average reference node pressure, as done in this study, is a reasonable and pragmatic first strategy.

Unlike connecting methods [37], the proposed adaptive method does not inherently enforce the assumption of balanced net inflow and outflow accumulated across domain boundaries. This characteristic is shared with other approaches such as embedding methods [32] and optimization‐based techniques [27, 36]. However, the flexibility of the adaptive method allows flow balancing to be considered if the investigator deems this additional constraint desirable for a given network architecture under study. For example, expectations about approximate flow balancing could be incorporated into the analysis by adjusting the relative pressure deviations to systematically diverge from the average reference node pressure, either globally or in spatially localized regions, to bias boundary pressures towards arteriolar or venular values, thereby supporting global flow constraints, as illustrated in Figure 12 in File S3. A similar effect could be achieved by employing nonuniform weighting coefficients in the adaptive method to emphasize arteriolar or venular reference node pressures. In the context of Bayesian calibration, the flow balance constraint can formally be incorporated into the likelihood function, and the flexibility of the adaptive method allows the pressure deviations to adjust accordingly. A systematic assessment of flow balancing as a method evaluation metric, along with an evaluation of the expected or tolerable magnitude of deviations from global flow conservation in the studied networks, is an interesting research direction but outside the scope of the present study.

### 4.2 Hemodynamic Simulations and Method Validation

To connect this study's findings with previous research, a methodical approach was adopted, gradually increasing model complexity. Initially, two reference models were considered with consistent hematocrit and hence hydraulic conductance, allowing exclusive evaluation of the proposed adaptive method for pressure boundary conditions against simulations based on reference pressure boundary conditions. Prescribed boundary conditions in the ref.Vitro model are based on simulations adopting network embedding to prescribe boundary conditions [32]. Hence, the comparison between the two reference models could be seen as a surrogate for a quantitative comparison of the adaptive method against boundary conditions based on an established embedding method [32], with the two reference models differing only in boundary conditions. The analysis, Figure 4 and Tables 3–8 in File S2, demonstrated good agreement between the two models, supporting the adaptive method's suitability for extensive microvascular networks. Although segment‐wise comparisons showed scatter, mostly confined to segments governed by most blood flow uncertainty, Figure 4A,B, high congruence between histograms, Figure 4C, and summary statistics, Tables 3–8 in File S2, was observed.

Once the adaptive approach's usefulness was established, it was incorporated into our Bayesian calibration framework for more complex analysis. Distributions over pressures at all boundary nodes were inferred by calibrating simulation models against diameter‐dependent target RBC velocities and presumed flow directions in a subset of arterioles and venules due to the lack of actual measurements. The aim was thereby to calibrate the models against characteristic data from the literature and reference data set, rather than matching segment‐wise hemodynamics of the reference model simulations. Despite this, consistencies between the reference and calibration models in hemodynamic estimates, depth‐dependent pressure drop profiles, and layer‐wise capillary flow phenomena were observed, Figure 9.

We previously incorporated a nonlinear blood flow simulation model, including the phase separation effect, into the Bayesian calibration stage in the analysis of mesenteric networks with extensive measurements, including hematocrit measurements [27]. However, inlet hematocrit only exhibited widespread systematic deviations from reference levels (prior means) when hematocrit measurements were included in model calibration. Since the currently studied networks lack hematocrit measurements, and since the study focused on pressure boundary conditions and their impact on blood flow uncertainty, the increased computational effort required for incorporating the nonlinear model into the Bayesian calibration was judged to yield marginal gains given the scope of the study. Subsequently utilizing sets of samples of inferred pressure boundary conditions in nonlinear simulation models, incorporating a well‐established phase separation model [25, 29, 30, 31, 37, 41, 43, 51, 66, 67], resulted in considerable heterogeneity in hematocrit throughout the microvascular networks (Figure 8 and Figures 8 and 9 in File S3), in agreement with previously reported results in cortical networks [27, 30, 31]. It is noted that the two‐step approach of first estimating pressure boundary conditions and subsequently adopting nonlinear simulations is in line with previous research [32].

Three viscosity model variants were considered in evaluating the proposed adaptive method in combination with our Bayesian calibration framework (cal.Vitro.ABC, cal.Esl.ABC, and cal.Vivo.ABC models). This facilitated systematic assessment across viscosity model variants currently applied to extensive microvascular networks [27, 30, 31, 32, 35, 37, 45, 56, 59]. Simulation models incorporating the three variants were calibrated against the same target velocities and flow directions. Predicted pressures were therefore expected to differ across the three models [43] which was confirmed by the analysis (Figure 8, Figures 8 and 9 in File S3, and Tables 3–8 in File S2). Shear stress, proportional to the pressure difference across a vessel segment, showed a similar effect. Despite systematic pressure differences, a strong correlation between segment pressure was observed across models (Figures 8 and 9 in File S3), preserving pressure hierarchy within the microvascular networks. The following two factors may contribute to this similarity: Blood flow resistance is proportional to the product of segment length and effective viscosity divided by the fourth power of segment diameter. Segment diameter thereby has a strong influence on resistance independent of viscosity [43]. Additionally, microvascular networks have a highly interconnected architecture which, together with the strong heterogeneity in segment morphology, shapes the heterogeneities of hemodynamic variables in the microcirculation [3, 4]. Formal statistical model selection between the three viscosity models is outside the study's scope, challenged by the lack of hemodynamic measurements in the examined networks. A comprehensive discussion of viscosity and flow resistance in the microcirculation is available in the cited literature [1, 43, 44, 46].

Consistencies, across models in segment hemodynamic variables, in depth dependencies in pressure drop profiles, and in capillary flow patterns resulted from analyses comparing the proposed adaptive method for pressure boundary conditions against simulations based on reference pressure boundary conditions (Experiment 1) or identical target RBC velocities and target flow directions in the Bayesian calibration (Experiment 2). Scatter was noted in the segment‐wise comparisons between models. The variability between the reference models was expected due to their differences in boundary conditions and the known influence of boundary conditions on simulated hemodynamics. Similarly, scatter was also expected and observed between models incorporating different viscosity models.

In the absence of extensive hemodynamic measurements, a commonly used approach to model validation, including methods for boundary conditions, is to compare model simulations to in vivo measurements from literature at the level of summary statistics, that is, hemodynamic metrics aggregated across numerous vessels of typical categories. Following this approach, it is noted that the presented model simulations, as summarized in Tables 3–8 in File S2, agree well with reported ranges of in vivo RBC velocity measurements in arterioles [61, 68, 69, 70], venules [61, 68, 69], and capillaries [69, 70, 71, 72, 73, 74, 75, 76, 77, 78]. Consequently, evidence is provided that the proposed adaptive approach and its incorporation into our Bayesian calibration framework yield flow simulations that are consistent with an established method for establishing pressure boundary conditions and blood flow simulations [32], as well as with in vivo velocity measurements.

### 4.3 Uncertainty Quantification

A probabilistic approach to quantify the influence of uncertainties in imposed pressure boundary conditions in linear forward modeling was adopted, Section 2.4.2, and evaluated, Section 3.1.2. The advantage of this method's simplicity is availability of all required variables in current blood flow simulation models (matrix H, Equation 7). The only additional information needed is the strength of uncertainty governing individual boundary conditions, as the influence of this uncertainty on blood flow depends on its assumed strength. For example, Experiment 1 considered identical strength of uncertainty across relative pressure boundary conditions resulting in proportional standard deviation of blood flow to the boundary pressures' uncertainty. Despite its simplicity, the probabilistic approach to uncertainty analysis in forward models, Section 2.4.2, effectively quantified the impact of pressure boundary condition uncertainty on model predictions. Moreover, good agreement between layer‐wise blood flow uncertainty profiles was similarly observed between the reference and calibration models, Figures 3 and 7.

The spatial distributions of blood flow uncertainty, quantified by the direction agreement metric, for both reference models and calibration models exhibited substantial heterogeneity in segment‐wise uncertainty, influenced by depth and generation, Figures 3 and 7. Considerable heterogeneity was noted among vessels within a given depth and generation group. Although there is a consistent pattern of increasing uncertainty in deeper segment generations with cortical depth, future research in brain cortical microvasculature should not rely solely on the median statistics for individual segment generations in Figures 3 and 7 to guide vessel selection for analysis. This is because the blood flow field, and consequently the uncertainty estimate for individual vessels, is likely influenced by factors such as the imposed boundary conditions and the specific network topology. Future studies are instead recommended to conduct a quantitative uncertainty analysis, using formal approaches like those adopted here.

Although unknown boundary conditions can be imposed by interpolating literature‐derived data, numerical optimization techniques, or embedding or connecting techniques, they remain inherently uncertain. For example, literature‐based or observed data used for model fitting is likely to be corrupted by some amount of physiological variability and measurement error. Embedding and connecting techniques may also be sensitive to how the microvascular network is truncated, the spatial location of the network within the larger synthetic domains, or specific connectivity of vessels across domain boundaries. In such applications, uncertainty analysis can be useful in assessing the reliability of hemodynamic predictions and in guiding the objective selection of specific simulated variables for further analysis.

More advanced parameterizations representing boundary uncertainty, such as spatially varying uncertainty profiles, could be implemented. For example, the smooth nature of the pressure field in microvascular networks could be exploited by incorporating expectations about the field's correlation structure into the covariance matrix $\Sigma_{A}$ in the probabilistic approach used in forward models, Section 2.4.2, or by incorporating correlation structure into the prior distribution governing boundary pressures in Bayesian calibration, Section 2.4.3. Whereas such an approach could potentially reduce uncertainty, formally establishing and validating it is beyond this study's scope.

### 4.4 Limitations and Future Research

The primary limitation of this study is the lack of measured hemodynamic data in the examined microvascular networks. Instead, model simulations was compared to simulations of an established model [32] in Experiment 1, whereas linear fits to literature‐derived data from awake imaging in mice [61] and RBC velocities from the reference data set [32] were used to define diameter‐dependent target velocities in Experiment 2. Additionally, model simulations agreed well with reported ranges of in vivo RBC velocity measurements. While these analyses provide a reasonable basis for the quantitative evaluation of the proposed adaptive method for pressure boundary conditions and its integration into our Bayesian calibration framework, it is noted that hemodynamic variables derived from the reference data set represent simulations from an established model rather than ground truth data. Similarly, the additional literature data used to guide the Bayesian calibration represent characteristic velocities rather than exact measurements from vessels in the examined networks. The ongoing development of experimental techniques [79] enabling comprehensive measurements of hemodynamic variables in extensive microvascular networks will likely support the continued development and validation of the proposed methodology [27, 29, 41, 43].

Another limitation to the study is the uncertainty related to vessel diameters and vessel type categorization as previously discussed [32]. The rheological descriptions and the resulting hydraulic resistances strongly depend on vessel diameter, especially for small diameters, and hence significantly influence hemodynamic simulations [29, 30, 44, 46]. Vessel tortuosity was considered in calculating segment lengths, whereas uniform diameters along individual segments were used [32]. These capillary diameters have previously been adjusted to match empirical distributions while maintaining the hierarchy of vessel diameters [32]. Future data sets with extensive hemodynamic measurements, including those in smaller vessels, could further facilitate the incorporation of uncertainties in vessel diameters, for example, into the calibration analysis and the adaptation of these diameters to align model predictions with measurements [28].

Whereas this study focused on the influence of boundary pressure uncertainty on model simulations, future studies could expand to include other sources of uncertainties, such as those related to diameters, as discussed above, to the parameters of the rheological descriptions including the phase separation model [41], or uncertainties governing boundary hematocrits [27, 41], including the interaction between pressure and hematocrit uncertainties. Additionally, future analyses could also further investigate the effects of measurement uncertainty and consider more advanced statistical error models [27]. Again, future comprehensive measurements will support these research directions.

In future in silico studies examining, for example, vessel diameter changes during functional activation [45] or localized occlusions in stroke [59], the adaptive method can be applied using the established approach [45, 59] that estimates and maintains baseline boundary conditions across scenarios. Additionally, interior nodes close to perturbation sites can be excluded from the reference node sets. For studies with widespread perturbations, such as capillary occlusions in Alzheimer's disease [80], applying the adaptive method to perturbed networks ensures boundary conditions reflect interior conditions under perturbation. Uncertainty quantification can enable identification of network regions robust to boundary condition uncertainty, guiding selection of perturbation sites or areas for further analysis. In inverse modeling, integrating the adaptive method into assimilation techniques will facilitate network‐oriented analysis [1, 2, 27] of sparse hemodynamic measurements. When applied to various tissue types or regions, hemodynamic measurements from the respective tissue region could be incorporated, whereas literature data characteristic of the specific region could be used in the absence of actual measurements [27, 28].

In summary, our Bayesian calibration framework, with the proposed adaptive method for pressure boundary conditions incorporated into it, proved its validity and scalability for blood flow simulation and uncertainty quantification in high‐dimensional settings with thousands of segments and unknown boundary conditions. It is anticipated that the adaptive method for pressure boundary conditions will be useful in future applications of both forward and inverse blood flow modeling, and that uncertainty quantification will be valuable in complementing hemodynamic predictions with associated uncertainties.

## 5 Perspectives

An adaptive method for selecting appropriate pressure boundary conditions in microvascular blood flow simulation models was proposed and rigorously evaluated in extensive microvascular networks from the brain cortex. Probabilistic approaches were employed to quantitatively assess the influence of the inevitable boundary condition uncertainty on blood flow simulations, complementing hemodynamic predictions with associated uncertainties. This methodology holds promise for future simulation studies of microcirculatory phenomena, both in health and disease.

## Disclosure

Generative AI: This paper reports the author's own work and ideas. Microsoft Word and Microsoft Copilot were employed for spell‐checking, grammar‐checking, and suggesting rephrasing of smaller pieces of original text to improve readability. All corresponding text was subsequently reviewed and edited by the author. No original content was generated by these tools, and the author takes full responsibility for the content of the publication. This declaration is provided to maintain transparency and integrity in accordance with publisher's and institutional guidelines.

## Conflicts of Interest

The author declares no conflicts of interest.

## Supporting information


**File S1:** micc70027‐sup‐0001‐FileS1.pdf.

**File S2:** micc70027‐sup‐0002‐FileS2.pdf.

**File S3:** micc70027‐sup‐0003‐FileS3.pdf.

## Data Availability

The methods for blood flow simulation, uncertainty quantification, and Bayesian calibration described in this paper are implemented in C++ and are available in a publicly available open‐source software suite, NetInf, at GitHub https://github.com/neuro8000/NetInf. The repository contains source code, build instructions, vignettes for analyses, and example data. Data files with model simulation results, underlying the presented summary figures and tables, and analysis scripts for reproducing figures and tables from these data files are available at Zenodo https://doi.org/10.5281/zenodo.15373376.
