# Supplementary material for: Blood Flow Simulation and Uncertainty Quantification in Extensive Microvascular Networks: Application to Brain Cortical Networks
Source: Microcirculation. 2025 Sep 21;32(7):e70027. doi: 10.1111/micc.70027 (PMC12450458; doi:10.1111/micc.70027)
Supplement: Supplementary file 1 — File S1: micc70027‐sup‐0001‐FileS1.pdf. [file MICC-32-e70027-s001.pdf]

Blood flow simulation and uncertainty quantification in extensive microvascular networks: Application to brain cortical networks

File S1
Supplementary text

2025.08.19

Peter Mondrup Rasmussen[1*]
1 Center of Functionally Integrative Neuroscience, Department of Clinical Medicine, Aarhus University, Aarhus, Denmark. * Corresponding author: pmr@cfin.au.dk.

Contents

1 Blood flow modeling - governing equations **2**

2 Preserving sparsity in the adaptive method for pressure boundary conditions **2**

3 Empirical descriptions of blood's rheology **3**
3.1 Adjusting for differences in mean corpuscular volume . . . 3
3.2 Descriptions of apparent viscosity and the Fåhraeus effect . . . 3
3.2.1 In-vitro model . . . 3
3.2.2 In-vivo model . . . 5
3.2.3 In-vivo Esl model . . . 5
3.3 Description of the phase separation effect . . . 6

4 Hematocrit iterations **7**

5 Probabilistic uncertainty quantification in linear forward models - relationship with sensitivity analysis **8**

6 Bayesian calibration **8**
6.1 Likelihood functions and prior distributions governing their parameters . . . 8
6.2 Prior distributions governing boundary pressures . . . 9
6.3 DREAM(ZS) algorithmic settings . . . 11

7 References **12**

1 Blood flow modeling - governing equations

Let a microvacular network comprise m segments and n nodes. Segment blood flow rates $\mathbf{q} \in \mathbb{R}^{m\times 1}$ are modeled as linearly related to node pressures $\mathbf{p} \in \mathbb{R}^{n\times 1}$ by

$$\mathbf{q} = \mathbf{M}\mathbf{p} \tag{1}$$

where $\mathbf{M} \in \mathbb{R}^{m\times n}$ is a sparse matrix of segment hydraulic conductance (inverse resistance) with non-zero elements

$$M_{j,i} = \frac{\pi \delta_{j,i} d_j^4}{128 \lambda \mu_j l_j} \tag{2}$$

$\delta_{j,i}$ is $+1$ (resp. -1) if node i is start node (resp. end node) of segment j, while d_j, l_j, and μ_j denote segment diameter, length, and effective viscosity, respectively. λ is a constant accounting for conversion between physical units (see below). Positive flow direction is defined from segment start node to end node.

Blood flows at interior nodes sum to zero (mass conservation), which together with imposed pressure or flow boundary conditions encoded in $\mathbf{b} \in \mathbb{R}^{n\times 1}$ yields

$$\mathbf{K}\mathbf{p} = \mathbf{R}\mathbf{b} \tag{3}$$

In (3), $\mathbf{K} = \mathbf{L}\mathbf{M} + \mathbf{J} + \mathbf{N}$. $\mathbf{L} \in \mathbb{R}^{n\times m}$ is a sparse matrix with non-zero elements if node i is either an interior node or a boundary node with an imposed blood flow rate boundary condition; then $L_{i,j} = -1$ (resp. $L_{i,j} = 1$) if the node i is the start node (resp. end node) of segment j. $\mathbf{J} \in \mathbb{R}^{n\times n}$ is a sparse matrix with non-zero elements $J_{i,i} = 1$ if a pressure boundary conditions is imposed at boundary node i. $\mathbf{N} \in \mathbb{R}^{n\times n}$ is a zero matrix and $\mathbf{R} \in \mathbb{R}^{n\times n}$ is an identity matrix. Elements in $\mathbf{N}$ and $\mathbf{R}$ are modified in the adaptive method for pressure boundary conditions (see Section 2.3.3, main text).

Boundary conditions are encoded in the sparse vector $\mathbf{b}$ in (3), with non-zero elements $b_i = -|q_i|$ (resp. $b_i = |q_i|$) if blood inflow rate (resp. outflow rate) $|q_i|$ is imposed at boundary node i, or $b_i = p_i$ if boundary pressure p_i is imposed at boundary node i (or $b_i = \Delta p_i$ if a relative boundary pressure is imposed, see Section 2.3.3, main text).

Physical units used in (1) to (3) are as follows

quantity	q_j	p_i	d_j	l_j	μ_j
physical unit	$(\mu\text{m})^3/\text{s}$	mmHg	μm	μm	cP (centipoise)

and $\lambda = 7.5 \cdot 10^{-6}\ \frac{\text{mmHg}\cdot\text{s}}{\text{cP}}$, since 1 cP = 0.001 Pa $\cdot$ s and 1 Pa $\approx$ 0.0075 mmHg.

2 Preserving sparsity in the adaptive method for pressure boundary conditions

The adaptive method can be incorporated into the governing system of equations (1) and (3) as described in Section 2.3.3 in the main text. Sparsity may, however, be significantly reduced if numerous boundary nodes are subjected to relative pressure boundary conditions, and many reference nodes are utilized concurrently. This decrease in sparsity can subsequently result in a substantial increase in the computational demand required to solve the system of linear equations (3). However, it is often

reasonable to assume that multiple boundary nodes may have identical sets of reference nodes, as the pressures at these boundary nodes may still vary due to the application of the relative pressure deviation Δp_i at the level of individual boundary nodes. In one extreme scenario, all boundary nodes governed by relative pressure boundary conditions could all have identical sets of reference nodes, which could be defined by all interior nodes with equal weight, for example. In this case, the pressures at these boundary nodes would be defined relative to the average interior node pressure. Alternatively, sets of reference nodes could be identical for boundary nodes that possess a common structural or topological property.

Consider $\mathcal{G}_g$ as a set of boundary nodes that all have identical sets of reference nodes, with the weighting coefficients for individual reference nodes also being identical across these boundary nodes. Sparsity can then be increased by designating one of the boundary nodes $i \in \mathcal{G}_g$ as a primary node and the remaining boundary nodes $i' \in \mathcal{G}_g, i' \neq i$ as secondary nodes. This requires modifying the definition of non-zero elements in $\mathbf{N}$ and the corresponding elements in $\mathbf{R}$ in (3). For the specific group of boundary nodes $\mathcal{G}_g$, $\mathbf{N}$ will then contain non-zero elements $N_{i,s} = -w_{is}$ only for the primary node i. Rows corresponding to secondary nodes will, on the other hand, only have one non-zero elements at $N_{i',i} = -1$. The elements in $\mathbf{R}$ need to be adjusted accordingly and will, in addition to the ones along the diagonal, contain non-zero off-diagonal elements $R_{i',i} = -1$ for secondary nodes.

The enhanced sparsity of $\mathbf{N}$ has a crucial implication since it can lead to a tremendous reduction in the computational demand needed for solving (3). This reduced computational demand is particularly important when the system of equations must undergo multiple solutions, such as during hematocrit iterations or in calibration analysis.

3 Empirical descriptions of blood's rheology

In the equations that follow, coefficients are denoted by mathematical symbols to ensure uniformity throughout all equations. Numerical coefficient values, which all have been reported in prior studies, are provided in associated tables.

3.1 Adjusting for differences in mean corpuscular volume

A scaling factor γ was used to adjust for differences in mean corpuscular volume (MCV) between human (MCV_h) and other species (MCV_s) (here mice)

$$\gamma = \left(\frac{MCV_h}{MCV_s}\right)^{\frac{1}{3}} \tag{4}$$

with scaled diameter $\tilde{d}$ defined by

$$\tilde{d} = \gamma d, \tag{5}$$

where d is the measured diameter[1].

3.2 Descriptions of apparent viscosity and the Fåhraeus effect

3.2.1 In-vitro model

The following parametric functions, describing relative apparent viscosity, were used in the ref.Vitro, ref.Vitro.ABC, and cal.Vitro.ABC models. The ref.Vitro and ref.Vitro.ABC models utilized non-scaled diameters d instead of $\tilde{d}$ as explained in the main text.

The dependence of relative apparent viscosity μ_{rel} (relative to plasma viscosity) on diameter $\tilde{d}$ and discharge hematocrit h_d was described by[1, 2]

$$\mu_{rel} = 1 + (\mu_{0.45} - 1)\frac{(1-h_d)^C - 1}{(1-0.45)^C - 1} \tag{6}$$

where

$$\mu_{0.45} = x_0 e^{-x_1 \tilde{d}} + x_2 - x_3 e^{-x_4 \tilde{d}^{x_5}} \tag{7}$$

and

$$C = \left(y_0 + e^{-y_1 \tilde{d}}\right)\left(-1 + \frac{1}{1 + 10^{-y_2}\tilde{d}^{y_3}}\right) + \frac{1}{1 + 10^{-y_2}\tilde{d}^{y_3}} \tag{8}$$

Coefficients used in (7) and (8).

coefficient	x_0	x_1	x_2	x_3	x_4	x_5	y_0	y_1	y_2	y_3
value	220	1.3	3.2	2.44	0.06	0.645	0.8	0.075	11	12

The Fåhraeus effect was described by[1, 2]

$$\frac{h_t}{h_d} = \begin{cases} h_d + (1-h_d)\left(1 + z_0 e^{-z_1 \tilde{d}} - z_2 e^{-z_3 \tilde{d}}\right) & \text{if } \tilde{d} \geq \tau \\ 1 & \text{otherwise} \end{cases} \tag{9}$$

where h_t denote tube hematocrit. The threshold

$$\tau = \frac{log\left(\frac{z_2}{z_0}\right)}{z_3 - z_1} \tag{10}$$

prevents the empirical description to become greater than one in the limit of very for small diameters ($\sim 2.58\mu m$ for the standard coefficients) in combination with low hematocrit.

Coefficients used in (9) and (10).

coefficient	z_0	z_1	z_2	z_3
value	1.7	0.415	0.6	0.011

The description (9) was used to convert tube hematocrits available in the reference data set to discharge hematocrits to be used for calculating the effective viscosities used in the ref.Vitro and ref.Vitro.ABC models.

The cal.Vitro.ABC model incorporated hematocrit as a functional variable, and red blood cell (RBC) velocity v_c was calculated by

$$v_c = v_b \left(\frac{h_t}{h_d}\right)^{-1} \tag{11}$$

where

$$v_b = \frac{4q}{\pi d^2} \tag{12}$$

is the blood flow velocity and q is the blood flow rate. RBC velocities for ref.Data, as summarized in Tables 3 to 8 in File S2, were also computed by (11) based on average blood flow rates and discharge hematocrit (from tube hematocrits by (9)) available in the reference data set, ref.Data.

3.2.2 In-vivo model

The following parametric functions were used in the cal.Vivo.ABC model.

The relative apparent viscosity was described by[1, 2]

$$\mu_{rel} = \left(1 + (\mu_{0.45} - 1)\frac{(1-h_d)^C - 1}{(1-0.45)^C - 1}\left(\frac{\tilde{d}}{\tilde{d} - w_6}\right)^2\right)\left(\frac{\tilde{d}}{\tilde{d} - w_6}\right)^2 \tag{13}$$

where

$$\mu_{0.45} = w_0 e^{-w_1 \tilde{d}} + w_2 - w_3 e^{-w_4 \tilde{d}^{w_5}} \tag{14}$$

and C defined as in eqn (8).

Coefficients used in (13) and (14).

coefficient	w_0	w_1	w_2	w_3	w_4	w_5	w_6
value	6	0.085	3.2	2.44	0.06	0.645	1.1

The Fåhraeus effect and the velocities were calculated as in the in-vitro model (9), (11), and (12).

3.2.3 In-vivo Esl model

The following parametric functions were used in the cal.Esl.ABC model.

The relative apparent viscosity was described by[1, 3]

$$\mu_{rel} = \left(1 + (\mu_{0.45} - 1)\frac{(1-h_d)^C - 1}{(1-0.45)^C - 1}\right)\left(\frac{\tilde{d}}{\tilde{d}_{eff}}\right)^4 \tag{15}$$

with $\mu_{0.45}$ and C calculated as in the in-vitro model (7) and (8) but with $\tilde{d}$ substituted by $\tilde{d}_{ph}$.

The two diameters $\tilde{d}_{ph}$ and $\tilde{d}_{eff}$ were calculated by the following axillary functions

$$W_{as} = \begin{cases} 0 & \text{if } \tilde{d} \leq \theta_0 \\ \theta_7 \frac{\tilde{d} - \theta_0}{\tilde{d} + \theta_2 - 2\theta_0} & \text{if } \tilde{d} > \theta_0 \end{cases} \tag{16}$$

$$W_{peak} = \begin{cases} 0 & \text{if } \tilde{d} \leq \theta_0 \\ \theta_3 \frac{\tilde{d} - \theta_0}{\theta_1 - \theta_0} & \text{if } \theta_0 < \tilde{d} \leq \theta_1 \\ \theta_3 e^{-\theta_4 (\tilde{d} - \theta_1)} & \text{if } \tilde{d} > \theta_1 \end{cases} \tag{17}$$

$$W_{ph} = W_{as} + \theta_5 W_{peak} \tag{18}$$

$$W_{eff} = W_{as} + (1 + \theta_6 h_d) W_{peak} \tag{19}$$

$$\tilde{d}_{ph} = \begin{cases} \tilde{d} - 2W_{ph} & \text{if } 2W_{ph} \leq \tilde{d} - \theta_8 \\ \theta_8 & \text{otherwise} \end{cases} \tag{20}$$

$$\tilde{d}_{eff} = \begin{cases} \tilde{d} - 2W_{eff} & \text{if } 2W_{eff} \leq \tilde{d} - \theta_8 \\ \theta_8 & \text{otherwise} \end{cases} \tag{21}$$

where (20) and (21) incorporates the assumption that the maximal layer thickness in a vessel with RBC passage corresponds to the vessel diameter reduced by the minimum diameter of a maximally elongated RBC divided by 2, corresponding to $\theta_8/2$.

Coefficients used in axillary functions (16) to (21).

coefficient	θ_0	θ_1	θ_2	θ_3	θ_4	θ_5	θ_6	θ_7	θ_8
value	2.4	10.5	100.0	1.1	0.03	0.6	1.18	2.6	2.8

The Fåhraeus effect was described by[1, 3]

$$\frac{h_t}{h_d} = \left(h_d + (1 - h_d)\left(1 + z_0 e^{-z_1 \tilde{d}_{ph}} - z_2 e^{-z_3 \tilde{d}_{ph}}\right)\right)\left(\frac{\tilde{d}_{ph}}{\tilde{d}}\right)^2 \tag{22}$$

with coefficients z_i defined as in the in-vitro description (9) and with the threshold τ applied for very small diameters as in (9). Velocities were calculated as (11) and (12) using (22).

3.3 Description of the phase separation effect

The following parametric function was used to describe the phase separation effect in the cal.Vitro.ABC, cal.Vivo.ABC, and cal.Esl.ABC models. Consider a diverging bifurcation with a feeding vessel m with diameter d_m and discharge hematocrit h_m, and two daughter branches a and b with diameters d_a and d_b and fractional blood flows f_a and f_b, respectively. The fractional RBC flow r_a into daughter branch a was described by[1, 4, 3]

$$r_a = \begin{cases} 0 & \text{if } f_a < X_0 \\ 1 & \text{if } f_a > 1 - X_0 \\ \frac{1}{1+e^{-A-B\log\left(\frac{G}{1-G}\right)}} & \text{otherwise} \end{cases} \tag{23}$$

with

$$A = -\phi_0 \left(\frac{\tilde{d}_a^2 - \tilde{d}_b^2}{\tilde{d}_a^2 + \tilde{d}_b^2}\right) \frac{1 - h_m}{\tilde{d}_m} \tag{24}$$

$$B = 1 + \phi_1 \frac{1 - h_m}{\tilde{d}_m} \tag{25}$$

$$X_0 = \phi_2 \frac{1 - h_m}{\tilde{d}_m} \tag{26}$$

$$G = \frac{f_a - X_0}{1 - 2X_0}. \tag{27}$$

with diameters weighted by MCV differences as in the viscosity descriptions[5]. The phase separation model could in principle yield daughter hematocrits exeeding 100% as blood flow propagates through

a series of diverging bifurcations depending on the specific bifurcation geometries and blood flow distributions. Hematocrit was therefore limited by an upper threshold ϕ_3, and hematocrit in the other branch was then calculated by mass conservation[5]. In practice, this threshold was very rarely active in the cal.Vitro.ABC, cal.Vivo.ABC, and cal.Esl.ABC models at convergence as shown in Table 9 in File S2.

The two networks contained a smaller number of nodes of degree greater than 3. For these branch points, in case of a diverging bifurcation with more than one feeding vessel, or if there were more than two draining vessels, the fractional RBC flow was assumed to partition according to fractional blood flow, i.e. draining hematocrits all equaled feeding hematocrit.

Coefficients ϕ_0 to ϕ_2 were estimated by [9] based on calibration against measurements in rats. These coefficients (10.11, 5.89, 0.09) have been rescaled (multiplied) by a factor of $(92/55)^{1/3}$, corresponding to $MCV_h = 92$ and $MCV_{rat} = 55$ [1] to account for the division with scaled diameters $\tilde{d}$ in the axillary functions (24) to (26). Coefficients used in the phase separation model, (23) to (27), were thereby:

coefficient	ϕ_0	ϕ_1	ϕ_2	ϕ_3
value	12.00	6.99	0.11	0.9

4 Hematocrit iterations

To account for the interdependence of blood flow distributions and hematocrit distributions in blood flow simulations, an iterative procedure was employed. This procedure alternated between estimating the blood flow distribution from a given hematocrit distribution and estimating the hematocrit distribution from the resulting blood flow distribution[7].

Initially, discharge hematocrit was set to 40% across all segments. The first step of the iterative approach involved computing segment viscosities and corresponding hydraulic resistances. Solving the governing system of equations yielded a prediction of blood flow throughout the network. In the second step, the phase separation model was used to update hematocrit throughout the network. These two steps provided predicted blood flows q^i and hematocrits hct^i for iteration i. The steps were then repeated to provide updated predictions based on the new hematocrit distribution.

Sucessive-over-relaxation (SOR) [7, 8, 5] was utilized to provide smooth optimization paths, stabilizing and accelerating convergence. Specifically, the hematocrit used to compute viscosities and corresponding resistances in the beginning of the first step in iteration $i+1$ was calculated as a weighted average of hct^i and hct^{i-1} by $hct^* = \alpha hct^i + (1-\alpha)hct^{i-1}$, with α being the relaxation factor. hct^* was then used to update viscosities and resistances, and blood flow equations were solved to yield predicted blood flows f^{i+1}. The second step was then conducted to yield predicted hematocrits hct^{i+1}. The relaxation factor α was initialized to 1 and was exponentially decreased throughout iterations by updating it according to $\alpha = 0.8\alpha$ at every 10'th iteration.

The two steps in the iterative approach were iterated until convergence. Convergence was monitored by evaluating the relative change in predicted blood flow at iteration i

$$\delta^i_{flow_b} = \left| \frac{q^i - q^{i-1}}{q_{mean}} \right| \tag{28}$$

where

$$q_{mean} = \left(q^i + q^{i-1}\right)/2. \tag{29}$$

The relative change (28) was calculated for all individual segments. However, segment subscripts are not shown in (28) and (29) to keep the notation uncluttered. Three thresholds were utilized to judge convergence. The first threshold $\zeta = 10^{-6} nL/min$ was used to identify segments with very low blood flow rate. Examples of such segments can be identified in Figure 11 in File S3 where the corresponding segments belong to a few segment communities that actually are interior dead-end loops. Conceptually, these segments have zero flow, but numerically they will have non-zero flow. Consequently, such segments could potentially have very high relative error resulting in convergence failure. Hence, segments with $|q_{mean}| < \zeta$, in a given iteration, was disregarded in convergence assessment for that iteration. The second convergence threshold $\epsilon = 10^{-3}$ was applied to the remaining segments, and the number of segments N_ϵ with a relative change $\delta^i_{flow_b} > \epsilon$ was counted. Finally, N_ϵ was compared to the third convergence threshold ω and convergence was decided if $N_\epsilon \leq \omega$. Setting $\omega = 0$ requires that the relative change in all segments fall below ϵ, whereas setting $\omega > 0$ allows a given number of segments to exceed the second convergence threshold. $\omega = 0$ was utilized in the study. However, for future reference, the analysis was repeated with $\omega = 10$ allowing a few (ten) segments to exceed the convergence threshold. Table 9 in File S2 shows that setting $\omega = 10$ led to a substantial acceleration in convergence speed.

5 Probabilistic uncertainty quantification in linear forward models - relationship with sensitivity analysis

The partial derivatives of blood flows $\mathbf{q}$ in (Eq 8, main text) with respect to the uncertain boundary variables in $\mathbf{b}_\mathcal{A}$ reads

$$\frac{\partial \mathbf{q}}{\partial \mathbf{b}_\mathcal{A}} = \mathbf{H}_\mathcal{A} \tag{30}$$

Consequently, the individual columns in $\mathbf{H}_\mathcal{A}$ quantify the sensitivity of segment blood flows with respect to the corresponding boundary variables (in columns). In case of a diagonal covariance matrix $\mathbf{\Sigma}_A$, it follows that the diagonal of the covariance matrix $\mathbf{\Omega}$ in (Eq 11, main text) is equivalent to first squaring the sensitivities in (30), then scaling (multiplying) each column according to the variances found along corresponding diagonal element of $\mathbf{\Sigma}_\mathcal{A}$, and finally summing across columns to accumulate contributions of individual scaled sensitivities. The squaring of individual sensitivities is commonly done in sensitivity analysis to avoid cancellation effects when accumulating across single sensitivities. The partial derivative in (30) quantifies changes in blood flow per unit change in the boundary variables. Scaling by the variances associated with individual boundary variables is therefore useful since it allows assigning varying levels of uncertainty across the uncertain boundary variables. Furthermore, it allows boundary variables with different physical units to be considered, which would be the case if both pressure boundary conditions and flow boundary conditions are modeled as uncertain parameters.

6 Bayesian calibration

6.1 Likelihood functions and prior distributions governing their parameters

To formulate the likelihood function, the observations (target values) were partitioned into two observation types: y_{vel} (target RBC velocities) and y_{dir} (target flow directions) with dimensions n_{vel}

and n_{dir}, respectively. Following our previous work[6, 9], it was assumed that the likelihood function decomposes as $L(\mathbf{x}|\mathbf{y}, m) = L_{vel}(\mathbf{x}|\mathbf{y}_{vel}, m) \times L_{dir}(\mathbf{x}|\mathbf{y}_{dir}, m)$.

RBC velocity errors were assumed to be Gaussian distributed, uncorrelated, and with identical variances

$$L_{vel}(\mathbf{x}|\mathbf{y}_{vel}, m) \quad \propto \quad \prod_{j=1}^{n_{vel}} \frac{1}{\sigma} \exp\left(-\frac{1}{2\sigma^2}(y_{vel,j} - f_{vel,j})^2\right), \tag{31}$$

where $f_{vel,j}$ denotes predicted velocity and σ denote the standard deviation of velocity errors. The prior governing the velocity errors was modeled by a scaled inverse chi-square distribution

$$p\left(\sigma^2|n_0, s_0^2\right) \propto \frac{1}{\sigma^{n_0+2}} \exp\left(-\frac{n_0 s_0^2}{2\sigma^2}\right), \tag{32}$$

with $n_0 = 1$ and $s_0^2 = 10^{-3}$ being hyper-parameters. The combination of the Gaussian error model with the scaled inverse chi-square distributions allows for an analytical solution to the posterior of the noise variance and for integrating the noise variance out of the inference equations by analytical means[10, 6].

The likelihood associated with flow direction was modelled as a sequence of independent Bernoulli trails[6]

$$L_{dir}(\mathbf{x}|\mathbf{y}_{dir}, m) \quad = \quad \prod_{j=1}^{n_{dir}} \gamma^{\mathbb{1}\left(y_{dir,j}=f_{dir,j}\right)} (1-\gamma)^{\mathbb{1}\left(y_{dir,j}\neq f_{dir,j}\right)}, \tag{33}$$

where γ is the probability of success, with success defined to be the event of correspondence between the observed and prediction flow direction, and $\mathbb{1}(\cdot)$ is the indicator function that returns one if its argument is true and zero otherwise. The prior governing the probability γ for predicting the correct flow direction was modeled as a beta distribution[6]

$$p(\gamma|\alpha, \beta) \propto \gamma^{\alpha-1}(1-\gamma)^{\beta-1}, \tag{34}$$

with $\alpha = 1000$ and $\beta = 1$ being hyper-parameters. The choice of a beta distribution permits an analytical solution to the posterior governing the probability of predicting correct flow direction[6].

6.2 Prior distributions governing boundary pressures

Let $\mathbf{x}$ denote a vector with n_x elements corresponding to the unknown pressure boundary conditions. The prior governing the pressure boundary conditions was modeled by scaled shifted beta distributions[6]

$$p(\mathbf{x}|\boldsymbol{\alpha}, \boldsymbol{\beta}, \mathbf{a}, \mathbf{b}) \propto \prod_{j=1}^{n_x} (x_j - a_j)^{\alpha_j - 1} (b_j - x_j)^{\beta_j - 1}, \tag{35}$$

where $\boldsymbol{\alpha}$ and $\boldsymbol{\beta}$ are shape hyper-parameters and $\mathbf{a}$ and $\mathbf{b}$ are hyper-parameters defining bounds of feasible parameter spaces (physiological plausible intervals). Hyper-parameters were defined by

**Vessel category**	parameter	cal.Vitro.ABC	cal.Esl.ABC	cal.Vivo.ABC
SA and DA+A, $> 20\mu$m	a/b/α/β	30/80/4/4	50/100/4/4	50/100/4/4
SA and DA+A, $\leq 20\mu$m	a/b/α/β	15/80/3/5	15/100/3/5	15/100/3/5
SV and AV+V, $> 20\mu$m	a/b/α/β	5/15/4/4	5/15/4/4	5/15/4/4
SV and AV+V, $\leq 20\mu$m	a/b/α/β	10/30/3/3	10/30/3/3	10/30/3/3
C and UNK	a/b/α/β	-15/15/7/7	-15/15/7/7	-15/15/7/7

The physical unit for parameters a and b is mmHg. Bounary nodes for categories C and UNK were defined as relative pressure boundary conditions. SA: surface arteriole, DA: decending arteriole, A: arteriole, C: capillary, V: venule, AV: ascending venule, SV: surface venule, UNK: unknown.

6.3 DREAM(ZS) algorithmic settings

Algorithmic settings used in DREAM(ZS) are provided below. Further details about these algorithmic variables and the DREAM(ZS) algorithm can be found in [11] and the references therein.

**Description**	**Symbol**	**Setting**
Samples in initial archive	m_0	100
Rate at which samples are appended to external archive	k	100
Number of chain iterations	T	2×10^6
Number of chains	N	5
Number of chain pairs used to generate a jump (jump rate)	δ	3
Number of crossover probabilities (no CR adaptation)	n_{cr}	3
Probability of jumps between disconnected modes of target distribution	$p_{(\gamma=1)}$	0.2
Probability of snooker update	-	0.1
Ergodicity variable used in proposal generation	ζ	$\mathcal{N}_{d*}\left(0, 10^{-6}\right)$
Randomization variable used in proposal generation	λ	$\mathcal{U}[-0.1, 0.1]$
Snooker jump rate	γ_s	$\mathcal{U}[1.2, 2.2]$

7 References

References

[1] A. R. Pries and T. W. Secomb. *Chapter 1 - Blood Flow in Microvascular Networks*, pages 3–36. Academic Press, San Diego, 2008.

[2] A. R. Pries, T. W. Secomb, T. Gessner, M. B. Sperandio, J. F. Gross, and P. Gaehtgens. Resistance to blood flow in microvessels in vivo. *Circ Res*, 75(5):904–15, 1994.

[3] A. R. Pries and T. W. Secomb. Microvascular blood viscosity in vivo and the endothelial surface layer. *Am J Physiol Heart Circ Physiol*, 289(6):H2657–64, 2005.

[4] A. R. Pries, K. Ley, M. Claassen, and P. Gaehtgens. Red cell distribution at microvascular bifurcations. *Microvascular Research*, 38(1):81–101, 1989.

[5] S. Lorthois, F. Cassot, and F. Lauwers. Simulation study of brain blood flow regulation by intra-cortical arterioles in an anatomically accurate large human vascular network: Part i: methodology and baseline flow. *Neuroimage*, 54(2):1031–42, 2011.

[6] P. M. Rasmussen, A. F. Smith, S. Sakadzic, D. A. Boas, A. R. Pries, T. W. Secomb, and L. Ostergaard. Model-based inference from microvascular measurements: Combining experimental measurements and model predictions using a bayesian probabilistic approach. *Microcirculation*, 24(4), 2017.

[7] A. R. Pries, T. W. Secomb, P. Gaehtgens, and J. F. Gross. Blood flow in microvascular networks. experiments and simulation. *Circ Res*, 67(4):826–34, 1990.

[8] R. Guibert, C. Fonta, and F. Plouraboue. Cerebral blood flow modeling in primate cortex. *J Cereb Blood Flow Metab*, 30(11):1860–73, 2010.

[9] P. M. Rasmussen, T. W. Secomb, and A. R. Pries. Modeling the hematocrit distribution in microcirculatory networks: A quantitative evaluation of a phase separation model. *Microcirculation*, 25(3):e12445, 2018.

[10] A. Gelman, J.B. Carlin, H.S. Stern, D.B. Dunson, A. Vehtari, and D.B. Rubin. *Bayesian Data Analysis, Third Edition.* Taylor & Francis, 2013.

[11] J. A. Vrugt. Markov chain monte carlo simulation using the dream software package: Theory, concepts, and matlab implementation. *Environmental Modelling & Software*, 75:273–316, 2016.
