# Supplementary material for: Blood Flow Simulation and Uncertainty Quantification in Extensive Microvascular Networks: Application to Brain Cortical Networks
Source: Microcirculation. 2025 Sep 21;32(7):e70027. doi: 10.1111/micc.70027 (PMC12450458; doi:10.1111/micc.70027)
Supplement: Supplementary file 2 — File S2: micc70027‐sup‐0002‐FileS2.pdf. [file MICC-32-e70027-s002.pdf]

Blood flow simulation and uncertainty quantification in extensive microvascular networks: Application to brain cortical networks

File S2
Supplementary tables

2025.08.19

Peter Mondrup Rasmussen[1*]
1 Center of Functionally Integrative Neuroscience, Department of Clinical Medicine, Aarhus University, Aarhus, Denmark. * Corresponding author: pmr@cfin.au.dk.

network	segType	segments	meanDia	stdDia	minDia	maxDia	meanLen	stdLen
NW1	SA	20	24.7	14.8	9.3	48.5	176.3	120.7
NW1	DA+A	920	10.0	1.1	6.1	28.0	51.1	48.1
NW1	C	8100	4.5	1.0	2.5	9.0	76.0	57.2
NW1	V+AV	703	10.7	1.9	5.8	25.8	41.8	29.7
NW1	SV	54	33.9	14.5	10.5	62.8	77.6	59.3
NW1	UNK	1076	9.6	0.7	9.0	13.5	42.4	35.4
NW2	SA	34	37.3	20.2	12.3	64.3	163.1	135.4
NW2	DA+A	923	10.7	2.0	6.3	26.9	54.3	64.4
NW2	C	15672	4.3	0.9	2.5	9.0	63.9	44.7
NW2	V+AV	1674	11.4	2.8	5.7	32.3	34.2	26.8
NW2	SV	74	30.3	12.4	14.3	54.3	64.5	44.9
NW2	UNK	941	9.7	0.6	9.0	14.3	26.6	18.2

Table 1 (File S2). Segment morphology.

Summary statistics grouped by networks and segment types. Diameters (*Dia) and lengths (*Len) are in microns.

Abbreviations: SA: surface arteriole, DA: descending arteriole, A: arteriole, C: capillary, V: venule, AV: ascending venule, SV: surface venule, UNK: unknown. mean*: mean, std*: standard deviation, min*: minimum, max*:maximum.

layer	NW1			NW2		
	**C**	**UNK**	**REF**	**C**	**UNK**	**REF**
AL1	164	22	1071	263	11	2287
AL2	165	15	860	236	2	1803
AL3	210	19	989	251	6	1855
AL4	161	21	704	241	18	1722
AL5	129	28	661	253	16	922
AL6	132	30		161	4	

Table 2 (File S2). Boundary node information for the adaptive approach. Number of capillary (C) and unknown (UNK) boundary nodes that had their boundary pressures assigned by the adaptive approach. REF denote the number of reference nodes. No reference nodes in AL6 (reference nodes in AL5 were used also for AL6). Node statistics are grouped by network (NW1, NW2) and by analysis layer (AL).

Metric: pressure. Unit: mmHg. Segment type: All.

model	nw	mean	median	sd	q2.5	q12.5	q25	q75	q87.5	q97.5
ref.Data	NW1	21.49	21.79	4.89	11.68	16.07	18.84	23.71	26.28	31.70
ref.Vitro	NW1	21.55	21.84	5.00	11.61	16.00	18.80	23.79	26.33	32.21
ref.Vitro.ABC	NW1	21.64	22.18	4.68	11.76	16.44	19.31	23.73	25.23	31.71
cal.Vitro.ABC	NW1	23.23	23.59	4.03	15.13	18.89	21.09	24.91	26.35	32.33
cal.Esl.ABC	NW1	31.21	32.18	7.77	14.86	22.24	27.08	34.93	37.84	46.70
cal.Vivo.ABC	NW1	35.10	36.26	10.02	14.00	23.14	29.41	40.20	44.41	55.79
ref.Data	NW2	26.15	26.45	6.09	14.14	18.95	22.57	29.55	31.86	38.78
ref.Vitro	NW2	26.21	26.45	6.34	13.97	18.74	22.36	29.72	32.34	39.67
ref.Vitro.ABC	NW2	28.50	29.61	6.11	14.82	21.10	25.32	31.82	33.77	40.15
cal.Vitro.ABC	NW2	25.27	26.12	3.43	17.47	21.13	23.57	26.95	27.86	31.19
cal.Esl.ABC	NW2	29.98	30.78	6.41	16.49	22.24	26.86	32.82	35.34	43.15
cal.Vivo.ABC	NW2	32.95	33.21	9.35	14.97	21.72	28.10	37.10	42.35	54.21

Metric: hct_d. Unit: %. Segment type: All.

model	nw	mean	median	sd	q2.5	q12.5	q25	q75	q87.5	q97.5
ref.Data	NW1	39.17	38.21	24.63	0.00	5.23	19.51	59.39	71.02	82.08
ref.Vitro	NW1									
ref.Vitro.ABC	NW1									
cal.Vitro.ABC	NW1	32.00	33.24	9.87	8.23	20.72	26.67	38.52	41.26	49.64
cal.Esl.ABC	NW1	34.12	35.30	9.51	11.48	23.66	29.11	39.71	42.97	52.15
cal.Vivo.ABC	NW1	33.17	34.31	9.85	11.01	22.14	27.21	39.46	42.49	52.62
ref.Data	NW2	37.97	37.27	25.56	0.00	3.03	16.01	59.07	71.87	82.84
ref.Vitro	NW2									
ref.Vitro.ABC	NW2									
cal.Vitro.ABC	NW2	32.89	34.07	10.02	10.61	21.26	26.87	39.15	42.67	51.93
cal.Esl.ABC	NW2	34.58	35.72	9.13	13.74	24.37	29.37	39.98	43.50	51.78
cal.Vivo.ABC	NW2	33.32	34.26	9.32	12.94	22.55	27.67	39.39	42.52	51.20

Metric: flow_b. Unit: nl/min. Segment type: All.

model	nw	mean	median	sd	q2.5	q12.5	q25	q75	q87.5	q97.5
ref.Data	NW1	4.35	0.51	42.21	0.01	0.08	0.18	1.39	3.25	27.16
ref.Vitro	NW1	4.23	0.47	41.96	0.01	0.06	0.16	1.32	3.15	26.37
ref.Vitro.ABC	NW1	4.88	0.60	42.54	0.01	0.08	0.20	1.77	4.35	31.36
cal.Vitro.ABC	NW1	6.53	0.55	64.92	0.01	0.08	0.19	1.64	4.32	31.44
cal.Esl.ABC	NW1	4.81	0.34	52.26	0.01	0.05	0.12	0.93	2.32	18.85
cal.Vivo.ABC	NW1	5.81	0.29	69.55	0.01	0.05	0.11	0.82	2.19	20.56
ref.Data	NW2	7.99	0.72	93.90	0.03	0.11	0.25	2.10	5.09	39.98
ref.Vitro	NW2	7.74	0.66	92.40	0.02	0.09	0.21	2.00	4.83	38.75
ref.Vitro.ABC	NW2	8.43	0.72	94.01	0.02	0.10	0.23	2.21	5.45	45.05
cal.Vitro.ABC	NW2	6.80	0.47	74.14	0.01	0.07	0.15	1.45	3.79	35.17
cal.Esl.ABC	NW2	5.00	0.30	67.30	0.01	0.05	0.11	0.89	2.23	23.25
cal.Vivo.ABC	NW2	7.13	0.29	135.70	0.01	0.05	0.11	0.83	2.16	25.34

Metric: vel_b. Unit: mm/s. Segment type: All.

model	nw	mean	median	sd	q2.5	q12.5	q25	q75	q87.5	q97.5
ref.Data	NW1	0.94	0.42	1.79	0.01	0.07	0.16	0.99	1.80	5.29
ref.Vitro	NW1	0.88	0.38	1.75	0.01	0.06	0.14	0.92	1.71	5.01
ref.Vitro.ABC	NW1	1.11	0.49	2.14	0.01	0.08	0.18	1.20	2.13	6.15
cal.Vitro.ABC	NW1	1.10	0.46	2.22	0.01	0.07	0.17	1.14	2.19	6.15
cal.Esl.ABC	NW1	0.66	0.29	1.43	0.01	0.05	0.11	0.67	1.23	3.78
cal.Vivo.ABC	NW1	0.64	0.25	1.68	0.01	0.04	0.10	0.57	1.05	3.75
ref.Data	NW2	1.50	0.73	2.57	0.03	0.13	0.28	1.73	3.02	7.53
ref.Vitro	NW2	1.40	0.65	2.48	0.02	0.11	0.24	1.58	2.84	7.19
ref.Vitro.ABC	NW2	1.57	0.73	2.93	0.02	0.12	0.26	1.74	3.13	8.15
cal.Vitro.ABC	NW2	1.10	0.49	2.45	0.01	0.08	0.18	1.15	2.06	5.85
cal.Esl.ABC	NW2	0.68	0.32	1.42	0.01	0.06	0.12	0.72	1.23	3.57
cal.Vivo.ABC	NW2	0.70	0.31	1.87	0.01	0.06	0.13	0.66	1.14	3.88

Metric: shear stress. Unit: dyn/(cm*cm). Segment type: All.

model	nw	mean	median	sd	q2.5	q12.5	q25	q75	q87.5	q97.5
ref.Data	NW1	24.81	11.82	42.14	0.23	1.55	4.04	28.71	49.89	128.67
ref.Vitro	NW1	26.21	11.93	47.01	0.22	1.56	3.99	29.86	52.44	139.28
ref.Vitro.ABC	NW1	31.66	15.11	52.82	0.37	2.29	5.29	36.45	64.11	166.41
cal.Vitro.ABC	NW1	24.84	11.31	45.13	0.28	1.65	3.92	28.70	49.82	129.64
cal.Esl.ABC	NW1	49.45	25.47	82.67	0.57	3.59	8.81	59.42	97.34	245.25
cal.Vivo.ABC	NW1	68.39	35.38	119.11	0.52	3.84	10.78	81.19	136.89	332.74
ref.Data	NW2	40.65	23.54	52.89	0.65	3.68	8.34	53.13	87.13	175.59
ref.Vitro	NW2	43.32	24.27	59.65	0.66	3.78	8.62	56.05	92.05	190.85
ref.Vitro.ABC	NW2	46.98	26.60	65.65	0.72	4.26	9.61	60.78	98.08	205.07
cal.Vitro.ABC	NW2	25.14	13.88	37.75	0.36	2.06	4.83	32.41	51.82	111.75
cal.Esl.ABC	NW2	52.02	30.93	71.26	0.93	4.93	10.85	68.02	107.38	220.64
cal.Vivo.ABC	NW2	82.82	50.49	108.87	1.22	6.98	16.92	110.56	173.25	342.43

Metric: vel_c. Unit: mm/s. Segment type: All.

model	nw	mean	median	sd	q2.5	q12.5	q25	q75	q87.5	q97.5
ref.Data	NW1	1.28	0.54	2.55	0.02	0.10	0.21	1.29	2.37	7.55
ref.Vitro	NW1									
ref.Vitro.ABC	NW1									
cal.Vitro.ABC	NW1	1.52	0.63	3.10	0.02	0.10	0.24	1.55	3.02	8.77
cal.Esl.ABC	NW1	1.16	0.50	2.54	0.01	0.08	0.19	1.14	2.14	6.83
cal.Vivo.ABC	NW1	0.89	0.34	2.35	0.01	0.06	0.14	0.79	1.47	5.41
ref.Data	NW2	2.03	0.92	3.70	0.04	0.17	0.35	2.22	4.03	11.13
ref.Vitro	NW2									
ref.Vitro.ABC	NW2									
cal.Vitro.ABC	NW2	1.51	0.66	3.46	0.02	0.11	0.24	1.54	2.79	8.23
cal.Esl.ABC	NW2	1.17	0.53	2.51	0.02	0.09	0.20	1.20	2.14	6.48
cal.Vivo.ABC	NW2	0.97	0.42	2.61	0.01	0.08	0.17	0.89	1.57	5.56

Table 3 (File S2). Summary statistics computed across segment type: All.

q2.5, q12.5, q25, q75, q87.5, q97.5 are distribution percentiles. Refer to the main text for model abbreviations. Hematocrit and RBC velocities are not summarized for the ref.Vitro and ref.Vitro.ABC models, since the biphasic nature of blood was not incorporated into these models (refer to section 2.6.1 in the main text for further details).

Metric: pressure. Unit: mmHg. Segment type: SA.										
model	nw	mean	median	sd	q2.5	q12.5	q25	q75	q87.5	q97.5
ref.Data	NW1	43.03	46.91	9.11	30.17	31.83	32.34	50.64	51.49	53.26
ref.Vitro	NW1	43.10	47.00	9.03	30.62	31.94	32.36	50.65	51.50	53.27
ref.Vitro.ABC	NW1	43.09	46.90	9.03	30.64	31.94	32.36	50.64	51.50	53.27
cal.Vitro.ABC	NW1	44.94	42.64	5.91	38.18	40.05	41.29	46.64	53.49	56.88
cal.Esl.ABC	NW1	70.40	71.06	5.34	58.64	65.45	68.09	73.97	75.92	77.31
cal.Vivo.ABC	NW1	75.86	78.23	8.86	62.76	63.88	66.94	83.91	85.12	86.24
ref.Data	NW2	51.53	51.90	6.62	35.55	44.33	48.21	57.78	58.36	59.05
ref.Vitro	NW2	51.57	51.96	6.62	35.55	44.43	48.22	57.80	58.38	59.05
ref.Vitro.ABC	NW2	51.60	52.02	6.62	35.55	44.54	48.23	57.82	58.38	59.05
cal.Vitro.ABC	NW2	41.78	39.78	4.74	36.52	37.74	38.60	45.24	48.67	51.38
cal.Esl.ABC	NW2	61.71	58.77	7.57	54.04	56.56	57.59	61.46	73.60	78.93
cal.Vivo.ABC	NW2	70.79	68.68	8.26	58.34	63.90	66.04	73.47	83.18	87.94

Metric: hct_d. Unit: %. Segment type: SA.										
model	nw	mean	median	sd	q2.5	q12.5	q25	q75	q87.5	q97.5
ref.Data	NW1	39.66	39.16	4.73	32.51	35.23	37.33	42.10	42.28	48.53
ref.Vitro	NW1									
ref.Vitro.ABC	NW1									
cal.Vitro.ABC	NW1	39.90	40.00	1.12	37.55	38.84	39.30	40.52	40.80	41.77
cal.Esl.ABC	NW1	39.81	40.00	1.02	37.90	38.45	39.37	40.30	40.74	41.34
cal.Vivo.ABC	NW1	39.69	40.00	1.29	37.01	38.13	39.28	40.38	40.80	41.23
ref.Data	NW2	36.40	37.29	5.58	19.22	34.04	35.55	38.55	41.52	42.48
ref.Vitro	NW2									
ref.Vitro.ABC	NW2									
cal.Vitro.ABC	NW2	40.63	40.40	1.39	38.61	39.73	40.03	40.69	42.02	43.87
cal.Esl.ABC	NW2	40.73	40.48	1.80	37.94	39.50	40.04	40.91	41.95	45.16
cal.Vivo.ABC	NW2	40.27	40.39	1.79	36.76	38.04	39.60	40.92	41.62	43.92

Metric: flow_b. Unit: nl/min. Segment type: SA.										
model	nw	mean	median	sd	q2.5	q12.5	q25	q75	q87.5	q97.5
ref.Data	NW1	556.89	313.14	702.42	5.49	12.03	25.01	638.78	1426.50	2210.09
ref.Vitro	NW1	554.23	308.57	701.55	4.48	9.63	23.97	637.55	1425.17	2203.08
ref.Vitro.ABC	NW1	555.61	311.93	702.28	4.46	9.54	23.76	635.59	1428.29	2206.39
cal.Vitro.ABC	NW1	380.29	219.63	412.69	20.24	23.66	31.94	670.46	874.89	1258.21
cal.Esl.ABC	NW1	416.81	186.73	472.90	5.96	8.79	15.94	640.80	1061.50	1374.79
cal.Vivo.ABC	NW1	676.47	253.66	818.78	3.88	4.43	14.82	1323.69	1855.93	2256.79
ref.Data	NW2	1298.45	561.02	1582.54	34.38	69.45	108.62	2479.60	3750.76	4725.66
ref.Vitro	NW2	1279.62	558.88	1564.48	34.01	68.32	99.90	2413.23	3745.21	4667.87
ref.Vitro.ABC	NW2	1269.66	554.08	1557.32	33.16	67.27	91.48	2376.87	3745.29	4634.71
cal.Vitro.ABC	NW2	1037.40	494.33	1152.33	48.95	115.66	199.42	1375.14	3000.87	3537.17
cal.Esl.ABC	NW2	1000.41	467.97	1107.67	39.67	86.06	161.09	1498.56	2872.51	3243.69
cal.Vivo.ABC	NW2	1999.93	567.44	2472.63	47.30	85.94	201.21	3996.16	6042.66	6509.29

Metric: vel_b. Unit: mm/s. Segment type: SA.										
model	nw	mean	median	sd	q2.5	q12.5	q25	q75	q87.5	q97.5
ref.Data	NW1	12.61	6.81	10.45	1.32	2.97	4.54	19.95	22.25	33.39
ref.Vitro	NW1	12.42	6.81	10.55	1.08	2.37	4.19	19.90	21.81	33.41
ref.Vitro.ABC	NW1	12.46	6.77	10.58	1.07	2.35	4.27	19.87	22.32	33.37
cal.Vitro.ABC	NW1	9.04	8.96	3.31	4.91	5.54	6.18	11.19	12.74	15.05
cal.Esl.ABC	NW1	8.04	8.86	4.31	1.33	2.17	3.93	11.87	12.40	13.60
cal.Vivo.ABC	NW1	11.52	10.41	8.28	0.94	1.09	3.02	19.94	21.53	23.67
ref.Data	NW2	17.23	12.44	18.99	0.74	1.92	4.04	23.48	33.46	77.78
ref.Vitro	NW2	17.04	12.15	18.99	0.65	1.88	4.04	23.20	33.22	77.92
ref.Vitro.ABC	NW2	16.91	11.98	18.99	0.59	1.84	3.95	23.04	32.96	77.99
cal.Vitro.ABC	NW2	12.25	11.65	7.20	3.92	5.73	7.21	15.34	17.48	29.72
cal.Esl.ABC	NW2	10.75	10.39	4.83	3.18	5.46	7.26	14.01	15.70	18.71
cal.Vivo.ABC	NW2	16.84	15.06	9.45	3.79	6.58	9.47	25.09	31.03	33.43

Metric: shear stress. Unit: dyn/(cm*cm). Segment type: SA.										
model	nw	mean	median	sd	q2.5	q12.5	q25	q75	q87.5	q97.5
ref.Data	NW1	85.62	65.91	88.18	13.20	21.21	29.30	88.66	161.31	298.43
ref.Vitro	NW1	83.41	65.57	88.81	12.06	19.90	26.84	88.41	161.25	295.84
ref.Vitro.ABC	NW1	83.63	65.35	89.12	12.24	20.18	26.61	88.53	161.17	298.68
cal.Vitro.ABC	NW1	70.30	56.86	38.00	28.66	40.82	45.67	77.83	125.40	150.92
cal.Esl.ABC	NW1	116.46	74.28	88.94	24.93	55.57	58.89	134.74	238.53	313.94
cal.Vivo.ABC	NW1	113.82	87.93	88.38	40.07	46.96	57.58	121.47	182.88	333.03
ref.Data	NW2	90.22	45.20	106.20	2.40	8.43	19.68	117.32	191.01	373.26
ref.Vitro	NW2	89.32	44.72	106.20	2.34	8.33	19.83	111.83	189.89	373.98
ref.Vitro.ABC	NW2	88.64	44.92	106.17	2.15	8.17	19.58	108.60	188.39	374.57
cal.Vitro.ABC	NW2	73.46	54.26	68.02	20.74	30.59	37.49	73.24	110.90	269.65
cal.Esl.ABC	NW2	98.47	61.35	92.07	25.11	43.64	51.20	89.28	221.54	325.87
cal.Vivo.ABC	NW2	120.55	95.77	90.25	51.12	54.61	67.17	111.43	259.06	349.87

Metric: vel_c. Unit: mm/s. Segment type: SA.										
model	nw	mean	median	sd	q2.5	q12.5	q25	q75	q87.5	q97.5
ref.Data	NW1	17.30	9.42	14.57	1.96	4.07	6.04	26.95	32.39	46.62
ref.Vitro	NW1									
ref.Vitro.ABC	NW1									
cal.Vitro.ABC	NW1	12.22	11.67	4.41	6.82	7.89	8.75	14.73	16.87	21.47
cal.Esl.ABC	NW1	12.42	13.30	6.06	2.86	4.29	7.76	17.12	19.11	21.15
cal.Vivo.ABC	NW1	15.06	13.70	10.33	1.37	1.57	4.59	24.61	27.22	29.64
ref.Data	NW2	23.35	15.21	26.07	0.98	2.55	5.23	29.52	47.83	104.96
ref.Vitro	NW2									
ref.Vitro.ABC	NW2									
cal.Vitro.ABC	NW2	15.78	15.04	9.95	5.53	7.15	9.41	19.33	20.97	41.01
cal.Esl.ABC	NW2	15.52	14.88	7.42	5.41	7.80	10.10	19.33	21.67	31.14
cal.Vivo.ABC	NW2	21.09	19.63	10.80	5.18	8.95	12.44	30.23	37.04	39.17

Table 4 (File S2). Summary statistics computed across segment type: SA.

q2.5, q12.5, q25, q75, q87.5, q97.5 are distribution percentiles. Refer to the main text for model abbreviations. Hematocrit and RBC velocities are not summarized for the ref.Vitro and ref.Vitro.ABC models, since the biphasic nature of blood was not incorporated into these models (refer to section 2.6.1 in the main text for further details).

Metric: pressure. Unit: mmHg. Segment type: SV.

model	nw	mean	median	sd	q2.5	q12.5	q25	q75	q87.5	q97.5
ref.Data	NW1	10.63	10.38	0.76	10.04	10.13	10.23	10.50	11.40	12.82
ref.Vitro	NW1	10.62	10.36	0.75	10.04	10.12	10.22	10.48	11.39	12.79
ref.Vitro.ABC	NW1	10.66	10.41	0.78	10.04	10.13	10.24	10.53	11.47	12.91
cal.Vitro.ABC	NW1	13.77	13.74	1.08	11.98	12.61	13.07	14.27	14.89	16.19
cal.Esl.ABC	NW1	12.16	11.91	1.27	10.21	10.82	11.38	12.95	13.76	14.87
cal.Vivo.ABC	NW1	11.54	11.30	1.27	9.54	10.18	10.73	12.62	13.21	14.07
ref.Data	NW2	12.70	13.13	1.94	10.04	10.19	10.61	14.29	14.59	16.15
ref.Vitro	NW2	12.63	13.00	1.89	10.04	10.19	10.59	14.19	14.50	16.02
ref.Vitro.ABC	NW2	12.90	13.33	2.07	10.04	10.20	10.72	14.65	14.94	16.36
cal.Vitro.ABC	NW2	15.95	16.69	1.97	11.94	13.64	14.35	17.49	17.78	18.97
cal.Esl.ABC	NW2	13.52	13.58	1.50	10.28	11.92	13.15	14.02	14.75	16.95
cal.Vivo.ABC	NW2	12.19	11.95	1.64	8.74	11.11	11.44	12.94	13.56	16.02

Metric: hct_d. Unit: %. Segment type: SV.

model	nw	mean	median	sd	q2.5	q12.5	q25	q75	q87.5	q97.5
ref.Data	NW1	39.41	39.69	5.92	31.06	35.38	36.60	42.67	45.36	47.25
ref.Vitro	NW1									
ref.Vitro.ABC	NW1									
cal.Vitro.ABC	NW1	39.28	39.71	1.28	36.49	38.08	38.91	39.98	40.01	40.75
cal.Esl.ABC	NW1	39.55	39.88	0.99	36.47	38.60	39.38	40.07	40.25	40.57
cal.Vivo.ABC	NW1	39.44	39.81	1.09	35.81	38.66	39.22	40.02	40.15	40.40
ref.Data	NW2	36.29	35.71	7.08	19.98	33.03	34.10	40.54	42.98	48.19
ref.Vitro	NW2									
ref.Vitro.ABC	NW2									
cal.Vitro.ABC	NW2	38.41	38.53	2.12	33.00	37.12	37.80	39.80	40.38	41.49
cal.Esl.ABC	NW2	38.27	38.49	2.15	33.14	36.60	37.45	39.71	40.02	40.89
cal.Vivo.ABC	NW2	37.24	37.38	2.69	31.53	35.42	36.02	39.37	39.86	40.03

Metric: flow_b. Unit: nl/min. Segment type: SV.

model	nw	mean	median	sd	q2.5	q12.5	q25	q75	q87.5	q97.5
ref.Data	NW1	142.64	115.44	116.34	6.83	28.99	52.14	201.71	239.65	446.13
ref.Vitro	NW1	139.09	112.81	112.60	6.76	28.20	49.44	199.25	233.47	428.41
ref.Vitro.ABC	NW1	149.54	132.36	118.23	6.75	30.05	55.75	212.13	246.91	455.66
cal.Vitro.ABC	NW1	514.68	219.31	623.67	19.81	44.08	101.28	444.79	1559.46	2044.95
cal.Esl.ABC	NW1	393.39	200.45	452.96	10.11	35.51	87.45	509.36	1202.30	1564.17
cal.Vivo.ABC	NW1	460.95	238.43	528.06	8.37	38.66	103.83	644.82	1425.88	1809.43
ref.Data	NW2	381.04	177.16	417.94	17.90	40.34	83.45	615.88	889.87	1471.68
ref.Vitro	NW2	368.84	173.05	405.16	18.17	41.41	82.89	581.92	843.47	1435.69
ref.Vitro.ABC	NW2	411.96	193.87	463.20	18.65	40.28	88.24	626.18	1080.44	1606.48
cal.Vitro.ABC	NW2	375.02	228.10	370.83	31.40	63.86	133.22	570.55	672.20	1371.71
cal.Esl.ABC	NW2	262.95	164.73	277.73	19.69	43.04	84.22	337.87	503.62	1049.87
cal.Vivo.ABC	NW2	292.46	189.33	312.42	17.42	42.15	86.64	371.61	534.59	1183.05

Metric: vel_b. Unit: mm/s. Segment type: SV.

model	nw	mean	median	sd	q2.5	q12.5	q25	q75	q87.5	q97.5
ref.Data	NW1	2.63	2.06	1.83	0.43	1.05	1.47	3.13	4.69	7.96
ref.Vitro	NW1	2.57	1.97	1.81	0.42	1.05	1.43	3.02	4.64	7.90
ref.Vitro.ABC	NW1	2.76	2.27	1.88	0.42	1.17	1.52	3.20	4.71	8.34
cal.Vitro.ABC	NW1	6.22	5.62	3.44	1.21	2.71	3.54	8.34	10.79	13.19
cal.Esl.ABC	NW1	5.02	5.28	2.54	0.77	1.86	3.38	6.26	8.25	10.09
cal.Vivo.ABC	NW1	5.90	6.07	3.03	0.82	1.87	4.07	7.58	9.75	11.67
ref.Data	NW2	7.38	5.72	6.02	1.00	1.48	3.37	9.98	14.32	23.79
ref.Vitro	NW2	7.16	5.47	5.82	0.98	1.51	3.28	9.51	13.54	23.22
ref.Vitro.ABC	NW2	7.84	5.79	6.66	1.15	1.66	3.74	9.90	14.86	25.94
cal.Vitro.ABC	NW2	9.04	5.63	10.21	1.95	3.07	3.45	9.00	20.75	37.23
cal.Esl.ABC	NW2	5.68	4.10	5.27	1.48	2.01	2.79	5.90	8.01	19.54
cal.Vivo.ABC	NW2	6.33	4.38	5.84	1.48	1.95	3.07	6.81	9.54	20.91

Metric: shear stress. Unit: dyn/(cm*cm). Segment type: SV.

model	nw	mean	median	sd	q2.5	q12.5	q25	q75	q87.5	q97.5
ref.Data	NW1	15.09	9.07	13.52	2.53	4.47	6.65	20.15	28.01	44.51
ref.Vitro	NW1	14.74	8.69	13.39	2.49	4.35	6.34	20.03	27.75	43.54
ref.Vitro.ABC	NW1	15.73	10.29	13.87	2.66	4.75	7.42	20.60	29.39	45.50
cal.Vitro.ABC	NW1	31.49	34.34	13.04	5.86	14.35	23.48	39.87	43.20	51.15
cal.Esl.ABC	NW1	37.48	37.13	18.86	5.40	19.98	25.50	44.85	53.89	87.67
cal.Vivo.ABC	NW1	40.82	39.12	24.43	4.70	17.79	27.12	48.57	59.83	109.55
ref.Data	NW2	38.90	33.65	29.71	4.05	8.28	16.94	53.47	73.42	112.43
ref.Vitro	NW2	37.78	31.87	28.83	3.95	8.81	16.82	51.50	69.11	110.15
ref.Vitro.ABC	NW2	40.70	35.18	31.37	4.77	9.70	17.39	52.43	80.17	116.10
cal.Vitro.ABC	NW2	57.77	35.40	79.84	7.48	14.30	20.33	49.51	98.80	320.06
cal.Esl.ABC	NW2	53.06	38.12	62.01	7.08	11.93	20.89	60.21	94.38	282.70
cal.Vivo.ABC	NW2	54.54	35.61	64.56	7.13	11.41	16.01	65.14	97.30	300.12

Metric: vel_c. Unit: mm/s. Segment type: SV.

model	nw	mean	median	sd	q2.5	q12.5	q25	q75	q87.5	q97.5
ref.Data	NW1	3.55	2.79	2.50	0.66	1.29	1.84	4.16	6.30	10.83
ref.Vitro	NW1									
ref.Vitro.ABC	NW1									
cal.Vitro.ABC	NW1	7.95	7.49	4.07	1.60	3.58	4.76	10.95	13.11	15.77
cal.Esl.ABC	NW1	7.18	7.48	3.24	1.14	2.88	5.04	9.30	11.24	12.95
cal.Vivo.ABC	NW1	7.56	7.88	3.65	1.14	2.40	5.33	9.79	12.34	13.95
ref.Data	NW2	10.20	8.18	8.18	1.30	2.00	4.53	13.56	19.19	32.37
ref.Vitro	NW2									
ref.Vitro.ABC	NW2									
cal.Vitro.ABC	NW2	12.10	7.59	14.02	2.41	4.00	4.80	11.68	26.69	52.05
cal.Esl.ABC	NW2	8.61	6.31	8.15	2.05	3.18	4.18	8.81	13.07	32.29
cal.Vivo.ABC	NW2	8.45	5.93	7.83	1.94	2.63	4.10	8.87	12.98	28.53

Table 5 (File S2). Summary statistics computed across segment type: SV.

q2.5, q12.5, q25, q75, q87.5, q97.5 are distribution percentiles. Refer to the main text for model abbreviations. Hematocrit and RBC velocities are not summarized for the ref.Vitro and ref.Vitro.ABC models, since the biphasic nature of blood was not incorporated into these models (refer to section 2.6.1 in the main text for further details).

Metric: pressure. Unit: mmHg. Segment type: DA+A.										
model	nw	mean	median	sd	q2.5	q12.5	q25	q75	q87.5	q97.5
ref.Data	NW1	25.38	23.44	5.28	19.76	21.90	22.27	27.21	29.44	43.65
ref.Vitro	NW1	25.50	23.55	5.36	20.07	22.00	22.31	27.15	29.93	43.95
ref.Vitro.ABC	NW1	25.35	23.66	5.08	20.58	22.30	22.96	25.41	28.67	43.83
cal.Vitro.ABC	NW1	27.06	25.41	4.86	21.54	22.97	24.13	28.49	32.88	39.60
cal.Esl.ABC	NW1	39.07	37.02	8.14	29.93	32.55	33.71	41.99	47.20	64.21
cal.Vivo.ABC	NW1	45.51	43.46	8.93	35.63	38.43	39.25	48.27	52.53	74.03
ref.Data	NW2	35.06	33.65	6.69	26.11	28.09	30.08	39.44	43.14	51.76
ref.Vitro	NW2	35.40	33.94	6.86	26.13	28.08	30.09	40.17	43.90	52.02
ref.Vitro.ABC	NW2	36.32	35.06	6.44	27.17	29.58	31.52	40.78	44.06	52.01
cal.Vitro.ABC	NW2	30.42	29.43	3.65	26.59	27.30	27.94	31.64	34.29	40.74
cal.Esl.ABC	NW2	40.47	38.88	7.13	31.01	33.39	35.26	44.08	48.40	57.15
cal.Vivo.ABC	NW2	48.32	46.74	9.64	34.07	37.39	40.35	55.22	59.74	68.32

Metric: hct_d. Unit: %. Segment type: DA+A.										
model	nw	mean	median	sd	q2.5	q12.5	q25	q75	q87.5	q97.5
ref.Data	NW1	44.38	43.34	24.20	0.00	13.33	27.09	65.41	75.20	81.14
ref.Vitro	NW1									
ref.Vitro.ABC	NW1									
cal.Vitro.ABC	NW1	36.82	37.71	7.48	22.12	28.10	32.05	40.92	44.69	52.30
cal.Esl.ABC	NW1	38.41	38.14	7.85	23.86	30.17	33.67	41.74	46.87	57.70
cal.Vivo.ABC	NW1	38.31	38.16	8.09	23.80	29.72	33.12	42.20	47.07	58.14
ref.Data	NW2	44.01	42.10	21.60	0.00	19.05	31.14	60.37	73.74	80.60
ref.Vitro	NW2									
ref.Vitro.ABC	NW2									
cal.Vitro.ABC	NW2	37.46	38.47	6.46	23.75	29.45	33.98	41.13	43.73	49.18
cal.Esl.ABC	NW2	37.87	38.67	6.17	24.54	30.52	34.98	41.40	44.06	49.11
cal.Vivo.ABC	NW2	36.38	37.19	6.85	21.23	28.23	33.03	40.27	42.79	50.24

Metric: flow_b. Unit: nl/min. Segment type: DA+A.										
model	nw	mean	median	sd	q2.5	q12.5	q25	q75	q87.5	q97.5
ref.Data	NW1	11.25	3.01	24.50	0.07	0.42	0.95	9.75	21.92	83.13
ref.Vitro	NW1	10.82	2.97	24.01	0.08	0.39	0.89	8.49	21.24	81.91
ref.Vitro.ABC	NW1	11.30	3.43	24.39	0.14	0.51	1.22	8.87	21.43	83.63
cal.Vitro.ABC	NW1	12.60	3.90	30.73	0.08	0.54	1.25	13.46	24.95	74.69
cal.Esl.ABC	NW1	8.18	2.26	25.95	0.07	0.34	0.75	7.19	14.32	56.05
cal.Vivo.ABC	NW1	9.32	2.36	30.20	0.11	0.37	0.79	6.93	14.96	67.73
ref.Data	NW2	28.47	10.70	54.10	0.57	2.24	4.44	28.10	53.97	192.05
ref.Vitro	NW2	27.13	10.01	51.77	0.50	2.03	3.93	26.87	51.34	159.54
ref.Vitro.ABC	NW2	26.49	10.05	50.68	0.36	1.50	3.30	27.03	50.09	152.45
cal.Vitro.ABC	NW2	22.65	8.03	43.74	0.49	1.60	3.14	20.77	45.37	144.59
cal.Esl.ABC	NW2	14.77	4.95	28.09	0.41	1.12	2.08	13.90	29.07	95.54
cal.Vivo.ABC	NW2	17.47	5.07	38.08	0.47	1.20	2.08	14.40	34.49	118.49

Metric: vel_b. Unit: mm/s. Segment type: DA+A.										
model	nw	mean	median	sd	q2.5	q12.5	q25	q75	q87.5	q97.5
ref.Data	NW1	2.04	0.68	3.65	0.02	0.10	0.22	2.01	4.60	13.16
ref.Vitro	NW1	1.96	0.67	3.59	0.02	0.09	0.21	1.71	4.21	12.44
ref.Vitro.ABC	NW1	2.05	0.75	3.62	0.03	0.12	0.27	1.92	4.29	13.42
cal.Vitro.ABC	NW1	2.34	0.85	4.10	0.02	0.12	0.29	2.85	5.44	11.49
cal.Esl.ABC	NW1	1.42	0.50	3.14	0.02	0.08	0.17	1.47	2.89	8.09
cal.Vivo.ABC	NW1	1.60	0.54	3.77	0.02	0.09	0.18	1.48	3.16	9.78
ref.Data	NW2	4.25	2.15	5.72	0.14	0.49	0.92	5.17	9.08	21.22
ref.Vitro	NW2	4.04	2.06	5.42	0.12	0.44	0.83	4.96	8.78	20.77
ref.Vitro.ABC	NW2	3.96	1.96	5.37	0.08	0.31	0.70	4.98	8.68	19.91
cal.Vitro.ABC	NW2	3.56	1.59	6.25	0.10	0.35	0.67	4.04	7.26	19.05
cal.Esl.ABC	NW2	2.22	0.99	3.58	0.09	0.25	0.44	2.48	4.60	11.20
cal.Vivo.ABC	NW2	2.62	1.00	5.44	0.10	0.26	0.46	2.68	4.99	15.01

Metric: shear stress. Unit: dyn/(cm*cm). Segment type: DA+A.										
model	nw	mean	median	sd	q2.5	q12.5	q25	q75	q87.5	q97.5
ref.Data	NW1	24.78	8.87	41.94	0.18	1.12	3.02	26.34	54.66	148.62
ref.Vitro	NW1	24.98	9.29	41.44	0.20	1.19	3.06	27.32	54.41	143.26
ref.Vitro.ABC	NW1	26.19	10.39	41.96	0.44	1.81	3.88	28.51	60.34	151.64
cal.Vitro.ABC	NW1	29.33	11.38	49.93	0.22	1.63	3.70	36.20	68.17	146.41
cal.Esl.ABC	NW1	58.95	25.62	113.35	0.82	3.77	8.67	67.56	128.71	284.97
cal.Vivo.ABC	NW1	55.55	21.98	122.67	0.62	3.38	7.22	57.34	112.43	305.62
ref.Data	NW2	48.38	26.26	64.29	1.70	6.39	12.16	59.55	98.46	210.81
ref.Vitro	NW2	47.88	27.27	66.83	1.71	6.21	12.80	56.46	95.75	209.80
ref.Vitro.ABC	NW2	46.82	26.31	65.77	1.25	4.62	10.55	57.73	97.19	203.87
cal.Vitro.ABC	NW2	42.22	19.51	75.08	1.25	4.28	8.16	47.93	80.21	226.86
cal.Esl.ABC	NW2	82.47	42.14	142.05	3.60	10.99	18.64	87.52	157.48	373.62
cal.Vivo.ABC	NW2	82.41	33.47	196.82	3.29	8.09	15.61	86.18	143.79	390.54

Metric: vel_c. Unit: mm/s. Segment type: DA+A.										
model	nw	mean	median	sd	q2.5	q12.5	q25	q75	q87.5	q97.5
ref.Data	NW1	3.03	0.96	5.42	0.03	0.13	0.32	2.89	6.93	18.41
ref.Vitro	NW1									
ref.Vitro.ABC	NW1									
cal.Vitro.ABC	NW1	3.38	1.22	5.87	0.03	0.18	0.41	4.08	7.79	16.82
cal.Esl.ABC	NW1	2.73	0.95	5.83	0.03	0.15	0.33	2.85	5.75	15.63
cal.Vivo.ABC	NW1	2.32	0.78	5.40	0.04	0.12	0.27	2.16	4.58	14.44
ref.Data	NW2	6.32	3.33	8.31	0.18	0.67	1.29	7.78	13.76	31.90
ref.Vitro	NW2									
ref.Vitro.ABC	NW2									
cal.Vitro.ABC	NW2	5.13	2.31	8.98	0.15	0.52	1.01	5.81	10.13	28.54
cal.Esl.ABC	NW2	4.19	1.94	6.70	0.18	0.49	0.87	4.67	8.57	20.89
cal.Vivo.ABC	NW2	3.82	1.53	7.84	0.16	0.39	0.67	3.86	7.25	21.85

Table 6 (File S2). Summary statistics computed across segment type: DA+A.

q2.5, q12.5, q25, q75, q87.5, q97.5 are distribution percentiles. Refer to the main text for model abbreviations. Hematocrit and RBC velocities are not summarized for the ref.Vitro and ref.Vitro.ABC models, since the biphasic nature of blood was not incorporated into these models (refer to section 2.6.1 in the main text for further details).

Metric: pressure. Unit: mmHg. Segment type: AV+V.										
model	nw	mean	median	sd	q2.5	q12.5	q25	q75	q87.5	q97.5
ref.Data	NW1	16.08	15.14	4.02	10.48	11.18	12.92	19.21	21.71	24.40
ref.Vitro	NW1	15.99	15.01	4.02	10.47	11.13	12.80	19.04	21.66	24.41
ref.Vitro.ABC	NW1	16.27	15.31	4.14	10.50	11.21	12.95	19.94	22.07	24.07
cal.Vitro.ABC	NW1	18.84	18.43	3.39	13.24	14.64	16.02	22.06	23.46	24.05
cal.Esl.ABC	NW1	21.34	20.04	6.49	11.55	13.75	16.04	27.32	30.69	32.59
cal.Vivo.ABC	NW1	20.77	18.86	7.22	10.84	12.79	15.08	26.47	32.07	34.64
ref.Data	NW2	20.07	19.19	5.67	10.71	13.41	15.58	25.05	28.17	28.93
ref.Vitro	NW2	19.89	18.99	5.68	10.69	13.22	15.39	24.74	28.11	28.89
ref.Vitro.ABC	NW2	21.21	20.40	6.27	10.73	13.76	16.02	27.92	29.93	30.61
cal.Vitro.ABC	NW2	21.37	20.77	3.65	15.00	16.95	18.50	25.36	26.31	26.76
cal.Esl.ABC	NW2	22.19	20.67	5.72	13.83	15.62	17.50	28.29	29.97	31.79
cal.Vivo.ABC	NW2	21.15	19.77	6.23	12.32	14.03	15.82	27.29	29.73	32.61

Metric: hct_d. Unit: %. Segment type: AV+V.										
model	nw	mean	median	sd	q2.5	q12.5	q25	q75	q87.5	q97.5
ref.Data	NW1	37.38	38.23	14.17	1.99	22.79	29.22	45.52	53.37	66.65
ref.Vitro	NW1									
ref.Vitro.ABC	NW1									
cal.Vitro.ABC	NW1	36.52	36.34	5.22	26.54	31.04	33.04	39.92	41.86	47.31
cal.Esl.ABC	NW1	37.67	37.94	4.36	29.22	32.98	35.11	40.16	42.07	46.40
cal.Vivo.ABC	NW1	37.17	37.62	4.50	26.92	32.37	34.98	39.91	41.36	45.37
ref.Data	NW2	35.74	36.61	15.99	0.02	17.56	26.88	43.33	53.36	70.92
ref.Vitro	NW2									
ref.Vitro.ABC	NW2									
cal.Vitro.ABC	NW2	36.68	37.06	5.18	24.92	31.16	34.32	39.53	41.14	46.97
cal.Esl.ABC	NW2	37.31	37.56	4.47	27.40	32.70	35.00	39.77	41.04	46.76
cal.Vivo.ABC	NW2	36.33	36.48	4.73	26.88	31.42	33.56	39.34	40.31	46.16

Metric: flow_b. Unit: nl/min. Segment type: AV+V.										
model	nw	mean	median	sd	q2.5	q12.5	q25	q75	q87.5	q97.5
ref.Data	NW1	12.93	5.98	17.07	0.28	1.08	2.14	16.10	31.17	62.93
ref.Vitro	NW1	12.65	5.73	16.81	0.23	0.98	2.03	15.87	30.50	61.10
ref.Vitro.ABC	NW1	13.73	7.36	17.42	0.22	1.04	2.26	17.86	31.97	60.44
cal.Vitro.ABC	NW1	17.26	8.07	40.84	0.47	1.29	2.78	20.52	35.61	63.29
cal.Esl.ABC	NW1	11.29	5.34	23.60	0.31	1.02	1.86	13.68	23.09	48.17
cal.Vivo.ABC	NW1	12.65	5.46	27.68	0.29	1.07	1.83	15.17	24.87	55.14
ref.Data	NW2	20.88	6.14	41.17	0.17	0.99	2.02	20.14	41.88	159.89
ref.Vitro	NW2	20.33	6.07	40.34	0.21	0.96	1.99	19.81	39.66	158.93
ref.Vitro.ABC	NW2	24.45	8.20	46.50	0.30	1.21	2.63	24.82	49.02	174.54
cal.Vitro.ABC	NW2	20.11	6.81	40.15	0.19	0.99	2.08	21.15	43.23	121.79
cal.Esl.ABC	NW2	12.78	4.10	24.32	0.17	0.66	1.40	13.43	26.38	86.82
cal.Vivo.ABC	NW2	14.36	4.32	27.94	0.26	0.78	1.46	14.76	29.50	100.72

Metric: vel_b. Unit: mm/s. Segment type: AV+V.										
model	nw	mean	median	sd	q2.5	q12.5	q25	q75	q87.5	q97.5
ref.Data	NW1	2.06	1.24	2.25	0.06	0.23	0.45	2.92	4.76	7.52
ref.Vitro	NW1	2.02	1.24	2.21	0.04	0.22	0.44	2.78	4.68	7.32
ref.Vitro.ABC	NW1	2.21	1.44	2.31	0.05	0.23	0.49	3.23	5.11	7.71
cal.Vitro.ABC	NW1	2.66	1.62	4.19	0.10	0.29	0.60	3.59	5.15	8.67
cal.Esl.ABC	NW1	1.73	1.07	2.40	0.07	0.24	0.41	2.33	3.44	5.38
cal.Vivo.ABC	NW1	1.93	1.08	2.85	0.06	0.24	0.41	2.65	3.76	7.38
ref.Data	NW2	2.50	1.16	3.29	0.04	0.20	0.41	3.28	5.84	11.76
ref.Vitro	NW2	2.42	1.12	3.21	0.04	0.20	0.41	3.19	5.65	11.65
ref.Vitro.ABC	NW2	2.99	1.51	3.98	0.06	0.26	0.52	4.06	6.79	12.66
cal.Vitro.ABC	NW2	2.56	1.31	4.31	0.05	0.20	0.42	3.48	5.29	9.98
cal.Esl.ABC	NW2	1.55	0.77	2.30	0.04	0.14	0.27	2.17	3.35	6.61
cal.Vivo.ABC	NW2	1.74	0.82	2.72	0.05	0.17	0.31	2.27	3.84	8.23

Metric: shear stress. Unit: dyn/(cm*cm). Segment type: AV+V.										
model	nw	mean	median	sd	q2.5	q12.5	q25	q75	q87.5	q97.5
ref.Data	NW1	22.82	14.51	24.91	0.72	2.88	5.65	33.09	49.71	84.56
ref.Vitro	NW1	22.31	14.46	24.43	0.63	2.67	5.69	31.16	48.00	84.10
ref.Vitro.ABC	NW1	24.67	16.96	25.59	0.65	3.07	5.95	36.13	52.07	83.53
cal.Vitro.ABC	NW1	31.00	20.21	47.65	1.18	3.53	7.62	40.85	54.88	111.09
cal.Esl.ABC	NW1	63.58	41.62	87.98	3.01	10.47	19.53	80.99	103.68	243.57
cal.Vivo.ABC	NW1	60.85	38.36	93.88	2.24	8.40	14.95	74.42	106.02	263.68
ref.Data	NW2	25.21	13.10	30.19	0.42	2.39	4.75	35.99	57.24	104.15
ref.Vitro	NW2	24.57	12.81	29.94	0.52	2.41	4.92	34.59	55.46	102.32
ref.Vitro.ABC	NW2	30.57	17.15	37.21	0.72	3.12	6.40	43.48	66.10	118.25
cal.Vitro.ABC	NW2	28.21	15.37	47.92	0.51	2.42	4.99	38.53	55.68	110.73
cal.Esl.ABC	NW2	48.67	29.01	70.02	1.76	5.69	10.92	64.55	96.89	191.19
cal.Vivo.ABC	NW2	46.81	23.81	75.85	1.94	5.53	10.05	57.03	95.24	206.39

Metric: vel_c. Unit: mm/s. Segment type: AV+V.										
model	nw	mean	median	sd	q2.5	q12.5	q25	q75	q87.5	q97.5
ref.Data	NW1	3.10	1.84	3.33	0.09	0.34	0.68	4.63	7.27	11.07
ref.Vitro	NW1									
ref.Vitro.ABC	NW1									
cal.Vitro.ABC	NW1	3.88	2.30	6.00	0.15	0.43	0.88	5.21	7.51	12.38
cal.Esl.ABC	NW1	3.28	2.06	4.43	0.14	0.46	0.83	4.45	6.61	10.55
cal.Vivo.ABC	NW1	2.81	1.57	4.09	0.09	0.35	0.62	3.85	5.52	10.66
ref.Data	NW2	3.82	1.87	4.92	0.07	0.31	0.65	5.01	8.96	17.42
ref.Vitro	NW2									
ref.Vitro.ABC	NW2									
cal.Vitro.ABC	NW2	3.70	1.90	6.18	0.07	0.30	0.62	5.09	7.69	14.22
cal.Esl.ABC	NW2	2.88	1.50	4.17	0.07	0.27	0.54	4.10	6.19	11.91
cal.Vivo.ABC	NW2	2.53	1.21	3.91	0.08	0.25	0.45	3.35	5.56	11.72

Table 7 (File S2). Summary statistics computed across segment type: AV+V.

q2.5, q12.5, q25, q75, q87.5, q97.5 are distribution percentiles. Refer to the main text for model abbreviations. Hematocrit and RBC velocities are not summarized for the ref.Vitro and ref.Vitro.ABC models, since the biphasic nature of blood was not incorporated into these models (refer to section 2.6.1 in the main text for further details).

Metric: pressure. Unit: mmHg. Segment type: C.										
model	nw	mean	median	sd	q2.5	q12.5	q25	q75	q87.5	q97.5
ref.Data	NW1	21.48	21.62	4.34	12.94	16.76	18.96	23.57	26.07	30.72
ref.Vitro	NW1	21.55	21.70	4.46	12.76	16.76	18.95	23.69	26.15	31.20
ref.Vitro.ABC	NW1	21.68	22.09	4.10	13.12	17.15	19.49	23.66	25.15	30.62
cal.Vitro.ABC	NW1	23.18	23.53	3.43	16.17	19.32	21.15	24.87	26.17	30.58
cal.Esl.ABC	NW1	31.15	32.01	6.59	16.74	23.63	27.35	34.75	37.33	43.59
cal.Vivo.ABC	NW1	35.15	35.97	8.64	16.33	25.25	30.00	39.79	43.54	51.98
ref.Data	NW2	26.27	26.52	5.23	15.50	20.12	23.14	29.49	31.45	36.78
ref.Vitro	NW2	26.34	26.53	5.50	15.26	19.87	23.00	29.67	31.92	37.73
ref.Vitro.ABC	NW2	28.79	29.70	5.13	16.83	22.49	26.15	31.78	33.42	38.33
cal.Vitro.ABC	NW2	25.37	26.14	2.78	18.56	21.90	24.04	26.91	27.61	29.86
cal.Esl.ABC	NW2	30.17	30.87	5.15	18.41	23.98	27.80	32.71	34.72	39.94
cal.Vivo.ABC	NW2	33.28	33.47	7.83	17.32	24.25	29.20	36.89	41.25	50.91

Metric: hct_d. Unit: %. Segment type: C.										
model	nw	mean	median	sd	q2.5	q12.5	q25	q75	q87.5	q97.5
ref.Data	NW1	38.74	37.21	25.63	0.00	3.81	16.73	60.84	71.93	83.18
ref.Vitro	NW1									
ref.Vitro.ABC	NW1									
cal.Vitro.ABC	NW1	30.80	31.94	10.38	6.89	18.77	24.84	37.66	40.89	49.93
cal.Esl.ABC	NW1	33.17	34.29	10.07	10.16	21.96	27.41	39.29	43.04	52.25
cal.Vivo.ABC	NW1	32.02	32.87	10.33	9.14	20.38	25.67	38.85	42.36	52.40
ref.Data	NW2	37.62	36.51	26.67	0.00	1.67	13.10	61.10	72.68	83.69
ref.Vitro	NW2									
ref.Vitro.ABC	NW2									
cal.Vitro.ABC	NW2	32.06	32.84	10.48	9.41	19.88	25.54	38.76	42.81	52.48
cal.Esl.ABC	NW2	33.98	34.79	9.61	12.39	23.19	28.28	39.89	43.70	52.34
cal.Vivo.ABC	NW2	32.66	33.30	9.75	11.91	21.45	26.43	39.20	42.72	51.49

Metric: flow_b. Unit: nl/min. Segment type: C.										
model	nw	mean	median	sd	q2.5	q12.5	q25	q75	q87.5	q97.5
ref.Data	NW1	0.74	0.37	1.29	0.01	0.06	0.14	0.84	1.47	3.83
ref.Vitro	NW1	0.69	0.33	1.25	0.01	0.05	0.12	0.80	1.39	3.58
ref.Vitro.ABC	NW1	0.92	0.41	2.08	0.01	0.06	0.15	1.01	1.80	4.73
cal.Vitro.ABC	NW1	0.87	0.38	1.85	0.01	0.06	0.14	0.92	1.67	4.60
cal.Esl.ABC	NW1	0.48	0.25	0.84	0.01	0.04	0.10	0.55	0.94	2.40
cal.Vivo.ABC	NW1	0.41	0.21	0.81	0.00	0.03	0.08	0.46	0.79	2.03
ref.Data	NW2	1.19	0.54	2.33	0.02	0.10	0.20	1.35	2.43	6.29
ref.Vitro	NW2	1.12	0.48	2.24	0.01	0.08	0.17	1.26	2.32	5.90
ref.Vitro.ABC	NW2	1.29	0.53	3.52	0.01	0.08	0.18	1.36	2.52	6.82
cal.Vitro.ABC	NW2	0.80	0.34	1.69	0.01	0.05	0.12	0.86	1.58	4.25
cal.Esl.ABC	NW2	0.47	0.23	0.85	0.01	0.04	0.09	0.54	0.95	2.43
cal.Vivo.ABC	NW2	0.45	0.22	0.88	0.01	0.04	0.09	0.50	0.86	2.23

Metric: vel_b. Unit: mm/s. Segment type: C.										
model	nw	mean	median	sd	q2.5	q12.5	q25	q75	q87.5	q97.5
ref.Data	NW1	0.75	0.41	1.15	0.01	0.07	0.17	0.90	1.52	3.62
ref.Vitro	NW1	0.69	0.37	1.09	0.01	0.06	0.14	0.83	1.42	3.33
ref.Vitro.ABC	NW1	0.88	0.46	1.48	0.01	0.08	0.17	1.05	1.79	4.14
cal.Vitro.ABC	NW1	0.85	0.43	1.45	0.01	0.07	0.17	0.97	1.68	4.26
cal.Esl.ABC	NW1	0.49	0.28	0.71	0.01	0.05	0.11	0.59	0.95	2.26
cal.Vivo.ABC	NW1	0.40	0.24	0.59	0.01	0.04	0.10	0.48	0.78	1.75
ref.Data	NW2	1.23	0.69	1.66	0.03	0.13	0.27	1.56	2.57	5.60
ref.Vitro	NW2	1.13	0.61	1.58	0.02	0.11	0.23	1.42	2.41	5.36
ref.Vitro.ABC	NW2	1.26	0.67	2.04	0.02	0.12	0.25	1.53	2.58	5.77
cal.Vitro.ABC	NW2	0.79	0.45	1.15	0.01	0.07	0.16	0.98	1.62	3.58
cal.Esl.ABC	NW2	0.48	0.30	0.62	0.01	0.05	0.11	0.62	0.98	2.03
cal.Vivo.ABC	NW2	0.44	0.29	0.57	0.01	0.05	0.12	0.57	0.87	1.82

Metric: shear stress. Unit: dyn/(cm*cm). Segment type: C.										
model	nw	mean	median	sd	q2.5	q12.5	q25	q75	q87.5	q97.5
ref.Data	NW1	27.51	14.31	45.04	0.29	2.23	5.41	32.04	53.76	136.33
ref.Vitro	NW1	29.43	14.57	51.04	0.29	2.24	5.45	33.23	57.99	152.54
ref.Vitro.ABC	NW1	35.32	17.76	56.99	0.46	3.04	6.93	41.14	70.56	179.19
cal.Vitro.ABC	NW1	26.05	12.51	46.60	0.33	1.98	4.68	29.29	51.12	135.02
cal.Esl.ABC	NW1	51.89	27.94	82.05	0.65	4.36	10.87	61.89	100.28	252.26
cal.Vivo.ABC	NW1	78.15	43.71	126.07	0.74	6.18	16.05	93.49	154.56	363.02
ref.Data	NW2	43.66	26.27	54.37	0.91	4.64	10.17	57.29	91.56	183.33
ref.Vitro	NW2	47.03	27.68	62.05	0.91	4.78	10.58	60.97	99.10	199.36
ref.Vitro.ABC	NW2	50.68	29.85	68.22	0.89	5.36	11.68	65.35	104.56	214.94
cal.Vitro.ABC	NW2	24.71	14.46	33.04	0.42	2.25	5.24	32.40	51.12	107.32
cal.Esl.ABC	NW2	52.88	32.98	65.98	1.01	5.45	11.79	70.32	109.20	217.56
cal.Vivo.ABC	NW2	90.97	60.04	105.63	1.56	9.64	22.69	122.78	185.73	355.93

Metric: vel_c. Unit: mm/s. Segment type: C.										
model	nw	mean	median	sd	q2.5	q12.5	q25	q75	q87.5	q97.5
ref.Data	NW1	0.93	0.51	1.42	0.02	0.10	0.21	1.12	1.89	4.47
ref.Vitro	NW1									
ref.Vitro.ABC	NW1									
cal.Vitro.ABC	NW1	1.13	0.57	1.93	0.02	0.10	0.23	1.30	2.24	5.66
cal.Esl.ABC	NW1	0.81	0.45	1.19	0.01	0.08	0.19	0.98	1.57	3.78
cal.Vivo.ABC	NW1	0.54	0.32	0.81	0.01	0.06	0.13	0.65	1.05	2.38
ref.Data	NW2	1.54	0.83	2.15	0.04	0.16	0.32	1.92	3.20	7.32
ref.Vitro	NW2									
ref.Vitro.ABC	NW2									
cal.Vitro.ABC	NW2	1.04	0.59	1.56	0.02	0.10	0.22	1.29	2.14	4.81
cal.Esl.ABC	NW2	0.79	0.47	1.08	0.02	0.08	0.18	1.01	1.62	3.46
cal.Vivo.ABC	NW2	0.59	0.37	0.80	0.01	0.07	0.16	0.76	1.17	2.52

Table 8 (File S2). Summary statistics computed across segment type: C.

q2.5, q12.5, q25, q75, q87.5, q97.5 are distribution percentiles. Refer to the main text for model abbreviations. Hematocrit and RBC velocities are not summarized for the ref.Vitro and ref.Vitro.ABC models, since the biphasic nature of blood was not incorporated into these models (refer to section 2.6.1 in the main text for further details).

A: Iterations at convergence.									
model	network	ω	mean	median	sd	q12.5	q25	q75	q87.5
cal.Vitro.ABC	NW1	0	19.08	19.00	7.28	16.00	17.00	21.00	21.00
cal.Esl.ABC	NW1	0	37.05	32.00	17.69	26.00	28.00	40.00	49.00
cal.Vivo.ABC	NW1	0	63.12	56.00	27.25	41.00	46.00	71.00	91.00
cal.Vitro.ABC.w10	NW1	10	15.70	15.00	4.98	14.00	15.00	16.00	17.00
cal.Esl.ABC.w10	NW1	10	23.12	23.00	0.86	22.00	23.00	24.00	24.00
cal.Vivo.ABC.w10	NW1	10	30.75	30.00	2.61	28.00	29.00	32.00	33.00
cal.Vitro.ABC	NW2	0	18.88	18.00	3.11	16.00	16.00	21.00	23.00
cal.Esl.ABC	NW2	0	37.72	34.00	14.30	28.00	30.00	41.00	50.00
cal.Vivo.ABC	NW2	0	70.20	64.00	25.46	49.00	53.00	80.25	93.00
cal.Vitro.ABC.w10	NW2	10	15.88	15.00	2.23	14.00	14.00	17.00	19.00
cal.Esl.ABC.w10	NW2	10	24.70	24.00	2.79	23.00	24.00	25.00	26.00
cal.Vivo.ABC.w10	NW2	10	33.43	32.00	3.47	31.00	31.00	35.00	37.00

B: Diverging bifurcations below threshold ϕ_3 at convergence.									
model	network	ω	mean	median	sd	q12.5	q25	q75	q87.5
cal.Vitro.ABC	NW1	0	3104.08	3104.00	14.52	3088.00	3094.75	3114.00	3121.00
cal.Esl.ABC	NW1	0	3087.58	3087.00	13.98	3071.00	3078.00	3097.00	3104.00
cal.Vivo.ABC	NW1	0	3075.02	3074.00	13.98	3059.00	3066.00	3084.00	3092.00
cal.Vitro.ABC.w10	NW1	10	3104.02	3104.00	14.50	3088.00	3094.00	3114.00	3121.00
cal.Esl.ABC.w10	NW1	10	3087.58	3087.00	13.98	3071.00	3078.00	3097.00	3104.00
cal.Vivo.ABC.w10	NW1	10	3075.03	3074.00	13.98	3059.00	3066.00	3084.00	3092.00
cal.Vitro.ABC	NW2	0	5398.49	5398.00	18.92	5377.00	5386.00	5411.00	5421.00
cal.Esl.ABC	NW2	0	5365.72	5366.00	18.01	5345.00	5353.00	5378.00	5387.00
cal.Vivo.ABC	NW2	0	5344.62	5344.00	16.72	5325.00	5334.00	5356.00	5364.00
cal.Vitro.ABC.w10	NW2	10	5398.49	5398.00	18.92	5377.00	5386.00	5411.00	5421.00
cal.Esl.ABC.w10	NW2	10	5365.72	5366.00	18.00	5345.00	5353.00	5378.00	5387.00
cal.Vivo.ABC.w10	NW2	10	5344.63	5344.00	16.72	5325.00	5334.00	5356.00	5364.00

C: Diverging bifurcations above threshold ϕ_3 at convergence.									
model	network	ω	mean	median	sd	q12.5	q25	q75	q87.5
cal.Vitro.ABC	NW1	0	0.01	0.00	0.09	0.00	0.00	0.00	0.00
cal.Esl.ABC	NW1	0	0.00	0.00	0.07	0.00	0.00	0.00	0.00
cal.Vivo.ABC	NW1	0	0.00	0.00	0.03	0.00	0.00	0.00	0.00
cal.Vitro.ABC.w10	NW1	10	0.01	0.00	0.08	0.00	0.00	0.00	0.00
cal.Esl.ABC.w10	NW1	10	0.00	0.00	0.07	0.00	0.00	0.00	0.00
cal.Vivo.ABC.w10	NW1	10	0.00	0.00	0.03	0.00	0.00	0.00	0.00
cal.Vitro.ABC	NW2	0	0.03	0.00	0.16	0.00	0.00	0.00	0.00
cal.Esl.ABC	NW2	0	0.01	0.00	0.08	0.00	0.00	0.00	0.00
cal.Vivo.ABC	NW2	0	0.01	0.00	0.13	0.00	0.00	0.00	0.00
cal.Vitro.ABC.w10	NW2	10	0.03	0.00	0.16	0.00	0.00	0.00	0.00
cal.Esl.ABC.w10	NW2	10	0.01	0.00	0.08	0.00	0.00	0.00	0.00
cal.Vivo.ABC.w10	NW2	10	0.01	0.00	0.14	0.00	0.00	0.00	0.00

Table 9 (File S2). Flow iterations, summary statistics
q12.5, q25, q75, q87.5 are distribution percentiles, and sd is standard deviation.
Refer to sections 3.3 and 4 in File S1 for information about ϕ_3 and ω, respectively.
Refer to the main text for model abbreviations.
