# Supplementary material for: Blood Flow Simulation and Uncertainty Quantification in Extensive Microvascular Networks: Application to Brain Cortical Networks
Source: Microcirculation. 2025 Sep 21;32(7):e70027. doi: 10.1111/micc.70027 (PMC12450458; doi:10.1111/micc.70027)
Supplement: Supplementary file 3 — File S3: micc70027‐sup‐0003‐FileS3.pdf. [file MICC-32-e70027-s003.pdf]

Blood flow simulation and uncertainty quantification in extensive microvascular networks: Application to brain cortical networks

File S3
Supplementary figures

2025.08.19

Peter Mondrup Rasmussen[1*]
1 Center of Functionally Integrative Neuroscience, Department of Clinical Medicine, Aarhus University, Aarhus, Denmark. * Corresponding author: pmr@cfin.au.dk.

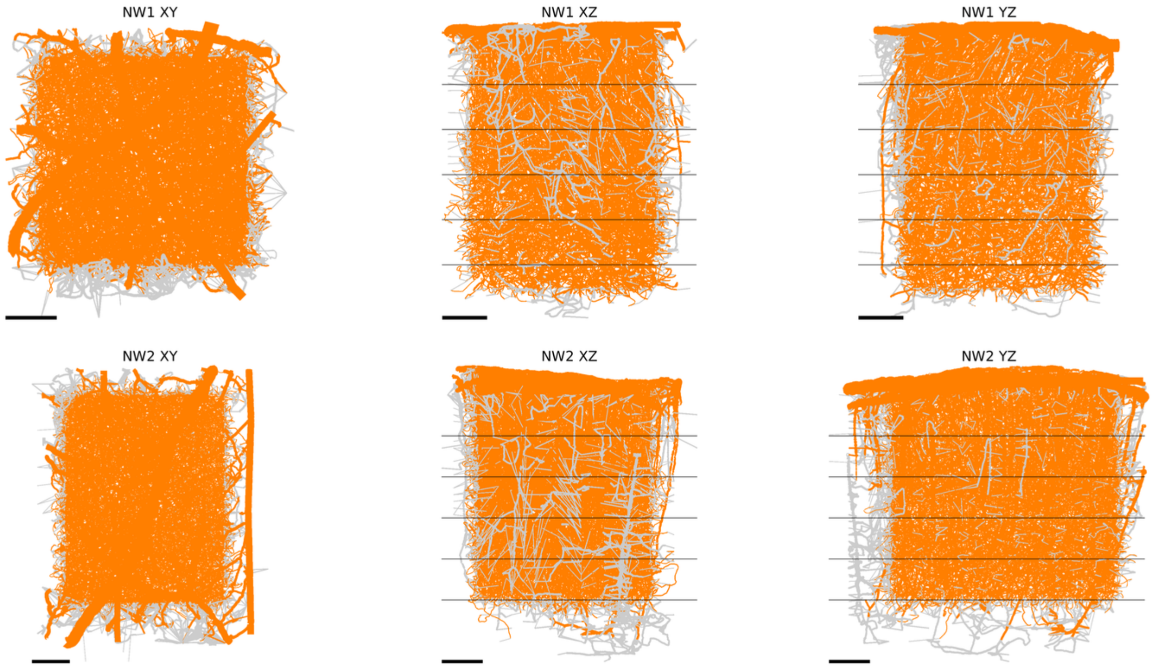

Figure 1 (File S3): The two studied microvascular networks (NW1 and NW2) were obtained from two literature sources [1, 2]. The most recent version of the networks provides a cleaner representation near the network faces with depth. Consequently, the networks from the original source were trimmed to match the more recent version by identifying vessel segments present in both data sets. Segments unique to the original networks were removed, provided that their removal did not alter the degree order of interior nodes. This facilitates the use of hemodynamic simulation results, including pressure boundary conditions and hematocrit, from the original data source while utilizing the cleaner, more recent spatial representation. The orange color represents the resulting trimmed networks, while the union of gray and orange colors represents the original networks. Networks are shown in rows, and different 2D projections are shown in columns. Boundaries between analysis layers are shown as horizontal lines in the XZ and YZ projections. Scale bars represent 200 μm.

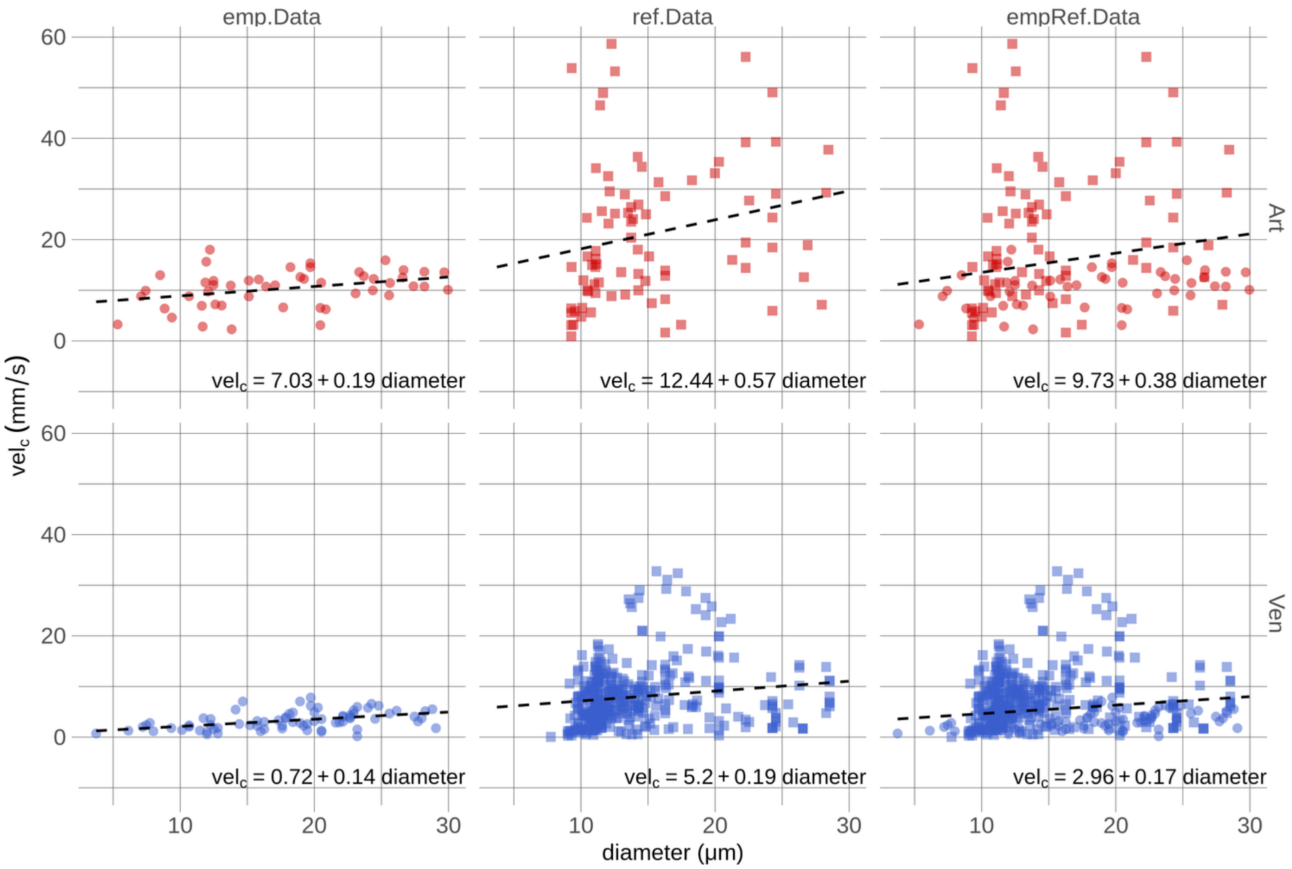

Figure 2 (File S3): Diameter dependent target values used in the Bayesian model calibration in Experiment 2. Linear functions were used to create target values for each of the vessel categories arterioles and venules, represented in rows. The left column shows empirical measurements of red blood cell (RBC) velocities in mice obtained from a literature source[3], denoted emp.Data. The middle column shows simulated RBC velocities in the two studied microvascular networks obtained from the reference dataset[1], denoted ref.Data. The right column shows the two data sets combined. Target velocities were established based on data points up to a diameter of 30 μm, corresponding to the approximate range with data points available for both vessel categories in the empirical data[3]. Dashed lines in the first two columns represent linear fits. Dashed lines in the right column represent averages of the linear fits in the first two columns. The individual fits to emp.Data and ref.Data were averaged, instead of fitting a function to the combined data, to ensure equal contribution of the two data sources given the fact that the distribution of data points across the diameter span differs for the two data sources.

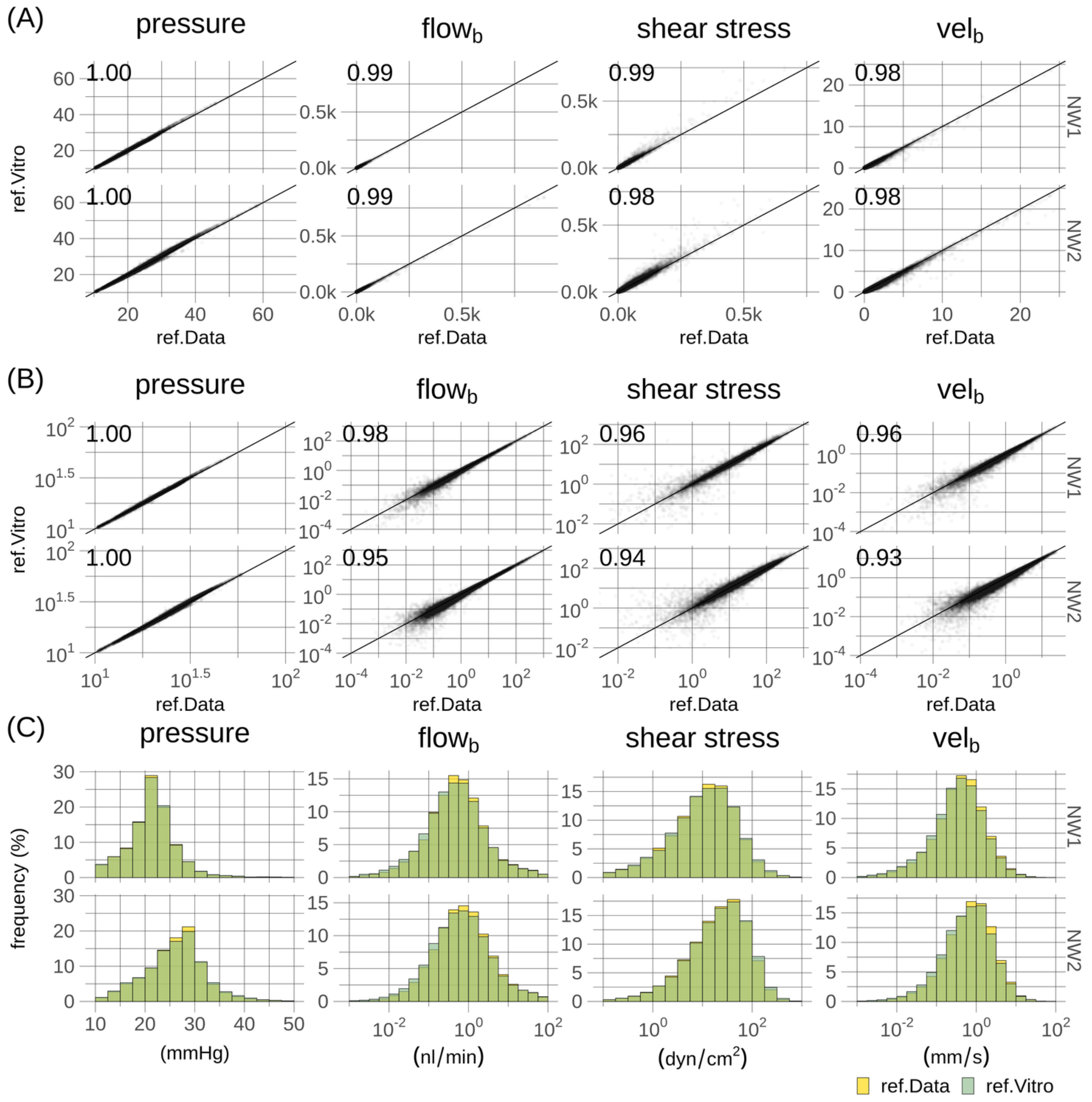

Figure 3 (File S3): Quantitative comparison of hemodynamic metrics in the reference data (ref.Data) and in the reference simulation (ref.Vitro) which utilized pressure boundary conditions from the reference data. Shear stress for ref.Data was calculated using segment lengths, diameters, and pressure drops. Points are shown with transparency to facilitate representation of point distributions. The scatter plots are shown on a log-scale in (B). Black lines represent identity lines, and correlation coefficients (Spearman's for (A) and Pearson for (B)) are provided as inserts. Physical units of axes in (A) and (B) are consistent with the axis units of frequency histograms depicted in (C). Strong correspondence between reference simulations and the reference data is observed. A minor level of scatter is observed for a smaller number of points. In interpreting this scatter, it should be noted that ref.Data represent averaged values from blood flow simulations based on a numerical model with discrete tracking of red blood cells[1]. The ref.Vitro simulations utilized such average pressures as pressure boundary conditions and also utilized average hematocrits for calculating effective viscosities used in simulations in Experiment 1. As viscosity depends non-linearly on hematocrit, exact numerical agreement between ref.Data and ref.Vitro simulations is not expected. Despite this, a strong agreement is observed both in the scatter plots in (A) and (B), and a strong congruency between frequency histograms is seen in (C), supporting the validity of the ref.Vitro simulations.

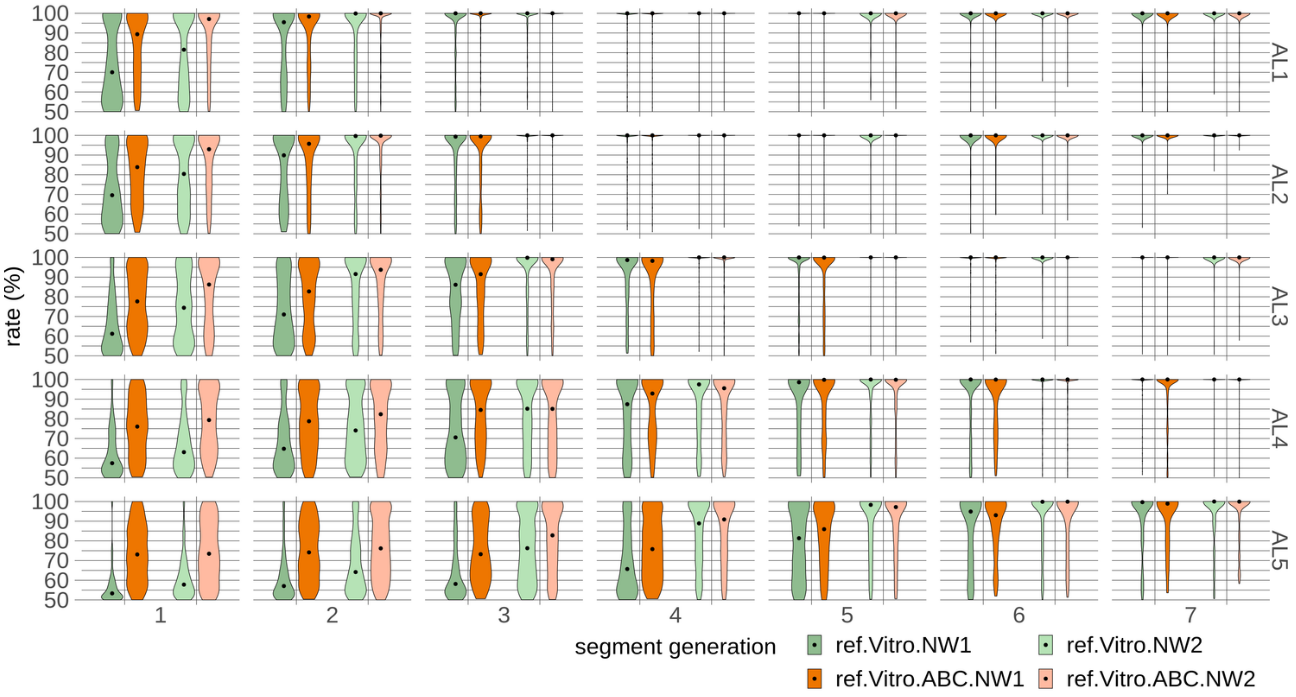

Figure 4 (File S3): Depth-wise (AL) and segment generation-wise profiles of direction agreement rate for different combinations of the two reference models (ref.Vitro and ref.Vitro.ABC) and the two microvascular networks (NW1 and NW2). Different combinations of models and networks are represented by unique colors. Violins represent distributions over segments, and dots mark medians.

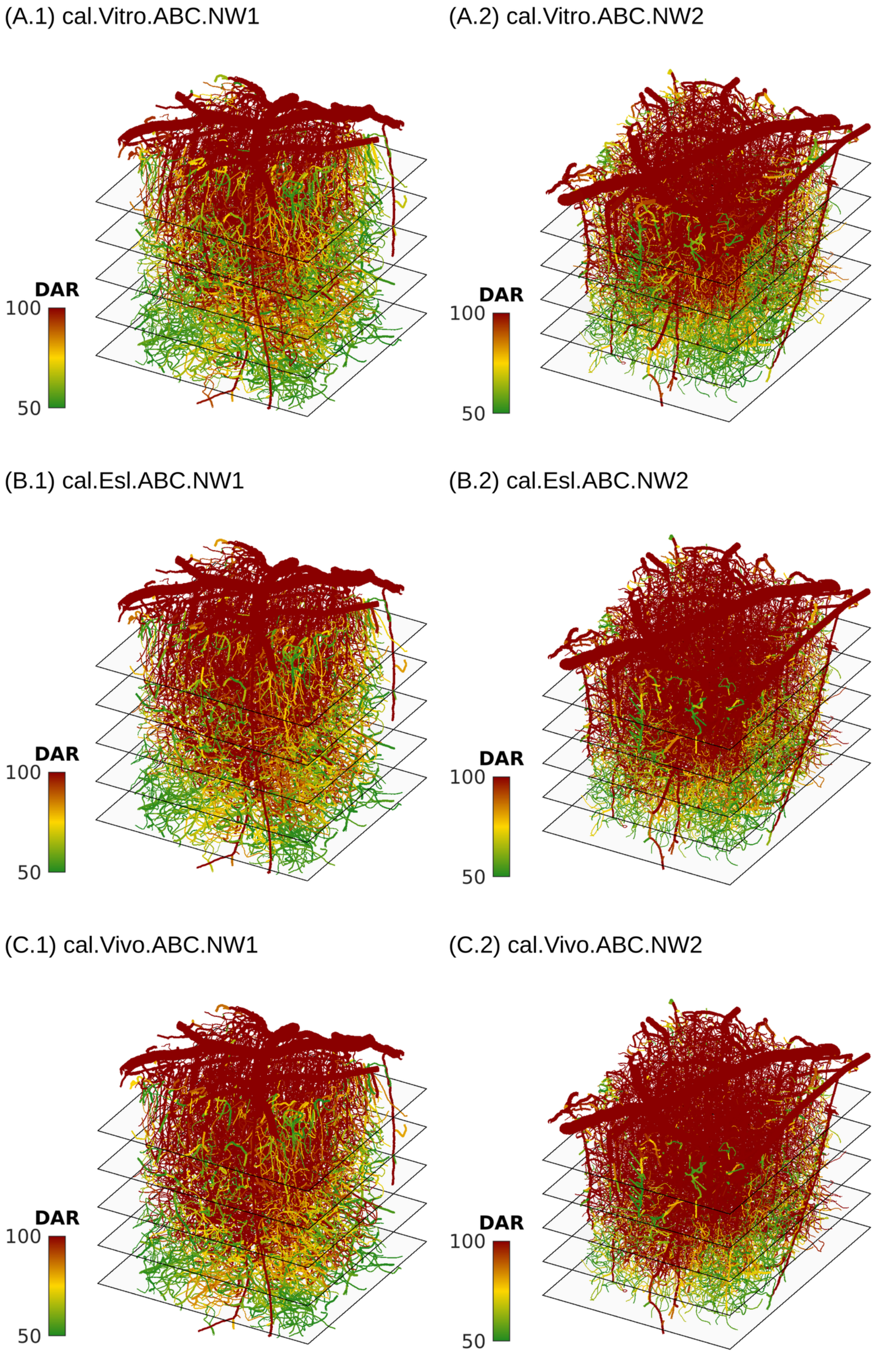

Figure 5 (File S3): Spatial representation of direction agreement rates (DAR) for all three calibration models in both microvascular networks. Each segment in the spatial representations of DAR is uniquely colored according to its rate. A direction agreement rate of 100% corresponds to full agreement in blood flow direction, while a rate of 50% corresponds to complete disagreement.

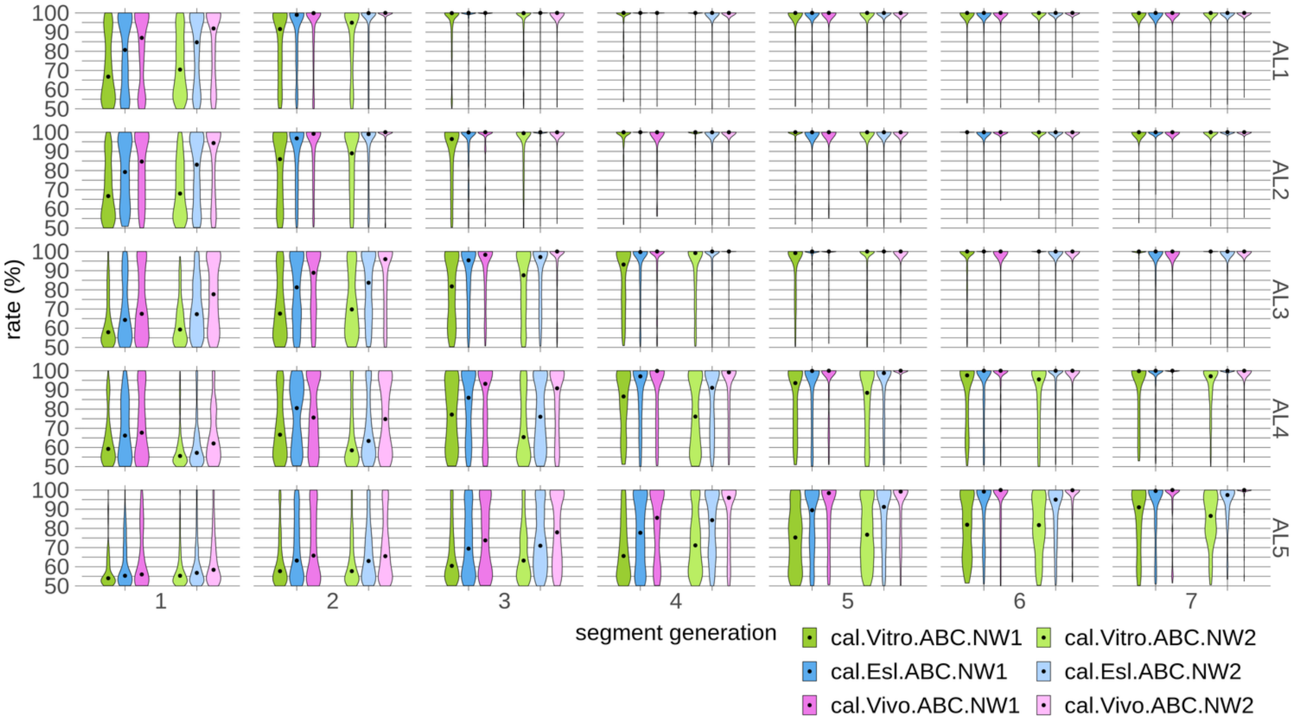

Figure 6 (File S3): Depth-wise (AL) and segment generation-wise profiles of direction agreement rate for different combinations of the three calibration models and the two microvascular networks (NW1 and NW2). Different combinations of models and networks are represented by unique colors. Violins represent distributions over segments, and dots mark medians.

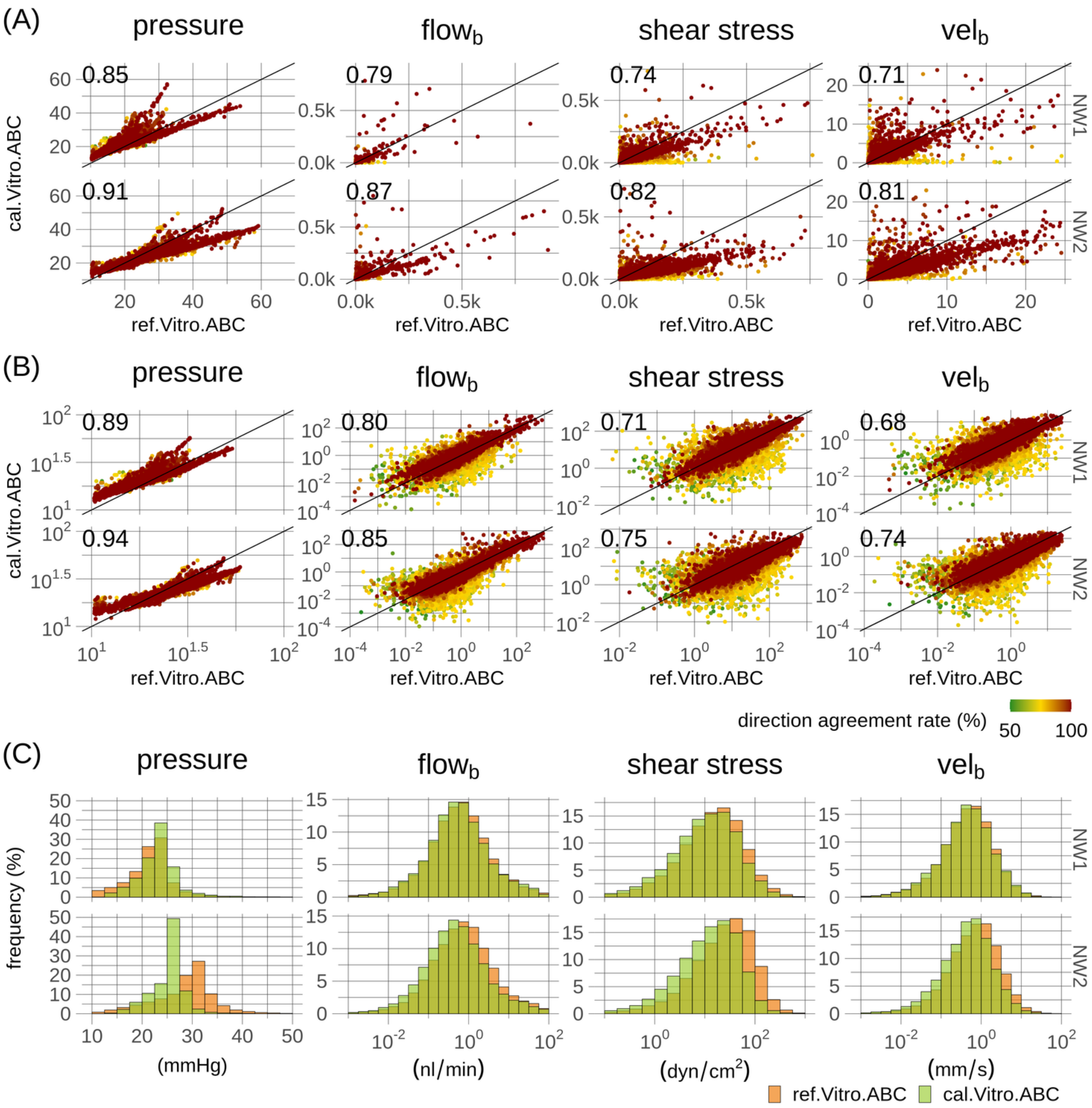

Figure 7 (File S3): Quantitative comparison of hemodynamic metrics in the ref.Vitro.ABC and cal.Vitro.ABC models. In (A) and (B), each point in the scatter plots represents a vessel segment and is uniquely colored according to its direction agreement rate (DAR) averaged across the two models. For better visualization, scatter points are overlaid according to increasing DAR. The scatter plots are shown on a log-scale in (B). Black lines represent identity lines, and correlation coefficients, Spearman's for (A) and Pearson for (B), are provided as inserts. Physical units of axes in (A) and (B) are consistent with the axis units of frequency histograms depicted in (C). Rows in (A-C) correspond to the two microvascular networks (NW1 and NW2).

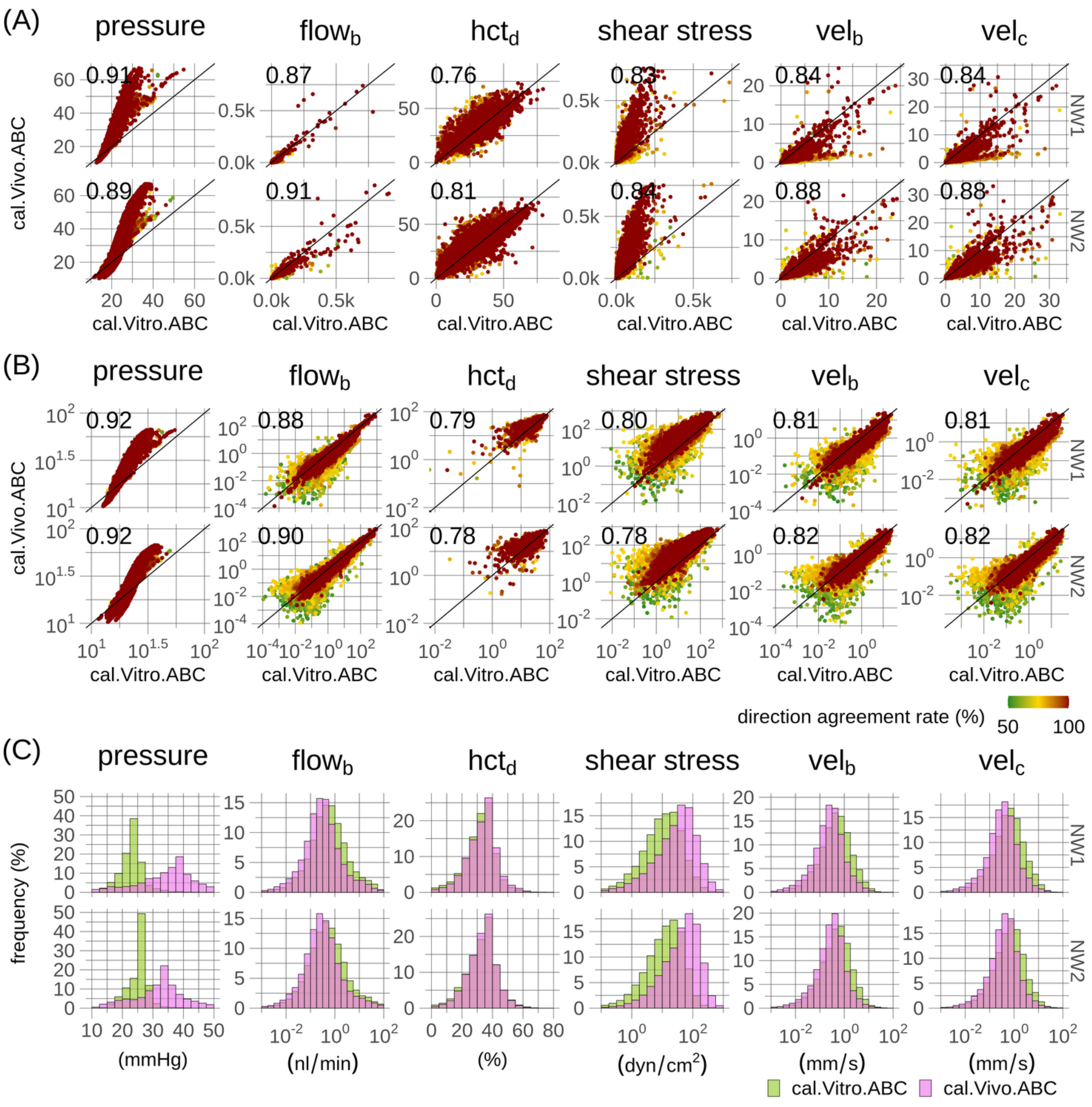

Figure 8 (File S3): Same format as in Figure 7 in File S3 but for the cal.Vitro.ABC and cal.Vivo.ABC model pair.

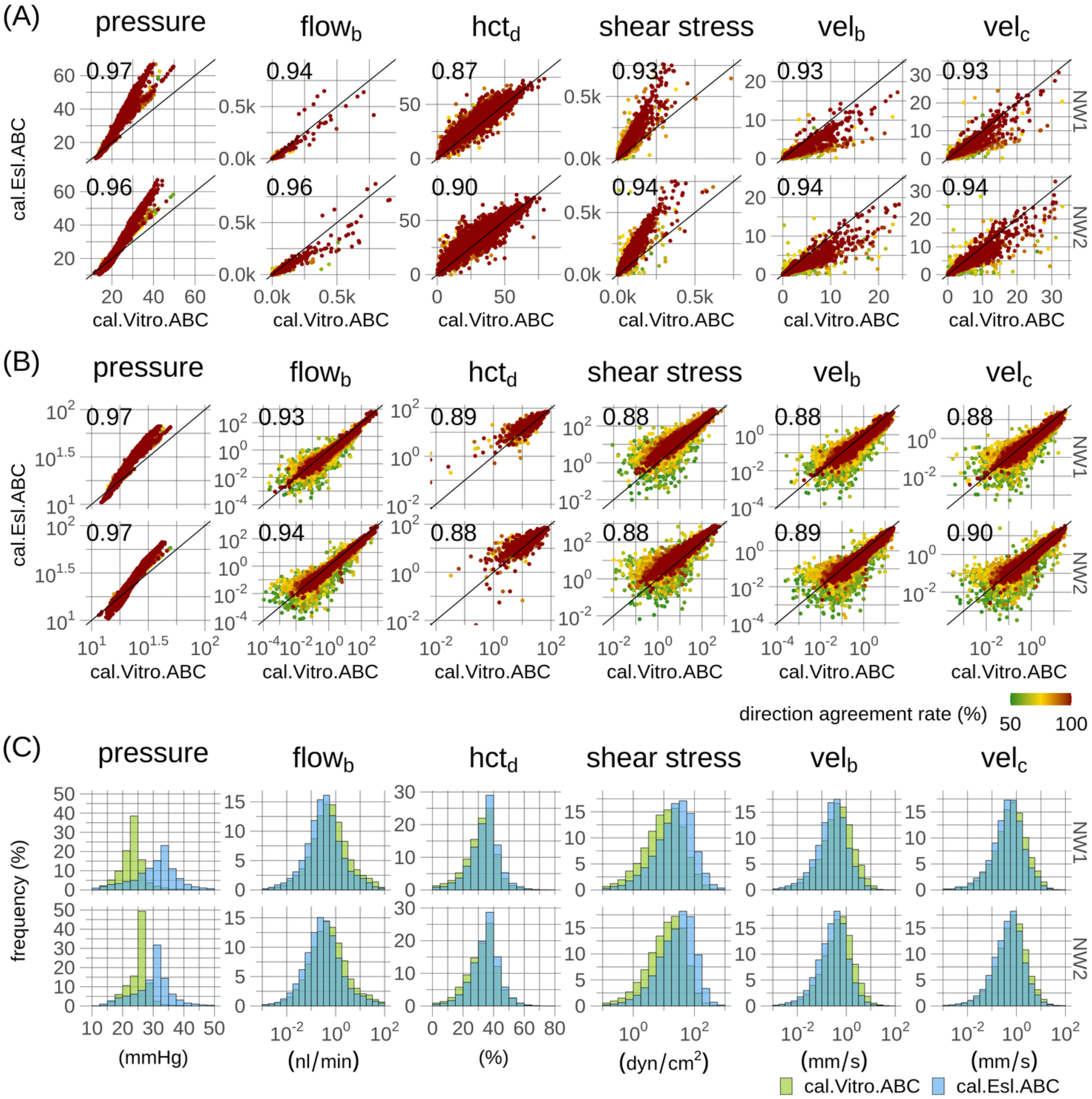

Figure 9 (File S3): Same format as in Figure 7 in File S3 but for the cal.Vitro.ABC and cal.Esl.ABC model pair.

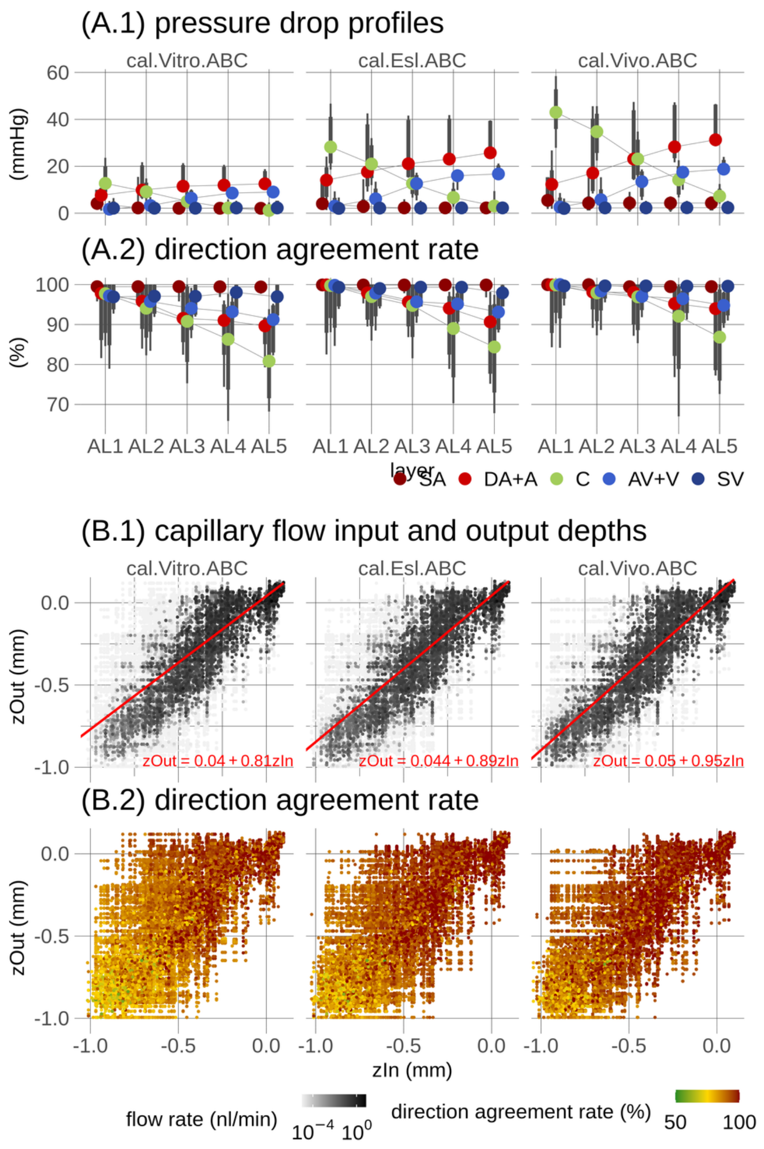

Figure 10 (File S3): Pressure drop profiles and capillary input and output depths along pathways of red blood cell (RBC) flow. In (A.1), the pressure drop profiles for each vessel category are shown. The paths of RBC flow, which were tracked from the end nodes of surface arterioles to surface venules, are grouped by analysis layers (ALs) based on the depth of the first capillary segment along the paths. The columns display the three calibration models. Points denote medians across paths, while the thick and thin vertical lines represent [12.5 87.5] and [5 95] percentiles, respectively. (A.2) show a corresponding uncertainty analysis, with uncertainty quantified by average direction agreement rate of segments across respective parts of individual flow paths. In (B.1), the depths of capillary inputs (zIn) and outputs (zOut) are presented. Individual paths are represented by points, with the intensity of the greyscale color indicating the RBC flow rate. For better visualization, colors are displayed on a log-scale, and points are ordered according to intensity. Inserts provide linear fits and their corresponding equations. (B.2) shows direction agreement rates corresponding to points in (B.1) using the same point ordering. The summary statistics in (A.1 and A.2) and the fits in (B.1) were computed by weighting individual paths according to their RBC flow rate. SA: surface arteriole, DA: descending arteriole, A: arteriole, C: capillary, V: venule, AV: ascending venule, SV: surface venule.

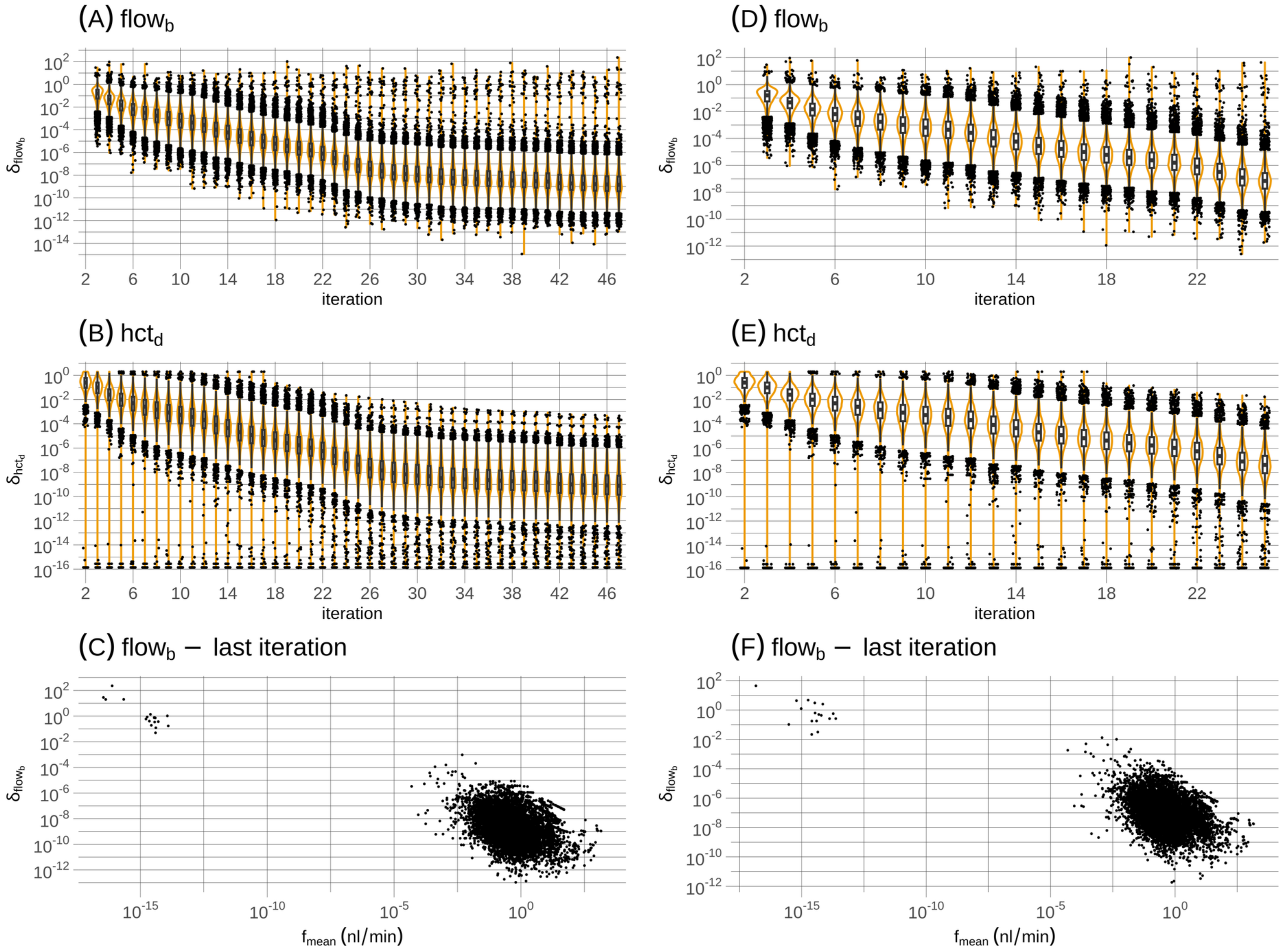

Figure 11 (File S3): Illustration of hematocrit iterations of the biphasic simulation model cal.Esl.ABC in Experiment 2 (the example shown is in microvascular network NW1 comprising 10873 vessel segments). Iterations are shown for a specific set of pressure boundary conditions, corresponding to one sample from the retained Markov chain Monte Carlo chain samples (semi-randomly selected sample index 42). (A) and (D) show relative changes in segment blood flow, (B) and (E) show relative changes in segment hematocrit, and (C) and (F) show relative changes in segment blood flow vs. blood flow at the last iteration. The left column (A-C) is based on simulations with no super-threshold segments allowed ($\omega = 0$), whereas 10 super-threshold segments were allowed ($\omega = 10$) in the right column (D-F). Convergence threshold was $\epsilon = 10^{-3}$. Violins represent distributions across segments. Boxplots in (A,B,D,E) show the median as a line within the box, the first and third quartiles as the edges of the box, and the interquartile range (IQR) as the distance between these quartiles. Whiskers extend to the smallest and largest values within 1.5 times the IQR from the quartiles, while individual segments outside the whiskers are indicated by dots. The cloud with a few segments at very low blood flow in (C) and (F) corresponds to interior dead-end loops.

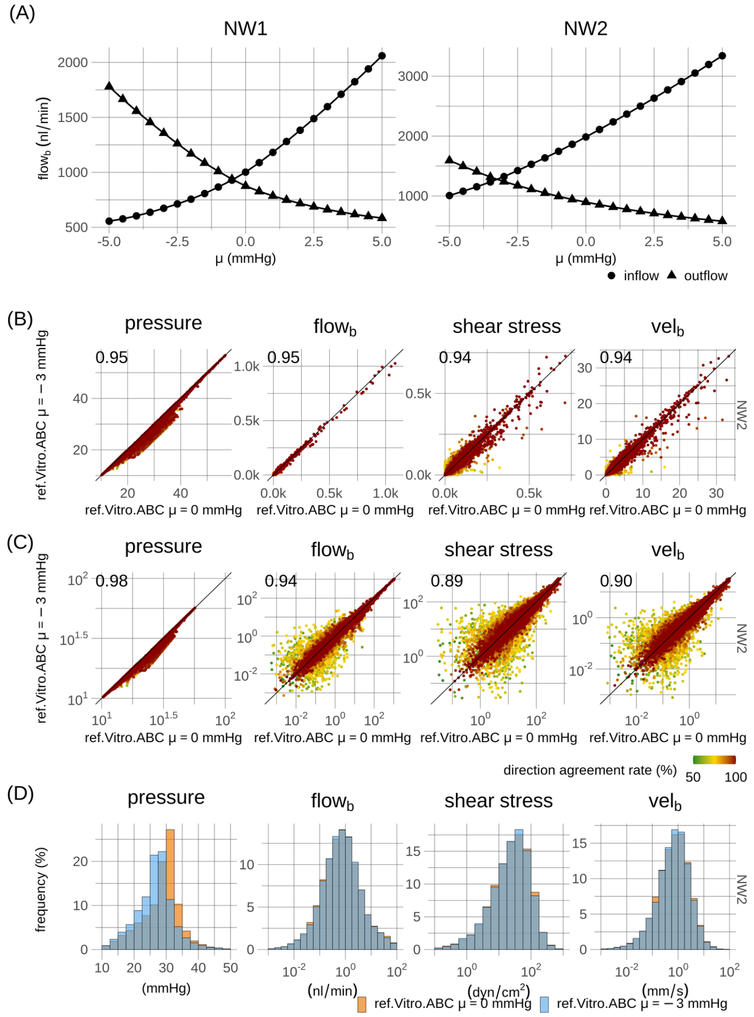

Figure 12 (File S3): Capillary blood inflow and outflow rates accumulated across network boundary faces in variants of the ref.Vitro.ABC model. In the analyses presented in the main text, pressure deviations (Δp, see Eq. 3, main text) were defined as random variables centered at zero mean ($\mu = 0$ mmHg) relative to average reference node pressures (Sections 2.6.1 and 2.6.2, main text). Panel (A) illustrates how the accumulated capillary boundary inflows and outflows in the ref.Vitro.ABC model vary when biasing pressure deviations toward venous or arteriolar pressures by globally varying μ. Approximate flow balance was achieved at $\mu = -0.5$ mmHg (resp. $\mu = -3.0$ mmHg) in NW1 (resp. NW2). Panels (B)–(D) (same format as Figure 7 in File S3) compare hemodynamic metrics between the default and balanced model variants in NW2, which exhibited the largest difference between its default and balanced variant. While biasing pressure deviations improved flow balance, the overall hemodynamic metrics remained largely consistent across models. Histograms of hemodynamic variables showed close alignment (D), with only the pressure distributions differing, as expected due to the negatively shifted boundary pressure deviations. While the flexibility of the adaptive method facilitates incorporation of flow balancing across network boundary faces as an additional modeling constraint, depending on network architecture and modeling assumptions, these results suggest that the adaptive method yields hemodynamic predictions that are robust to moderate global deviations in relative pressure settings and differences between accumulated blood inflows and outflows.

1 References

References

[1] F. Schmid, P. S. Tsai, D. Kleinfeld, P. Jenny, and B. Weber. Depth-dependent flow and pressure characteristics in cortical microvascular networks. *PLoS Comput Biol*, 13(2):e1005392, 2017.

[2] F. Schmid, G. Conti, P. Jenny, and B. Weber. The severity of microstrokes depends on local vascular topology and baseline perfusion. *Elife*, 10, 2021.

[3] Z. J. Taylor, E. S. Hui, A. N. Watson, X. Nie, R. L. Deardorff, J. H. Jensen, J. A. Helpern, and A. Y. Shih. Microvascular basis for growth of small infarcts following occlusion of single penetrating arterioles in mouse cortex. *J Cereb Blood Flow Metab*, 36(8):1357–73, 2016.
